# Computerized clinical decision support systems for chronic disease management: A decision-maker-researcher partnership systematic review

**DOI:** 10.1186/1748-5908-6-92

**Published:** 2011-08-03

**Authors:** Pavel S Roshanov, Shikha Misra, Hertzel C Gerstein, Amit X Garg, Rolf J Sebaldt, Jean A Mackay, Lorraine Weise-Kelly, Tamara Navarro, Nancy L Wilczynski, R Brian Haynes

**Affiliations:** 1 Health Research Methodology Program, McMaster University, 1280 Main Street West, Hamilton, ON, Canada; 2 University of Toronto, 1 Kings College Circle, Toronto, ON, Canada; 3 Department of Medicine, McMaster University, 1280 Main Street West, Hamilton, ON, Canada; 4 Hamilton Health Sciences, 1200 Main Street West, Hamilton, ON, Canada; 5 Department of Medicine, Department of Epidemiology and Biostatistics, University of Western Ontario, 1151 Richmond Street, London, ON, Canada; 6 Health Information Research Unit, Department of Clinical Epidemiology and Biostatistics, McMaster University, 1280 Main Street West, Hamilton, ON, Canada

## Abstract

**Background:**

The use of computerized clinical decision support systems (CCDSSs) may improve chronic disease management, which requires recurrent visits to multiple health professionals, ongoing disease and treatment monitoring, and patient behavior modification. The objective of this review was to determine if CCDSSs improve the processes of chronic care (such as diagnosis, treatment, and monitoring of disease) and associated patient outcomes (such as effects on biomarkers and clinical exacerbations).

**Methods:**

We conducted a decision-maker-researcher partnership systematic review. We searched MEDLINE, EMBASE, Ovid's EBM Reviews database, Inspec, and reference lists for potentially eligible articles published up to January 2010. We included randomized controlled trials that compared the use of CCDSSs to usual practice or non-CCDSS controls. Trials were eligible if at least one component of the CCDSS was designed to support chronic disease management. We considered studies 'positive' if they showed a statistically significant improvement in at least 50% of relevant outcomes.

**Results:**

Of 55 included trials, 87% (n = 48) measured system impact on the process of care and 52% (n = 25) of those demonstrated statistically significant improvements. Sixty-five percent (36/55) of trials measured impact on, typically, non-major (surrogate) patient outcomes, and 31% (n = 11) of those demonstrated benefits. Factors of interest to decision makers, such as cost, user satisfaction, system interface and feature sets, unique design and deployment characteristics, and effects on user workflow were rarely investigated or reported.

**Conclusions:**

A small majority (just over half) of CCDSSs improved care processes in chronic disease management and some improved patient health. Policy makers, healthcare administrators, and practitioners should be aware that the evidence of CCDSS effectiveness is limited, especially with respect to the small number and size of studies measuring patient outcomes.

## Background

Chronic conditions present patients, practitioners, and healthcare systems with some unique demands, including recurrent visits, adherence to complex care plans, long-term disease and treatment monitoring, behavior modification, and patient self-management. For the many patients with multiple co-morbidities [1], overlapping or diverging care plans may further complicate these processes.

Computerized clinical decision support systems (CCDSSs) may help practitioners meet the requirements of chronic care. These systems analyze a patient's characteristics to provide tailored recommendations for diagnosis, treatment, patient education, adequate follow-up, and timely monitoring of disease indicators. For example, Holbrook *et al*. [2,3] gave providers and diabetic patients access to a web-based system that offered care advice, allowed monitoring of diabetes risk factors, and tracked key care targets. As with any health intervention, however, rigorous testing is warranted to determine whether CCDSSs improve chronic care processes and patient outcomes.

In our previous review of the effects of CCDSSs [4], we analyzed 100 randomized and non-randomized studies published until September 2004, 40 of which assessed the effects of CCDSSs on disease management. Of these 40 studies, 37 measured processes of care of which 62% (23) showed an improvement, and 27 measured patient outcomes of which 19% (5) showed an improvement. The quality of the studies varied widely, but improved over time.

Many new randomized controlled trials (RCTs) have been published in this field since our previous work, potentially documenting important advances. Recognizing that the management of chronic disease has unique characteristics, we wished to review the impact of CCDSSs on the quality and effectiveness of chronic care. We had the opportunity to include the perspectives of senior hospital managers and front-line healthcare practitioners to ensure that relevant data were extracted and summarized--a level of stakeholder engagement that has not been included in other reviews [5-8].

## Methods

We previously published the details of our review protocol, openly accessible at http://www.implementationscience.com/content/5/1/12[9]. These methods are briefly summarized here, along with details specific to this review of CCDSSs for chronic disease management.

### Research question

Do CCDSSs improve chronic disease management processes or patient outcomes?

### Partnering with decision makers

We conducted this review in partnership with individuals responsible for implementing CCDSSs in our region [9]. Decision makers, both managers and clinicians, met with the review team periodically to discuss direction and specific details for the data extraction, analysis, presentation and interpretation of results.

### Search strategy

Full details of our search strategy are in our review protocol [9]. In summary, we searched MEDLINE, EMBASE, Ovid's Evidence-Based Medicine Reviews, and Inspec until 6 January 2010, and reviewed the reference lists of included RCTs and relevant systematic reviews. We screened articles for eligibility in two stages: a duplicate, independent review of titles and abstracts followed by a duplicate, independent, full-text review of potentially eligible articles, with a third reviewer resolving disagreements.

### Study selection

We selected RCTs of a CCDSS used by a health care provider for management of chronic conditions, published up to 6 January 2010 in any language that measured CCDSS impact on processes of care or patient outcomes. We included RCTs in any language that compared patient care with a CCDSS to routine care without a CCDSS and evaluated clinical performance (*i.e*., a measure of process of care) or a patient outcome. Additionally, to be included in the review, the CCDSS had to provide patient-specific advice that was reviewed by a healthcare practitioner before any clinical action. Studies were excluded if the system was used solely by students, only provided summaries of patient information, provided feedback on groups of patients without individual assessment, only provided computer-aided instruction, or was used for image analysis. Trials included in our previous review [4] were included if they were eligible. Trials of CCDSSs for managing narrow therapeutic index medications used in some chronic conditions (such as warfarin in atrial fibrillation [10]) were not included in this review, but are discussed in our review for therapeutic drug monitoring and dosing.

### Data extraction

To meet the needs of our management and clinical partners, we extracted study characteristics (*e.g*., study design, size, setting, authorship, funding, and year of publication) and system characteristics (*e.g*., integration with other systems, user interface elements, methods of data entry and delivery of recommendations, target users, and implementation details such as pilot testing and user training). Disagreements were resolved by a third reviewer or by consensus. We contacted primary authors to provide missing data and to assess the accuracy of the extracted data; 78% (43/55) provided input. For the remaining trials, a trained reviewer assessed the extraction form against the full-text to confirm accuracy.

### Assessment of study quality

Using a 10-point scale, pairs of reviewers independently evaluated the selected trials on five dimensions of quality, including concealment of allocation, appropriate unit of allocation, appropriate adjustment for baseline differences, appropriate blinding of assessment, and adequate follow-up [9]. We used a 2-tailed Mann-Whitney U test to compare methodologic scores between trials published before the year 2000 and those published later to determine if trial quality has improved with time.

### Assessment of CCDSS intervention effects

We assessed the effectiveness of CCDSSs in each trial for improving process of care and patient outcomes. We defined process outcomes as changes in care activities such as diagnosis, treatment, and monitoring of disease. Examples of patient outcomes included changes in blood pressure, clinical events and health-related quality of life. We judged a CCDSS effective if it produced a statistically significant (*p *< 0.05) improvement in a primary chronic disease outcome or in ≥50% of multiple relevant pre-specified outcomes. We considered primary any outcome that trial reports described as 'primary' or 'main.' If authors did not designate a primary outcome, we considered the outcome used to calculate the trial's sample size to be primary, if reported. When there were no pre-specified outcomes, the system was considered effective if it produced an improvement in ≥50% of all reported chronic disease outcomes. Our assessment criteria are more specific than those used in our 2005 review [4]; therefore, the assignment of effect was adjusted for some trials included in the review.

### Data synthesis and analysis

We summarized data using proportions, medians, and ranges. Denominators vary in some proportions because not all trials reported relevant information. All analyses were carried out using SPSS, version 15.0. We did not attempt a meta-analysis because of study-level differences in participants, clinical settings, disease conditions, interventions, and outcomes measured.

We conducted a sensitivity analysis to assess the possibility of biased results in studies with a mismatch between the unit of allocation (*e.g*., clinicians) and the unit of analysis (*e.g*., individual patients without adjustment for clustering). We compared success rates between studies with matched and mismatched analyses using chi-square for comparisons. No differences in reported success were found for either process of care outcomes (Pearson X^2 ^= 1.41, *p *= 0.24) or patient outcomes (Pearson X^2 ^= 1.45, *p *= 0.23). Accordingly, results have been reported without distinction for mismatch.

## Results

**Figure 1:**
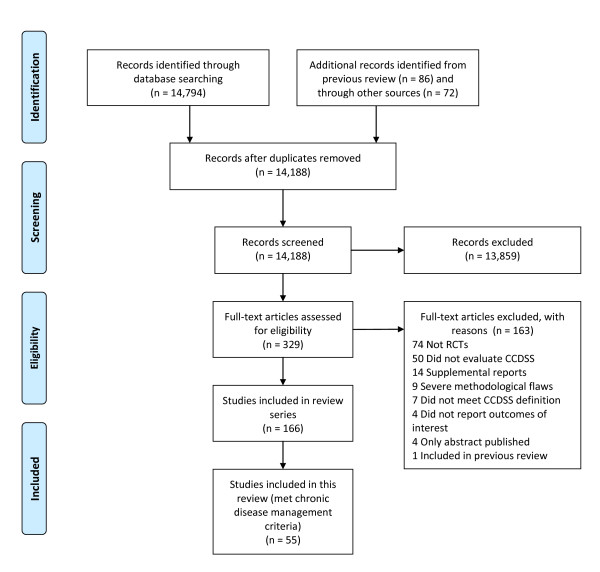
Flow diagram of included and excluded studies for the update 1 January 2004 to 6 January 2010 with specifics for chronic disease management*. * Details provided in: Haynes RB *et al*. [9] Two updating searches were performed, for 2004 to 2009 and to 6 January 2010 and the results of the search process are consolidated here.

Figure 1 shows a flow diagram of included and excluded trials. We identified 166 trials of CCDSSs and Cohen's κ for reviewer agreement on trial eligibility was 0.93 (95% confidence interval [CI], 0.91 to 0.94). In this review, we included 71 publications describing 55 trials (33% of total) about management of chronic diseases [2,3,11-79]. Thirty-eight included studies contributed outcomes to both this review and other CCDSS interventions in the series; three studies [30,53,62] to four reviews, 12 studies [21,25,28,31-33,42-44,51,52,54,55,57-61,74] to three reviews, and 23 studies [2,3,11,12,18,19,23,27,35-38,40,41,45,46,48-50,56,67,71-73,77,79] to two reviews; but we focused here on outcomes relevant to the management of chronic disease.

Summary of trial quality is reported in Additional file 1, Table S1; system characteristics in Additional file 2, Table S2; study characteristics in Additional file 3, Table S3; outcome data in Table 1 and Additional file 4, Table S4; and other CCDSS-related outcomes in Additional file 5, Table S5.

**Table 1:** Results for CCDSS trials of chronic disease management

Study	Methods Score	Indication	No. of centres/providers/patients	Process of care outcomes	CCDSS Effect^a^	Patient outcomes	CCDSS Effect^a^
**Diabetes**							

Holbrook, 2009[2,3]	7	Web-based tracking of diabetes monitoring in adults in primary care.	18/46/511*	Measurement of HbA_1c_, BP, LDL-C, albuminuria, BMI, exercise, and smoking status; foot surveillance.	**+**	Levels of BP, LDL-C, HbA_1c_, and albuminuria; BMI, exercise rate, absence of foot neuropathy and smoking; quality of life.	**+**
Maclean, 2009[11,12]	8	Reminders for the management of diabetes in primary care.	64*/132/7,412	Test completion within guideline-specified times (HbA_1c_, lipids, serum creatinine, and urine microalbumin).	**+**	Mean HbA_1c _level; patients with HbA_1c _<7%.	**0**
Christian, 2008[13]	8	Patient feedback and physician recommendations for management obesity and type 2 diabetes in primary care.	2/19/273*	...	**..**.	Weight change; patients with ≥5% weight loss.	**+**
Cleveringa2008 [14-17]	6	Recommendations for management of type 2 diabetes in primary care.	55*/.../3,391	Diabetes treatment satisfaction score.	**0**	Mean HbA_1c_.	**0**
Peterson, 2008[18]	10	Visit reminders and patient-specific physician alerts and progress reports for organization of primary care in patients with type 2 diabetes.	24*/238/7,101	Completion of foot and eye exams, BP monitoring, and renal, HbA_1c_, and LDL-C tests.	**+**	Patients with target composite clinical outcome (SBP <130 mm Hg, HbA_1c _<7%, and LDL-C <100 mg/dL).	**+**
Quinn, 2008[19]	6	Cell phone-based type 2 diabetes management, with real-time coaching for patients and remote monitoring of blood glucose for practitioners in primary care.	3/26/30*	Medications intensified and medication errors identified.	**+**	Mean HbA_1c_.	**+**
Augstein, 2007[20]	8	Recommendations for management of diabetes in outpatients.	5/5/49*	...	**..**.	Change in HbA_1c _and glucose levels.	**+**
Filippi, 2003[21]	7	Reminders for prescribing of anti-platelet medications to diabetic primary care patients.	.../300*/15,343	Patients with antiplatelet drug prescriptions.	**+**	...	**..**.
Meigs, 2003[22]	6	Feedback for management of type 2 diabetes in a hospital-based internal medicine clinic.	1/66*/598	Use of HbA_1c _and LDL-C tests; BP measurement; eye and foot exams.	**0**	Patients with HbA_1c _<7%; change in HbA_1c _levels.	**0**
Lobach, 1997[23]	6	Recommendations for screening, monitoring, and management of diabetes in primary care.	1/58*/497	Compliance with diabetes management recommendations (foot, ophthalmologic, and complete physical exams; chronic glycaemia monitoring; urine protein and cholesterol levels; and influenza and pneumococcal vaccinations).	**+**	...	**..**.
Nilasena, 1995[24]	7	Reminders for preventive care activities in diabetic outpatients.	2/35*/164	Compliance with preventive care guidelines.	**0**	...	**..**.
Mazzuca, 1990[25]	7	Reminders generated from the medical record system and placed in patients' clinic records for the management of non-insulin dependent diabetes mellitus in outpatients.	4*/114/279	Adherence to five recommendations for care of non-insulin dependent diabetes (HbA_1c _and fasting blood glucose laboratory orders, start home monitoring of blood glucose, diet clinic referral, and start oral hypoglycaemic therapy).	**0**	...	**..**.
Thomas, 1983[26]	2	Recommendations for test ordering, prescribing, and early diagnosis for ambulatory patients in primary care.	1/.../185*	Diabetic clinic visits.	**0**	Emergency department visits; hospitalizations and time hospitalized; BP and glucose levels; obesity.	**..**.

**Diabetes and Other**							

Derose, 2005[27]	7	Recommendations for prescription of ACE-Is, ARBs, and statins in outpatients with diabetes or atherosclerosis.	.../1089/8,557*	Appropriate prescription of ACE-Is, ARBs, or statins within two weeks after patient visit.	**+**	...	**..**.
Sequist, 2005[28]	6	Reminders, based on evidence-based guidelines, for management of diabetes and coronary artery disease in primary care.	20*/194/6,243	Receipt of recommended care for diabetes (cholesterol, HbA_1c_, and dilated eye exams, and use of ACE-Is or statins) or coronary artery disease (cholesterol exam and use of aspirin, beta-blockers, and statins).	**+**	...	**..**.
Martin, 2004[29]	8	Alerts for management of elderly patients in a health maintenance organization setting.	...*/104/8,504	Disenrollment from Health Management Organization plan; patient satisfaction with health plan.	**+**	General health (SF-36 score); inpatient and skilled nursing facility admissions.	**0**
Demakis, 2000[30]	7	Reminders for screening, monitoring, and counselling in accordance with predefined standards of care in ambulatory care.	12*/275/12,989	Compliance with 13 standards of care for coronary artery disease, hypertension, diabetes, smoking cessation, vaccination, warfarin treatment monitoring, atrial fibrillation, myocardial infarction, and gastrointestinal bleeding.	**+**	...	**..**.
Hetlevik, 1999[31-33]	8	Physician-initiated guideline-based guidance for diagnosis and management of hypertension, diabetes mellitus, and hypercholesterolemia in primary care.	56*/56/3,273	Hypertension and diabetic patients without recorded data for BP, serum cholesterol, BMI, smoking status, CHD risk score, and CV inheritance; diabetic patients without recorded data for HbA_1c _levels.	**0**	SBP and DBP levels; serum cholesterol levels; BMI; change in smoking status; change in CHD risk score and proportion of patients with CV inheritance; and, for diabetic patients, HbA_1c _levels.	**0**

**Hypertension**							

Bosworth, 2009[34]	9	Recommendations for management of hypertension in primary care.	1*/32/588	...	**..**.	Change in BP control.	**0**
Hicks, 2008[35]	7	Reminders for management of hypertension in adults in primary care.	14*/.../2,027	Visit-specific adherence to guideline medication prescribing.	**+**	Patients with controlled BP.	**0**
Borbolla, 2007[36]	7	Recommendations for monitoring of BP in outpatients and primary care patients with chronic disease.	.../182*/2,315	BP measurement for appropriate patients.	**+**	Mean SBP and DBP.	**0**
Mitchell, 2004[37]	7	Feedback for identification, treatment, and control of hypertension in elderly patients in primary care.	52*/.../30,345	Patients without BP measurements.	**0**	SBP levels; patients with controlled hypertension.	**0**
Murray, 2004[38]	5	Treatment recommendations for management of hypertension in primary care.	4/...*/712	Compliance with antihypertensive drug recommendations; patient satisfaction with physicians and pharmacists.	**0**	Quality of life measured using SF-36 and a locally validated generic quality of life indicator.	**0**
Montgomery, 2000[39]	10	Computer support system provided patient-specific five-year CV risk for management of hypertension in primary care.	27*/85/614	Number of patients prescribed CV drugs.	**0**	Five-year CV risk; SBP; DBP.	**0**
Rossi, 1997[40]	9	Reminders to modify drug therapy in hypertensive outpatients receiving calcium channel blockers.	1/71/719*	Prescription changes from a calcium channel blocker to another antihypertensive agent.	**+**		**..**.
McAlister, 1986[41]	7	Feedback to physicians for management of hypertension in primary care.	50/50*/2,231	Length of follow up; number of office visits; patients treated for hypertension.	**0**	Patients with DBP ≤90 mmHg; duration of DBP ≤90 mmHg; change in DBP.	**0**
Rogers, 1984[42-44]	4	Detection of deficiencies in care and recommendations for the management of hypertension, obesity and renal disease in outpatients.	1/.../484*	Patients with hypertension given renal function, potassium, or fundoscopic exams, or intravenous pyelograms; number of diets given to or reviewed with obesity patients; patients with renal disease given renal function exams, urine analysis, or urine culture; perceived quality of communication.	**+**	Perceived health status.	**+**
Coe, 1977[45]	4	Recommendations for management of hypertension medication in patients attending hypertension clinics.	2/.../116*	...	**..**.	Adequate BP control.	**0**

**Asthma and COPD**							

Fiks, 2009[46]	8	Alerts for influenza vaccination for children and adolescents with asthma in primary care.	20*/.../11,919	Captured opportunities for vaccination; up-to-date vaccination rates (adjusted analysis).	**0**	...	**..**.
Poels, 2009[47]	10	Presentation of data to assist in the diagnosis and management of chronic airway diseases in primary care.	44*/.../868	Change in diagnoses.	**0**	...	**..**.
Martens, 2007[48,49]	9	Recommendations for appropriate use of antibiotics and management of asthma, COPD, and dyslipidemia.	23*/53/3,496	Appropriate prescribing or lack of prescribing of drugs.	**0**	...	**..**.
Kattan, 2006[50]	8	Recommendations for management of drug therapy in severe asthma in paediatric outpatients.	.../435/937*	Time to appropriate medication step-up; % of scheduled visits within 2 months of medication step-up recommendation.	**+**	Symptom days every 2 weeks.	**0**
Kuilboer, 2006[51]	10	Recommendations for monitoring and treatment of asthma and COPD in primary care.	32*/40/156,772	Contact frequency; peak flow and FEV1 measurements; number of prescriptions for respiratory drugs.	**0**	...	**..**.
Plaza, 2005[52]	9	Guideline-based recommendations to general practitioners and pneumologists for cost-effective management of asthma in primary care.	.../20*/198	Health resource use (spirometry, blood tests, total immunoglobulin E, skin allergy tests, thorax radiography, and oral glucocorticoid prescriptions); medical visits; home visits; visits to other physicians.	**0**	St. George Respiratory Questionnaire total score.	**+**
Tierney, 2005[53]	9	Recommendations for the management of asthma and COPD in adults in primary care.	4/266*/706	Adherence to management recommendations.	**0**	SF-36 subscale scores; McMaster Asthma Quality of Life Questionnaire scores; McMaster Chronic Respiratory Disease Questionnaire scores; emergency department visits; hospitalizations.	**0**
Eccles, 2002[54,55]^b^	10	Care recommendations for management of asthma and angina in adults in primary care.	62*/.../4,506	Adherence to guideline recommendations for angina (record BP, 12-lead and exercise electrocardiogram, Hb and lipid levels, blood glucose levels, thyroid function, and record or provide advice for exercise, weight, and smoking) and medications prescribed for angina; adherence to guideline recommendations for asthma (assessment of lung function, compliance, inhaler technique, and smoking status, and provision of asthma education, action plan, smoking cessation advice, or nicotine replacement therapy) and prescription of drugs for asthma.	**0**	Quality of life (SF-36 and EQ-5D questionnaires); disease-specific quality of life (Seattle angina questionnaire, Newcastle asthma symptoms questionnaire, and the asthma quality of life questionnaire); angina or asthma consultations.	**0**
McCowan2001[56]	8	Guideline-based recommendations for management of asthma in primary care.	...*/46/477	Practice initiated reviews; peak flow meters issued; self-management plans used; symptom assessments; prescriptions for oral corticosteroids and emergency nebulizations.	**0**	Acute asthma exacerbations; patient-initiated primary care consultations.	**+**

**Dyslipidemia**							

Bertoni, 2009[57,58]	9	Recommendations for guideline-consistent screening and treatment of dyslipidemia in primary care.	59*/.../3,821	Change from baseline in number of patients with appropriate lipid management (based on LDL-C and risk strata).	**+**	...	**..**.
Gilutz, 2009[59]	7	Reminders for monitoring and treatment of patients previously hospitalized with coronary artery disease and followed up in primary care.	112*/600/7,448	Appropriate initiation, up-titration, or continuation of statin therapy; rate of adequate lipoprotein monitoring.	**+**	Reduction in LDL-C.	**+**
Lester, 2006[60,61]	8	Recommendations, based on evidence-based guidelines, for the management of patients at high risk for hyperlipidemia in primary care.	1/14/235*	Patients with changes in statin prescriptions at 1 month and 12 months.	**+**	Change in LDL-C.	**0**
Cobos, 2005[62]	10	Recommendations for hypercholesterolemia therapy, follow-up visit frequency, and laboratory test ordering for patients with hypercholesterolemia in primary care.	42*/.../2,221	Number of scheduled physician visits and patient assessments (lipids, aspartate or alanine aminotransferase, or creatine kinase); number of patients treated with lipid-lowering drugs.	**0**	Patients successfully managed according to CV risk level assessed by LDL-C levels or maintenance of CV risk level.	**0**

**Cardiac Care**							

Goud, 2009[63,64]	8	Recommendations for guideline-consistent care plans for outpatient cardiac rehabilitation.	35*/50/2,787	Compliance with guideline recommendations for exercise training, education therapy, relaxation therapy, and lifestyle change therapy.	**+**	...	**..**.
Feldman, 2005[65,66]	9	Recommendations for nurse-coordinated management of patients with heart failure receiving home care.	.../354*/628	Patient adherence to self-management indicators (taking and recognizing medications, salting food, and weighing behavior), home-care related visits, and outpatient doctor visits.	**0**	Kansas City Cardiomyopathy Questionnaire and EuroQoL EQ-5D scale scores; depression (Geriatric Depression Scale); service use (hospitalizations, inpatient nights, and emergency department visits).	**0**
Tierney, 2003[67]	10	Guideline-based recommendations for management of heart disease in primary care.	4*/115/706	Adherence with cardiac care recommendations.	**0**	Quality of life (SF-36 scale and chronic heart disease questionnaire).	**0**
Eccles, 2002[54,55]^b^	10	Care recommendations for management of asthma and angina in adults in primary care.	62*/.../4,506	Adherence to guideline recommendations for angina (record BP, 12-lead and exercise electrocardiogram, Hb and lipid levels, blood glucose levels, thyroid function, and record or provide advice for exercise, weight, and smoking) and medications prescribed for angina; adherence to guideline recommendations for asthma (assessment of lung function, compliance, inhaler technique, and smoking status, and provision of asthma education, action plan, smoking cessation advice, or nicotine replacement therapy) and prescription of drugs for asthma.	**0**	Quality of life (SF-36 and EQ-5D questionnaires); disease-specific quality of life (Seattle angina questionnaire, Newcastle asthma symptoms questionnaire, and the asthma quality of life questionnaire); angina or asthma consultations.	**0**

**Other**							

Lee, 2009[68,69]	6	Recommendations for screening, diagnosis and obesity care planning in acute and primary care.	.../29*/1,874	Encounters with obesity-related diagnoses or missing obesity-related diagnoses, and obesity -related diagnoses not screened and entered in CCDSS.	**+**	...	**..**.
Locatelli, 2009[70]	8	Recommendations for management of chronic kidney disease in nephrology units.	53*/.../599	Use of iron therapy or erythropoetic therapy; guideline-adherent treatment.	**..**.	Achievement of hematological targets (Hb, serum ferritin, hypochromic red cell count); mean Hb level.	**0**
Javitt, 2008[71]	6	Patient-specific recommendations for detecting and correcting medical errors in a health maintenance organization setting.	1/1378/49,988*	Resolution rate for identified problems (add a drug, do a test, or stop a drug).	**+**	...	**..**.
Verstappen 2007[72]	6	Management of methotrexate for early rheumatoid arthritis in adult outpatients.	6/.../299*	...	**..**.	Patients in remission for ≥3 months in first two years.	**+**
Downs, 2006[73]	9	Prompts for the investigation and management of dementia in primary care.	35*/.../450	Detection of dementia; compliance with diagnostic guidelines.	**0**	...	**..**.
Feldstein, 2006b[74]	8	Guideline-recommended osteoporosis care for 50-89 year old women in primary care who experience a fracture.	15/159/311*	Measurement of bone mineral density; use of osteoporosis medication.	**+**	Caloric expenditure; regular physical activity; calcium intake.	**0**
McDonald2005[75]	8	Recommendations to home care nurses for cancer pain assessment and guideline-based management.	1/336*/673	Nurse assessment practices (pain, medications, mood, and bowel movement); nurse instruction practices (medication and side effect management, pain management, contacting physicians, and education); cost-effectiveness for reductions in pain and hospitalizations.	**0**	Pain; quality of life (European Organization for Research and Treatment of Cancer questionnaire); symptom management; cost-effectiveness.	**0**
Dexter, 1998[76]	8	Reminders to discuss and complete advanced directives in outpatients.	4*/10/1,042	Rate of advance directive discussions; rate of form completion.	**+**	...	**..**.
Rubenstein1995[77]	7	Computer-generated feedback designed to identify and suggest management for functional deficits in primary care.	1*/73/557	Clinical problems listed at visits; functional status interventions for patients with functional status problems; physician attitudes toward managing functional status.	**..**.	Functional status (basic and intermediate activities of daily living, mental health, social activities, and work performance); specific impairments (physical, psychological, or social function).	**0**
Petrucci, 1991[78]	6	Recommendations for nurse management of urinary incontinence in elderly patients in nursing homes.	...*/50/27	Nurses' knowledge about urinary incontinence care.	**+**	Wet occurrences.	**+**
McDonald1984[79]	6	Reminders for management of outpatients, including cancer screening, vaccinations, and weight reduction counselling.	1*/130/12,467	Rate of clinician response to indications for care actions.	**+**	Hospitalizations; emergency room and clinic visits; and time averaged values for DBP/SBP; weight; serum glucose; serum Hb; serum potassium; blood urea nitrogen.	**0**

Abbreviations: ACE -I, angiotensin converting enzyme inhibitor; ARB, angiotensin receptor blockers; BMI, body mass index; BP, blood pressure; CCDSS, computerized clinical decision support system; CHD, coronary heart disease; COPD, chronic obstructive pulmonary disease; CV, cardiovascular; DBP, diastolic blood pressure; Hb, haemoglobin; LDL, low-density lipoprotein; SBP, systolic blood pressure.

*Unit of allocation.

^a^Outcomes are evaluated for effect as positive (+) or negative (-) for CCDSS, or no effect (0), based on the following hierarchy. An effect is defined as ≥ 50% of relevant outcomes showing a statistically significant difference (2*p *< 0.05):

1. If a single primary outcome is reported, *in which all components are applicable*, this is the only outcome evaluated.

2. If >1 primary outcome is reported, the ≥50% rule applies and only the primary outcomes are evaluated.

3. If no primary outcomes are reported (or only some of the primary outcome components are relevant) but overall analyses are provided, the overall analyses are evaluated as primary outcomes. Subgroup analyses are not considered.

4. If no primary outcomes or overall analyses are reported, or only some components of the primary outcome are relevant for the care area, any reported prespecified outcomes are evaluated.

5. If no clearly prespecified outcomes are reported, any available outcomes are considered.

6. If statistical comparisons are not reported, 'effect' is designated as not evaluated (...).

^b^Study included in two categories.

### Study quality

Additional file 1, Table S1 presents details of our methodological quality assessment. Of the 55 trials, 53% reported adequate concealment of allocation [2,3,13,18,20,27,29,31-34,37,39,40,47-58,60-67,72-75]; 78% showed no differences in baseline characteristics between study groups or adjusted accordingly [2,3,11-13,18-21,23-25,28,29,34,36,38-58,60-67,70-72,74-76,78,79]; 53% allocated entire wards or practices to each study group [11,12,14-18,25,28-35,37,39,46-49,51,54-59,62-64,67,70,73,76-79]; all except one used objective outcomes or blinding of outcome assessments [23]; and 60% achieved a ≥90% follow-up rate for their unit of analysis [11-13,18-24,27,30,35,36,39,40,46,47,50-55,59-62,65-70,73,74,76,77]. The overall quality of trials was good (median methods score, 8; ranging from 2 to 10) and improved with time (median methods score before versus after year 2000, 7 versus 8, 2-tailed Mann-Whitney U = 137; *p *= 0.005), possibly because early trials often failed to conceal allocation or to achieve adequate follow-up.

### CCDSS and study characteristics

Additional file 2, Table S2 describes CCDSS design and implementation characteristics. Denominators vary because not all trials reported on all features considered. Fifty-nine percent (32/54) of CCDSSs were integrated with electronic medical records [2,3,14-18,21-23,25,26,28,29,31-40,42-44,46,48,49,51,53-55,60-64,67,73,74,76,79], and 17% (8/47) were also integrated with computerized physician order entry systems [22,29,36,38,46,48,49,53,60,61]. Fifty-three percent (25/47) automatically obtained data needed to give recommendations from electronic medical records [2,3,18,21-23,25,28,34-36,38,40,46,48,49,51,53-55,60-64,67,73,74,76,79]; 36% (17/47) relied on practitioners to enter the data [2,3,14-17,23,30,39,41,45,48,49,52-58,67-69,72,75]; and 26% (12/47) used research staff for this purpose [18,24,36,41,47,50,59,65,66,72,75,77,79]. Advice was provided at the time of care in 85% of trials (46/54) [2,3,13-18,20-28,30-36,38-40,42-49,51-59,62-70,73-79] most often on a desktop or laptop computer (51%; 26/51) [2,3,14-17,21,22,28,30,34-39,46-49,51,53-56,62-64,67,70,72-74] or by existing non-prescribing staff (22%; 11/51) [18,23-26,30,40,42-44,71,76,79]. Fifty-three percent (29/54) provided advice to other healthcare practitioners in addition to physicians [2,3,11,12,14-18,22,23,25,26,28-36,38,40,45-47,53,57-59,63,64,67,71,73,76,77,79] and 15% (8/55) directly advised patients in addition to practitioners [2,3,11-13,18,19,29,41,74]. Sixty-four percent (25/39) of systems were pilot tested [11-19,22,23,26,28,31-34,36,37,39,46,47,50,51,56,59-61,63,64,67,72,77] and healthcare professionals were trained to use them in 72% (34/47) [2,3,11-17,19-22,25,29-33,35,36,38,39,46-61,63,64,67-69,73,77,78]. Reports rarely described the CCDSS user interface characteristics.

Seventy-three percent of trials (40/55) declared that at least one author was involved in the development of the system [2,3,11-13,18,19,22-26,28,30,34,36,38-51,53-61,63,64,67-70,72,73,76,77,79] and three trials indicated that all authors were independent of development [14-17,31-33,78].

Additional file 3, Table S3 provides further details of the CCDSS intervention, care setting, study funding source, and year of publication. Trials included a total of 7,335 practitioners (median, 72; ranging from 5 to 1,378 [when reported]) caring for 381,562 patients (median, 719; ranging from 27 to 156,772 [when reported]) in 974 clinics (median, 13; ranging from 1 to 112 [when reported]) across 705 distinct sites (median, 4; ranging from 1 to 112 [when reported]). Eight trials did not report their source of funding [21,26,36,40,71-73,75]. Of the remaining 47, 74% (n = 35) were publicly funded, 17% (n = 8) were conducted with only private funds, [14-17,19,27,48,49,52,60-62,70], and 9% (n = 4) were conducted with a combination of private and public funding [20,29,54,55,75]. The earliest trial was published in 1977 [45], but over one-half (62%) were published after our previous search in September 2004 [2,3,11-20,27-29,34-37,46-53,57-66,68-75].

### CCDSS effects

Table 1 summarizes the effects of all systems for improving process of care and patient outcomes and Additional file 4, Table S4 provides further detail regarding systems and individual outcomes selected for evaluation.

Eighty-seven percent (48/55) of trials measured effects on chronic disease management processes [2,3,11,12,14-19,21-33,35-44,46-69,71,73-76,78,79], and 52% (25/48) demonstrated improvement [2,3,11,12,18,19,21,23,27-30,35,36,40,42-44,50,57-61,63,64,68,69,71,73,74,76,78,79]. Sixty-five percent (36/55) measured impact on patient outcomes [2,3,11-20,22,29,31-39,41-45,50,52-56,59-62,65-67,70,72,74,75,77-79] and 31% (11/36) of these demonstrated benefit on measures such as health-related quality of life, rates of hospitalization, unscheduled care visits, and a host of disease-specific clinical outcomes [2,3,13,18-20,42-44,52,56,59,72,78].

### Diabetes

Thirteen trials described systems primarily supporting diabetes care (median quality score, 7; ranging from 2 to 10) [2,3,11-26]. Fifty-five percent (6/11) reported improvements in processes of care including treatment and monitoring [2,3,11,12,18,19,21,23], while 62.5% (5/8) reported improvements in corresponding patient outcomes including blood pressure, HbA_1c_, and low-density lipoprotein (LDL) cholesterol [2,3,13,18-20]. The seven trials published since 2005 appeared to show success more consistently: four of five improved the process of care [2,3,11-13,18-20], and five of seven improved patient outcomes [2,3,13,18-20].

Systems in five diabetes trials targeted patients in addition to practitioners [2,3,11-13,18,19]. Of these, all four trials that measured process effects demonstrated benefit [2,3,11,12,18,19], and four reported improvement in patient outcomes [2,3,13,18,19].

Several recent trials were conducted in primary community clinics whereas most previous trials were conducted in hospitals. For example, in two trials conducted across multiple practices, CCDSSs provided patient-specific reminders during visits and notified at-risk patients of their care targets and upcoming appointments [2,3,11,18]. Both trials demonstrated improvements in composite process measures comprising timely completion of foot and eye exams, and monitoring of blood pressure, HbA_1c_, lipoproteins, and renal function. Both trials also showed improvements in corresponding composite patient outcomes.

### Diabetes and other conditions

CCDSSs in five trials (median score, 7; ranging from 6 to 8) provided recommendations for a host of conditions in conjunction with diabetes, including dyslipidemia, hypertension, obesity, and heart failure [27-33]. Their effects on diabetes outcomes could not be isolated. All five measured process of care, and 80% (4/5) found improvements [27-30]. Only one measured corresponding patient outcomes, but showed no benefit [31-33].

### Hypertension

The 10 trials focusing primarily on hypertension management (median score, 7; ranging from 4 to 10) were older, with 70% (7/10) published before 2005 [34-45].

Eight of 10 trials assessed impact on process of care using measures such as adherence to recommendations for blood pressure control [35-44], patient satisfaction, and number of scheduled care visits, and four demonstrated improvements [35,36,40,42-44].

In contrast to diabetes systems, however, hypertension systems showed little or no patient benefit. Of the nine trials that reported patient outcomes, such as blood pressure and health-related quality of life [34-39,41-45], only one found benefit [42-44]. This multi-component system improved patients' perceived health status by giving suggestions for the management of hypertension, obesity, and renal disease. The trial, however, was of poor quality (methods score 4), and the nature of the intervention prevented isolating effects related to hypertension.

### Dyslipidemia

Four trials evaluated systems that focused primarily on dyslipidemia [57-62]. All were conducted in primary care settings and published after 2005 (median quality score, 8.5; ranging from 7 to 10).

Three trials measured effects on process of care and demonstrated improvements in lipid monitoring and treatment [57-61], but only one of three trials measuring patient outcomes found a benefit [59]. This CCDSS generated patient-specific reminders that were mailed to primary care physicians and nurses; highlighted the patient's risk factors, lipoprotein values, and current medications; and recommended initiation or adjustment of lipid-lowering treatment when appropriate. The trial detected improvements in blood lipid monitoring and treatment management, as well as relative reductions in patients' LDL cholesterol.

### Asthma and chronic obstructive pulmonary disease (COPD)

The nine trials of systems supporting asthma care were of excellent quality (median score, 9; ranging from 8 to 10) and relatively new (7/9 published after September 2004), but the systems were generally ineffective [46-56]. All trials measured effects on process of care (including rates of spirometry, thorax radiography, IgE levels, and allergy testing; medication prescriptions and influenza vaccinations; and use of rescue medications) but only one demonstrated benefit [50].

Two of five trials measuring patient outcomes found an impact [52,56]. One system delivered asthma recommendations in primary care, made prognostic predictions by matching patients to similar known cases, and allowed users to print self-management plans for their patients [56]. The trial demonstrated a reduction in acute asthma exacerbations and patient-initiated primary care visits. Another system delivered guideline recommendations to general practitioners and pneumologists, and proved to be more cost effective at improving quality of life than usual asthma care [52].

Three asthma systems also gave advice for management of COPD [48,49,51,53]. All of these measured process of care but detected no effects. One trial also measured patient outcomes but did not show benefit [53].

### Cardiac care

Systems in four methodologically strong trials (median score, 9.5; ranging from 8 to 10) focused on heart failure [65,66], cardiac rehabilitation [63,64], ischemic heart disease [67], and angina [54,55]. All measured process of care using adherence to guideline recommendations but only one found benefit [63,64]. The CCDSS for cardiac rehabilitation used electronic medical records and needs assessment data to generate recommendations for exercise training, education, lifestyle change, and stress management [63,64]. The trial demonstrated improved guideline adherence, but patient outcomes were not studied. The other three trials measured effects on quality of life as a patient outcome, but none found benefit [54,55,65-67].

### Other care

We did not group the remaining 12 trials due to their diverse primary indications. They focused on urinary incontinence [78], cancer [75], osteoporosis [74], renal disease [70], functional deficits [77], obesity [68,69], dementia [73], rheumatoid arthritis [72], advance directives [76], and various non-specific indications [71,79]. Most trials found improvements in care process but only two demonstrated benefit to patients: one reduced urinary incontinence in nursing home patients [78], and the other improved likelihood of remission in patients with early rheumatoid arthritis through CCDSS-guided management of methotrexate [72].

### Costs and practical process related outcomes

Four trials used cost-effectiveness as an outcome (see Additional file 4, Table S4) [14-17,52,65,66,75]. Only one trial demonstrated improvement to patient outcomes overall, and the CCDSS was also more cost-effective than usual asthma care [52].

Additional file 5, Table S5 summarizes cost-related findings of the 12 trials that statistically compared costs of care between the study groups: six reported no difference with CCDSS compared to usual care [14-17,29,38,52,67,75], four reported savings with the CCDSS [11,12,50,62,71], and two reported that the CCDSS increased some costs [65-67].

In addition to process of care and patient outcomes, we looked for effects on user satisfaction and workflow (see Additional file 5, Table S5). Only seven trials reported a formal effort of assessing user satisfaction: 3 found users satisfied overall [19,28,63,64], one found them unsatisfied [54,55], and the remaining three showed mixed results [2,3,31-33,56]. The authors of five other studies commented that users were satisfied in informal evaluations [13,18,36,59,72].

Two trials made formal attempts to measure systems' impact on user workflow and reported mixed results [23,63,64].

## Discussion

This review was done in partnership with key decision makers to summarize the effectiveness of clinical decision support technology for the management of chronic conditions. We considered studies 'positive' if they showed a statistically significant improvement in at least 50% of relevant outcomes. CCDSSs often improved the process of patient care. When assessed, effects on any patient outcomes were rarely found, but may have been underestimated: 56% of trials reporting these outcomes declared them primary [11-20,22,29,34,35,38,50,52,56,59-62,67,70,72], and the remaining trials may not have been large enough or long enough to detect such outcomes. No study showed convincing evidence of benefit for major patient outcomes.

Nevertheless, results from recent diabetes management trials are encouraging. Several of these systems were deployed in general community practice and those that engaged both patients and providers were consistently effective. These systems may become increasingly popular with the advent of patient-controlled electronic medical records. Systems addressing several conditions, including but not limited to diabetes, generally improved care but only one measured patient outcomes [31-33] (no effect). In dyslipidemia, systems improved lipid monitoring and treatment, but only one reduced blood lipids [59]. The few dyslipidemia trials were recent and may represent a promising area for future research.

Conversely, most trials in hypertension measured patient outcomes and almost never found benefits, and only some showed improvements in the process of care. Asthma and COPD systems mostly failed to show effectiveness, despite being tested in recent, high-quality trials. The small collection of trials in heart failure, ischemic heart disease, cardiac rehabilitation, and angina also rarely show effects, with improvement only in rehabilitation processes. The remaining systems, too diverse to group, often improved care processes but were seldom found to benefit patients.

While systems in diabetes appear to achieve success with respect to patient outcomes more often than systems in asthma and hypertension, we did not pre-specify this comparison and, given the play of chance and many possible confounders, we cannot confidently assert that the pattern is real. It is plausible that the effectiveness of CCDSS recommendations at improving patient outcomes for some indications is limited by the absence of high-quality clinical evidence in that area. Even the most scientifically sound recommendations, however, will fail to improve health outcomes if patients do not adhere to prescribed treatments--a very common problem [80]. Unfortunately, our suggestions regarding the discrepancy remain purely speculative because studies did not explore reasons for failure, and we do not have enough trials to test these hypotheses reliably.

The growing use of CCDSSs and their potential for benefit and harm highlight the importance of evaluating these systems in well-conducted randomized clinical trials. The increase in number and quality of trials is encouraging, but results remain mixed, and few trials investigated the mechanisms behind their findings. Careful description of study and system design in trial reports, as well as assessments of effectiveness and acceptability of system features, would support progress in this area.

CCDSSs may represent a cost-effective way of improving chronic disease outcomes. However, the economic effects of systems are not readily assessed based on available data. The costs of design, local implementation, ongoing maintenance, and user support can be high, and may be further elevated by the unique nature of chronic care. This warrants cost-effectiveness analyses, but only four trials [14-17,52,65,66,75] reported such data and little cost data of any kind are available across studies. If cost savings exist, however, current results suggest that they are modest.

The benefits we can expect from the use of computerized decision support are not clear. Policy makers promoting the use of CCDSS, as well as healthcare administrators and practitioners considering local implementation, should be aware that the evidence of CCDSS effectiveness is limited, especially with respect to the small number and size of studies of patient outcomes. Further, evidence of benefit comes mainly from a few 'trail blazer' institutions with much in-house informatics expertise, evaluating home-grown systems developed over many years. As a result, trials in this review may not represent the effects in less technically endowed settings or from commercially available systems, the capabilities of which have been shown to vary greatly [81].

Our review has some potential limitations. Great heterogeneity in CCDSS design, purpose, and targets for evaluation prevented us from conducting a meta-analysis. Instead, we used a binary measure of effect, where we considered studies 'positive' if they showed a statistically significant improvement in at least 50% of relevant outcomes. Thus, some of the studies we considered to show no effect found improvement on a minority of secondary or non-prespecified outcomes. These findings could be real but could also be due to *post hoc *unplanned analyses and multiple testing. Readers should refer to the Methods section for a more detailed account of our effect assessment.

We were unable to assess the risk of publication bias in this literature. Given that most systems were studied by their own developers, we suspect that publication bias is likely, and even our findings of modest effects may overestimate the true likelihood of seeing benefit from CCDSSs.

Our method of summarizing the evidence by vote counting inflates the risk of Type 2 error [82] and should generally be approached with caution. However, our results remain essentially unchanged from our 2005 review [4] and are comparable to another major review conducted by Kawamoto and colleagues [83], and a recent 'umbrella ' review of high-quality systematic reviews of CCDSSs in hospital settings [84]. Another recent review of reminder systems [5] (a subset of CCDSS) summarized evidence by effect size meta-analysis and qualified the impact of these interventions as falling below the thresholds of clinical importance. Given the similar conclusions of these other systematic reviews and the risk of publication bias in the CCDSS literature, we have little reason to believe that our methods underestimate the benefit from these systems.

Finally, we observed improvements in the quality of trials over time but this trend may have resulted from better reporting in more recent studies.

## Conclusions

CCDSSs can improve chronic disease management processes and, in some cases, patient outcomes. Recent trials in diabetes care show the most promising results. The mechanisms behind systems' success or failure remain understudied. Future trials with clear descriptions of system design, local context, implementation strategy, costs, adverse outcomes, user satisfaction, and impact on user workflow will better inform CCDSS development and decisions about local implementation.

## Competing interests

RBH, PSR, SM, HCG, AXG, RJS, JAM, NMS, LWK, and TN received support through the Canadian Institutes of Health Research Synthesis Grant: Knowledge Translation KRS 91791 for the submitted work. PSR was also supported by an Ontario Graduate Scholarship, a Canadian Institutes of Health Research Strategic Training Fellowship, and a Canadian Institutes of Health Research 'Banting and Best' Master's Scholarship. Additionally, PSR is a co-applicant for a patent concerning computerized decision support for anticoagulation, which was not discussed in this review, and has recently received awards from organizations that may benefit from the notion that information technology improves health care, including COACH (Canadian Organization for Advancement of Computers in Healthcare), the National Institutes of Health Informatics, and Agfa HealthCare Corp. RJS is the owner of Fig.P Software Incorporated, which develops and sells a chronic disease management system that is not a subject of this review. HCG has/had financial relationships with the following organisations in the previous three years: Sanofi Aventis, GlaxoSmithKline, Eli Lilly, Novo Nordisk, Astra Zeneca, BMS, Roche, Bayer, Janssen Ortho, Solvay, BI, Servier. RBH is acquainted with several CCDSS developers and researchers, including authors of papers included in this review.

## Authors' contributions

RBH was responsible for study conception and design; acquisition, analysis, and interpretation of data; drafting and critical revision of the manuscript; obtaining funding; study supervision. He is the guarantor. PSR acquired, analyzed, and interpreted data; drafted and critically revised the manuscript; and conducted statistical analysis. SM acquired data; drafted and critically revised the manuscript. HCG analyzed and interpreted data; and critically revised the manuscript. AXG acquired, analyzed, and interpreted data; and critically revised the manuscript. RJS analyzed and interpreted the data. JAM acquired, analyzed, and interpreted data; and critically revised the manuscript. LWK and TN acquired data and drafted the manuscript. NLW acquired, analyzed, and interpreted data; provided administrative, technical, or material support; and provided study supervision. All authors read and approved the final manuscript.

## Supplementary Material

Additional file 1 **Table S1. Study methods scores for trials of chronic disease management**. Methods scores for the included studies. Click here for file

Additional file 2 **Table S2. CCDSS characteristics for trials of chronic disease management**. CCDSS characteristics of the included studies. Click here for file

Additional file 3 **Table S3. Study characteristics for trials of chronic disease management**. Study characteristics of the included studies. Click here for file

Additional file 4 **Table S4. Results for CCDSS trials of chronic disease management**. Details results of the included studies. Click here for file

Additional file 5 **Table S5. Costs and CCDSS process-related outcomes for trials of chronic disease management**. Cost and CCDSS process-related outcomes for the included studies. Click here for file
