# Supplementary material for: Computerized clinical decision support systems for chronic disease management: A decision-maker-researcher partnership systematic review
Source: Implement Sci. 2011 Aug 3;6:92. doi: 10.1186/1748-5908-6-92 (PMC3170626; doi:10.1186/1748-5908-6-92)
Supplement: Additional file 1 — Table S1. Study methods scores for trials of chronic disease management. Methods scores for the included studies. [file 1748-5908-6-92-S1.DOCX]

**Additional file 1, Table S1. Study methods scores for trials of chronic disease management^a^**

| **Study** | **Allocation concealed^b^** | **Cluster randomization** | **Adjustment for baseline differences** | **Objective outcome** | **Adequate follow-up** | **Total score** |
| --- | --- | --- | --- | --- | --- | --- |
| Coe, 1977[45] | 0 | 0 | 2 | 2 | 0 | 4 |
| Thomas, 1983[26] | 0 | 0 | 0 | 2 | 0 | 2 |
| McDonald, 1984[79] | 0 | 2 | 2 | 2 | 0 | 6 |
| Rogers, 1984[42-44] | 0 | 0 | 2 | 2 | 0 | 4 |
| McAlister, 1986[41] | 1 | 1 | 2 | 2 | 1 | 7 |
| Mazzuca, 1990[25] | 0 | 2 | 2 | 2 | 1 | 7 |
| Petrucci, 1991[78] | 0 | 2 | 2 | 2 | 0 | 6 |
| Nilasena, 1995[24] | 0 | 1 | 2 | 2 | 2 | 7 |
| Rubenstein, 1995[77] | 0 | 2 | 1 | 2 | 2 | 7 |
| Lobach, 1997[23] | 0 | 1 | 2 | 1 | 2 | 6 |
| Rossi, 1997[40] | 2 | 1 | 2 | 2 | 2 | 9 |
| Dexter, 1998[76] | 0 | 2 | 2 | 2 | 2 | 8 |
| Hetlevik, 1999[31-33] | 2 | 2 | 1 | 2 | 1 | 8 |
| Demakis, 2000[30] | 0 | 2 | 1 | 2 | 2 | 7 |
| Montgomery, 2000[39] | 2 | 2 | 2 | 2 | 2 | 10 |
| McCowan, 2001[56] | 2 | 2 | 2 | 2 | 0 | 8 |
| Eccles, 2002[54, 55]^,^ | 2 | 2 | 2 | 2 | 2 | 10 |
| Filippi, 2003[21] | 0 | 1 | 2 | 2 | 2 | 7 |
| Meigs, 2003[22] | 0 | 1 | 1 | 2 | 2 | 6 |
| Tierney, 2003[67] | 2 | 2 | 2 | 2 | 2 | 10 |
| Martin, 2004[29] | 2 | 2 | 2 | 2 | 0 | 8 |
| Mitchell, 2004[37] | 2 | 2 | 1 | 2 | 0 | 7 |
| Murray, 2004[38] | 0 | 1 | 2 | 2 | 0 | 5 |
| Cobos, 2005[62] | 2 | 2 | 2 | 2 | 2 | 10 |
| Derose, 2005[27] | 2 | 0 | 1 | 2 | 2 | 7 |
| Feldman, 2005[65, 66] | 2 | 1 | 2 | 2 | 2 | 9 |
| McDonald, 2005[75] | 2 | 1 | 2 | 2 | 1 | 8 |
| Plaza, 2005[52] | 2 | 1 | 2 | 2 | 2 | 9 |
| Sequist, 2005[28] | 0 | 2 | 2 | 2 | 0 | 6 |
| Tierney, 2005[53] | 2 | 1 | 2 | 2 | 2 | 9 |
| Downs, 2006[73] | 2 | 2 | 1 | 2 | 2 | 9 |
| Feldstein, 2006[74] | 2 | 0 | 2 | 2 | 2 | 8 |
| Kattan, 2006[50] | 2 | 0 | 2 | 2 | 2 | 8 |
| Kuilboer, 2006[51] | 2 | 2 | 2 | 2 | 2 | 10 |
| Lester, 2006[60, 61]^,^ | 2 | 0 | 2 | 2 | 2 | 8 |
| Augstein, 2007[20] | 2 | 0 | 2 | 2 | 2 | 8 |
| Borbolla, 2007[36] | 0 | 1 | 2 | 2 | 2 | 7 |
| Martens, 2007[48, 49]^,^ | 2 | 2 | 2 | 2 | 1 | 9 |
| Verstappen, 2007[72] | 2 | 0 | 2 | 2 | 0 | 6 |
| Christian, 2008[13] | 2 | 0 | 2 | 2 | 2 | 8 |
| Cleveringa, 2008[14-17] | 0 | 2 | 1 | 2 | 1 | 6 |
| Hicks, 2008[35] | 0 | 2 | 1 | 2 | 2 | 7 |
| Javitt, 2008[71] | 1 | 0 | 2 | 2 | 1 | 6 |
| Peterson, 2008[18] | 2 | 2 | 2 | 2 | 2 | 10 |
| Quinn, 2008[19] | 0 | 0 | 2 | 2 | 2 | 6 |
| Bertoni, 2009[57, 58]^,^ | 2 | 2 | 2 | 2 | 1 | 9 |
| Bosworth, 2009[34] | 2 | 2 | 2 | 2 | 1 | 9 |
| Fiks, 2009[46] | 0 | 2 | 2 | 2 | 2 | 8 |
| Gilutz, 2009[59] | 0 | 2 | 1 | 2 | 2 | 7 |
| Goud, 2009[63, 64] | 2 | 2 | 2 | 2 | 0 | 8 |
| Holbrook, 2009[2, 3] | 2 | 0 | 2 | 2 | 1 | 7 |
| Lee, 2009[68, 69] | 0 | 1 | 1 | 2 | 2 | 6 |
| Locatelli, 2009[70] | 0 | 2 | 2 | 2 | 2 | 8 |
| Maclean, 2009[11, 12] | 0 | 2 | 2 | 2 | 2 | 8 |
| Poels, 2009[47] | 2 | 2 | 2 | 2 | 2 | 10 |

^a^Based on five individual items (score 2 = yes, 1 = partly, and 0 = no) and a summed total score (range 0-10). Because this review update included only randomized, controlled trials, the total score differs from that reported in the previous version of this review[4]: the item evaluating study type (randomized, quasi-randomized, or concurrent controls) has been replaced by one that evaluates use of concealed allocation (concealed, unclear, not concealed).

^b^If allocation concealment was not readily apparent from the description provided in the published article, the primary author of the trial confirmed or indicated that allocation was concealed.
