# Supplementary material for: Computerized clinical decision support systems for chronic disease management: A decision-maker-researcher partnership systematic review
Source: Implement Sci. 2011 Aug 3;6:92. doi: 10.1186/1748-5908-6-92 (PMC3170626; doi:10.1186/1748-5908-6-92)
Supplement: Additional file 2 — Table S2. CCDSS characteristics for trials of chronic disease management. CCDSS characteristics of the included studies. [file 1748-5908-6-92-S2.DOCX]

**Additional file 2, Table S2. CCDSS characteristics for trials of chronic disease management^a^**

| **Study** | **Design** | | | Interface description | | | | | **Data entry source** | | | | | | Methods for delivery of recommendations | | | | | | | **CCDSS users** | | | | | | Other characteristics | | | | |
| --- | --- | --- | --- | --- | --- | --- | --- | --- | --- | --- | --- | --- | --- | --- | --- | --- | --- | --- | --- | --- | --- | --- | --- | --- | --- | --- | --- | --- | --- | --- | --- | --- |
|  | **Stand Alone** | **Integrated with EMR** | **Integrated with CPOE** | Graphic user interface | User must type | Drop down menus | Drag and drop | Other interface | **Automated through EMR** | **Project staff** | **Existing staff** | **Practitioner/decision-maker** | **Patient** | **Other data entry** | Desktop/Laptop computer | E-Mail | PDA | Pager | Project staff | Existing non-prescribing staff | Other Methods | **Trainees** | **Physicians** | **Advanced Practice Nurses** | **Physician Assistants** | **Pharmacists** | **Other health professionals** | Pilot tested | Users trained | Feedback at time of care | CCDSS suggested diagnoses/ treatments/procedures | Authors as developers |
| Coe, 1977[45] | **+** | **-** | **-** | ? | ? | ? | ? | ? | **-** | **-** | **+** | **+** | **-** | **-** | ? | ? | ? | ? | ? | ? | ? | **+** | **+** | **-** | **-** | **-** | **-** | - | - | + | + | + |
| Thomas, 1983[26] | **-** | **+** | **-** | ? | ? | ? | ? | ? | **?** | **?** | **?** | **?** | **?** | **?** | - | - | - | - | - | + | - | **+** | **+** | **-** | **-** | **-** | **-** | + | - | + | + | + |
| McDonald, 1984[79] | **-** | **+** | **?** | ? | ? | ? | ? | ? | **+** | **+** | **-** | **-** | **-** | **-** | - | - | - | - | - | + | - | **+** | **+** | **+** | **-** | **-** | **-** | - | - | + | + | + |
| Rogers, 1984[42-44] | **-** | **+** | **-** | ? | ? | ? | ? | ? | **?** | **?** | **?** | **?** | **?** | **?** | - | - | - | - | - | + | - | **-** | **+** | **-** | **-** | **-** | **-** | - | - | + | + | + |
| McAlister, 1986[41] | **+** | **-** | **-** | ? | ? | ? | ? | ? | **-** | **+** | **-** | **+** | **-** | **-** | - | - | - | - | + | - | - | **-** | **+** | **-** | **-** | **-** | **-** | - | - | - | + | + |
| Mazzuca, 1990[25] | **-** | **+** | **?** | ? | ? | ? | ? | ? | **+** | **-** | **-** | **-** | **-** | **-** | - | - | - | - | - | + | - | **+** | **+** | **-** | **-** | **-** | **-** | - | + | + | + | + |
| Petrucci, 1991[78] | **+** | **-** | **-** | ? | ? | ? | ? | ? | **?** | **?** | **?** | **?** | **?** | **?** | ? | ? | ? | ? | ? | ? | ? | **-** | **-** | **-** | **-** | **-** | **+** | - | + | + | + | - |
| Nilasena, 1995[24] | **+** | **-** | **-** | + | - | - | - | ? | **-** | **+** | **-** | **-** | **-** | **-** | - | - | - | - | + | + | - | **-** | **+** | **-** | **-** | **-** | **-** | - | - | + | + | + |
| Rubenstein, 1995[77] | **+** | **-** | **-** | ? | ? | ? | ? | ? | **-** | **+** | **-** | **-** | **-** | **-** | - | - | - | - | + | - | - | **+** | **+** | **-** | **-** | **-** | **-** | + | + | + | + | + |
| Lobach, 1997[23] | **-** | **+** | **-** | + | ? | ? | ? | ? | **+** | **-** | **+** | **+** | **-** | **-** | - | - | - | - | - | + | - | **-** | **+** | **+** | **+** | **-** | **-** | + | - | + | + | + |
| Rossi, 1997[40] | **-** | **+** | **-** | ? | ? | ? | ? | ? | **+** | **-** | **-** | **-** | **-** | **-** | - | - | - | - | - | + | - | **+** | **+** | **+** | **-** | **-** | **-** | - | - | + | + | + |
| Dexter, 1998[76] | **-** | **+** | **-** | ? | ? | ? | ? | ? | **+** | **-** | **+** | **-** | **-** | **-** | - | - | - | - | - | + | - | **+** | **+** | **-** | **-** | **-** | **-** | - | - | + | + | + |
| Hetlevik, 1999[31-33] | **-** | **+** | **-** | ? | ? | ? | ? | ? | **?** | **?** | **?** | **?** | **?** | **?** | ? | ? | ? | ? | ? | ? | ? | **-** | **+** | **-** | **+** | **-** | **-** | + | + | + | + | - |
| Demakis, 2000[30] | **+** | **-** | **-** | - | + | - | - | + | **-** | **-** | **-** | **+** | **-** | **+** | + | - | - | - | - | + | - | **+** | **+** | **-** | **-** | **-** | **-** | - | + | + | + | + |
| Montgomery, 2000[39] | **-** | **+** | **-** | + | + | - | - | - | **-** | **-** | **+** | **+** | **-** | **-** | + | - | - | - | - | - | - | **-** | **+** | **-** | **-** | **-** | **-** | + | + | + | - | + |
| McCowan, 2001[56] | **+** | **-** | **-** | + | + | + | - | ? | **-** | **-** | **-** | **+** | **-** | **-** | + | - | - | - | - | - | - | **-** | **+** | **-** | **-** | **-** | **-** | + | + | + | + | + |
| Eccles, 2002[54, 55] | **-** | **+** | **-** | ? | ? | ? | ? | ? | **+** | **-** | **-** | **+** | **-** | **-** | + | - | - | - | - | - | - | **-** | **+** | **-** | **-** | **-** | **-** | - | + | + | + | + |
| Filippi, 2003[21] | **-** | **+** | **?** | ? | ? | ? | ? | ? | **+** | **-** | **-** | **-** | **-** | **-** | + | - | - | - | - | - | - | **-** | **+** | **-** | **-** | **-** | **-** | ? | + | + | + | ? |
| Meigs, 2003[22] | **-** | **+** | **+** | + | - | ? | ? | ? | **+** | **-** | **-** | **-** | **-** | **-** | + | - | - | - | - | - | - | **+** | **+** | **+** | **-** | **-** | **-** | + | + | + | + | + |
| Tierney, 2003[67] | **-** | **+** | **-** | ? | ? | ? | ? | ? | **+** | **-** | **-** | **+** | **-** | **-** | + | - | - | - | - | - | - | **+** | **+** | **-** | **-** | **+** | **-** | + | + | + | + | + |
| Martin, 2004[29] | **-** | **+** | **+** | ? | ? | ? | ? | ? | **?** | **?** | **?** | **?** | **?** | **?** | - | - | - | - | - | - | + | **+** | **+** | **+** | **-** | **-** | **-** | ? | + | - | + | ? |
| Mitchell, 2004[37] | **-** | **+** | **-** | - | - | - | - | - | **-** | **-** | **-** | **-** | **-** | **+** | + | - | - | - | - | - | - | **-** | **+** | **-** | **-** | **-** | **-** | + | - | - | + | ? |
| Murray, 2004[38] | **-** | **+** | **+** | + | - | ? | ? | + | **+** | **-** | **-** | **-** | **-** | **-** | + | - | - | - | - | - | + | **+** | **+** | **-** | **-** | **+** | **-** | ? | + | + | + | + |
| Cobos, 2005[62] | **-** | **+** | **-** | ? | ? | ? | ? | ? | **+** | **-** | **-** | **-** | **-** | **-** | + | - | - | - | - | - | - | **-** | **+** | **-** | **-** | **-** | **-** | ? | ? | + | + | ? |
| Derose, 2005[27] | **?** | **?** | **?** | ? | ? | ? | ? | ? | **?** | **?** | **?** | **?** | **?** | **?** | - | - | - | - | - | - | + | **-** | **+** | **-** | **-** | **-** | **-** | ? | ? | + | + | ? |
| Feldman, 2005[65, 66] | **+** | **-** | **-** | ? | ? | ? | ? | ? | **-** | **+** | **-** | **-** | **-** | **-** | - | + | - | - | - | - | - | **-** | **-** | **-** | **-** | **-** | **+** | ? | ? | + | + | ? |
| McDonald, 2005[75] | **+** | **-** | **-** | + | - | ? | ? | + | **-** | **+** | **-** | **+** | **-** | **-** | - | + | - | - | - | - | - | **-** | **-** | **-** | **-** | **-** | **+** | - | ? | + | + | ? |
| Plaza, 2005[52] | **+** | **-** | **-** | ? | ? | ? | ? | ? | **-** | **-** | **-** | **+** | **-** | **-** | - | - | + | - | - | - | - | **-** | **+** | **-** | **-** | **-** | **-** | ? | + | + | + | ? |
| Sequist, 2005[28] | **-** | **+** | **-** | + | + | + | ? | ? | **+** | **-** | **-** | **-** | **-** | **-** | + | - | - | - | - | - | + | **+** | **+** | **-** | **-** | **-** | **-** | + | - | + | + | + |
| Tierney, 2005[53] | **-** | **+** | **+** | + | + | ? | ? | + | **+** | **-** | **-** | **+** | **-** | **-** | + | - | - | - | - | - | + | **+** | **+** | **-** | **-** | **+** | **-** | ? | + | + | + | + |
| Downs, 2006[73] | **-** | **+** | **?** | ? | ? | ? | ? | ? | **+** | **-** | **-** | **-** | **-** | **-** | + | - | - | - | - | - | - | **-** | **+** | **+** | **-** | **-** | **-** | ? | + | + | + | + |
| Feldstein, 2006[74] | **-** | **+** | **?** | + | - | ? | ? | - | **+** | **-** | **-** | **-** | **-** | **-** | + | + | - | - | - | - | - | **-** | **+** | **-** | **-** | **-** | **-** | ? | ? | + | + | ? |
| Kattan, 2006[50] | **+** | **-** | **-** | - | - | - | - | + | **-** | **+** | **-** | **-** | **-** | **-** | - | - | - | - | - | - | + | **-** | **+** | **-** | **-** | **-** | **-** | + | + | ~ | + | + |
| Kuilboer, 2006[51] | **-** | **+** | **-** | ? | ? | + | ? | + | **+** | **-** | **-** | **-** | **-** | **-** | + | - | - | - | - | - | - | **-** | **+** | **-** | **-** | **-** | **-** | + | + | + | + | + |
| Lester, 2006[60, 61] | **-** | **+** | **+** | + | - | - | - | + | **+** | **-** | **-** | **-** | **-** | **-** | - | + | - | - | - | - | - | **-** | **+** | **-** | **-** | **-** | **-** | + | + | - | + | + |
| Augstein, 2007[20] | **+** | **-** | **-** | ? | ? | ? | ? | ? | **?** | **?** | **?** | **?** | **?** | **?** | ? | ? | ? | ? | ? | ? | ? | **-** | **+** | **-** | **-** | **-** | **-** | ? | + | + | + | ? |
| Borbolla, 2007[36] | **-** | **+** | **+** | ? | + | ? | ? | ? | **+** | **+** | **+** | **-** | **-** | **-** | + | - | - | - | - | - | - | **-** | **+** | **-** | **+** | **-** | **-** | + | + | + | + | + |
| Martens, 2007[48, 49] | **-** | **+** | **+** | + | + | ? | ? | + | **+** | **-** | **-** | **+** | **-** | **-** | + | - | - | - | - | - | - | **-** | **+** | **-** | **-** | **-** | **-** | ? | + | + | + | + |
| Verstappen, 2007[72] | **+** | **-** | **-** | ? | ? | ? | ? | ? | **-** | **+** | **-** | **+** | **-** | **-** | + | - | - | - | - | - | - | **-** | **+** | **-** | **-** | **-** | **-** | + | ? | - | + | + |
| Christian, 2008[13] | **+** | **-** | **-** | ? | ? | ? | ? | ? | **-** | **-** | **-** | **-** | **+** | **-** | - | - | - | - | - | - | + | **-** | **+** | **-** | **-** | **-** | **-** | + | + | + | + | + |
| Cleveringa, 2008[14-17] | **-** | **+** | **?** | + | + | ? | ? | ? | **-** | **-** | **-** | **+** | **-** | **-** | + | - | - | - | - | - | - | **-** | **+** | **-** | **-** | **-** | **+** | + | + | + | + | - |
| Hicks, 2008[35] | **-** | **+** | **?** | ? | ? | ? | ? | ? | **+** | **-** | **-** | **-** | **-** | **-** | + | - | - | - | - | - | + | **-** | **+** | **+** | **-** | **-** | **-** | ? | + | + | + | ? |
| Javitt, 2008[71] | **+** | **-** | **-** | ? | ? | ? | ? | ? | **-** | **-** | **-** | **-** | **-** | **+** | - | - | - | - | + | + | - | **-** | **+** | **+** | **-** | **-** | **-** | ? | ? | - | + | ? |
| Peterson, 2008[18] | **-** | **+** | **-** | ? | ? | ? | ? | ? | **+** | **+** | **-** | **-** | **-** | **+** | - | - | - | - | - | + | + | **-** | **+** | **-** | **+** | **-** | **+** | + | - | + | + | + |
| Quinn, 2008[19] | **+** | **-** | **-** | ? | + | ? | ? | + | **-** | **-** | **-** | **-** | **+** | **+** | - | + | - | - | - | - | - | **-** | **+** | **-** | **-** | **-** | **-** | + | + | - | + | + |
| Bertoni, 2009[57, 58] | **+** | **-** | **-** | + | + | + | - | ? | **-** | **-** | **-** | **+** | **-** | **-** | - | - | + | - | - | - | - | **-** | **+** | **+** | **+** | **-** | **-** | - | + | + | + | + |
| Bosworth, 2009[34] | **-** | **+** | **-** | + | + | ? | ? | + | **+** | **-** | **-** | **-** | **-** | **-** | + | - | - | - | - | - | - | **-** | **+** | **+** | **+** | **-** | **-** | + | - | + | + | + |
| Fiks, 2009[46] | **-** | **+** | **+** | ? | ? | ? | ? | ? | **+** | **-** | **-** | **-** | **-** | **-** | + | - | - | - | - | - | - | **+** | **+** | **+** | **-** | **-** | **-** | + | + | + | + | + |
| Gilutz, 2009[59] | **+** | **-** | **-** | ? | ? | ? | ? | ? | **-** | **+** | **-** | **-** | **-** | **-** | - | - | - | - | - | - | + | **-** | **+** | **-** | **-** | **-** | **+** | + | + | + | + | + |
| Goud, 2009[63, 64] | **-** | **+** | **-** | + | + | ? | ? | + | **+** | **-** | **+** | **-** | **-** | **-** | + | - | - | - | - | - | - | **-** | **+** | **-** | **-** | **-** | **+** | + | + | + | + | + |
| Holbrook, 2009[2, 3] | **-** | **+** | **-** | + | - | ? | ? | ? | **+** | **-** | **-** | **+** | **-** | **-** | + | - | - | - | - | - | - | **-** | **+** | **+** | **-** | **-** | **-** | - | + | + | + | + |
| Lee, 2009[68, 69] | **+** | **-** | **-** | + | + | + | ? | + | **-** | **-** | **-** | **+** | **-** | **-** | - | - | + | - | - | - | - | **-** | **-** | **-** | **-** | **-** | **+** | ? | + | + | + | + |
| Locatelli, 2009[70] | **+** | **-** | **-** | ? | ? | ? | ? | ? | **?** | **?** | **?** | **?** | **?** | **?** | + | - | - | - | - | - | - | **-** | **+** | **-** | **-** | **-** | **-** | ? | ? | + | + | + |
| Maclean, 2009[11, 12] | **+** | **-** | **-** | - | - | - | - | - | **-** | **-** | **-** | **-** | **-** | **+** | - | - | - | - | - | - | + | **-** | **+** | **+** | **+** | **-** | **-** | + | + | - | + | + |
| Poels, 2009[47] | **+** | **-** | **-** | ? | ? | ? | ? | ? | **-** | **+** | **+** | **-** | **-** | **-** | + | - | - | - | - | - | - | **-** | **+** | **-** | **-** | **-** | **+** | + | + | + | + | + |

Abbreviations: CCDSS, computerized clinical decision support system; CPOE, computerized physician order entry system; EMR, electronic medical record; PDA, personal digital assistant.

^a^Symbol key: + = characteristic present; - = characteristic absent; ~ = characteristic sometimes present; ? = unstated or uncertain.
