# Supplementary material for: Computerized clinical decision support systems for chronic disease management: A decision-maker-researcher partnership systematic review
Source: Implement Sci. 2011 Aug 3;6:92. doi: 10.1186/1748-5908-6-92 (PMC3170626; doi:10.1186/1748-5908-6-92)
Supplement: Additional file 3 — Table S3. Study characteristics for trials of chronic disease management. Study characteristics of the included studies. [file 1748-5908-6-92-S3.DOCX]

**Additional file 3, Table S3. Study characteristics for trials of chronic disease management^a^**

| **Study (Country)** | **Methods Score^b^** | **Funding Source** | **Indication** | **No. of Practitioners / Patients** | **Setting^c^ (No. of clinics / sites)** | **CCDSS intervention** | **Comparison** |
| --- | --- | --- | --- | --- | --- | --- | --- |
| **Diabetes** | | | | | | | |
| Holbrook, 2009[2, 3] , Canada | 7 | Public | Tracking of diabetes monitoring in adults in primary care. | 46 / 511 | •Primary care  •Community-based clinic  (18/18) | Intervention involved shared access by primary care providers and patients to a Web-based, color-coded diabetes tracker which interfaced with EMRs and an automated telephone reminder system for patients. The tracker system monitored 13 diabetes risk factors, their respective targets and gave brief, prioritised advice, based on national guidelines and a literature review. | Usual care |
| Maclean, 2009[11, 12], USA | 8 | Public | Management of diabetes in primary care. | 132 / 7412 | •Primary care  •Community-based clinic  (64/64) | The Vermont Diabetes Information System (VDIS) is for internal or family medicine practice providers (physicians, nurse practitioners, and physician assistants) and their patients with diabetes. Providers and patients were faxed and mailed reminders, flow sheets and reports on the management of their diabetes. The system used laboratory results on haemoglobin A1C, cholesterol, creatinine and urine protein and sent reminders when testing was overdue, results were elevated and reported on general status of diabetes. | Usual care |
| Christian, 2008[13], USA | 8 | Public | Setting and review of goals for health lifestyle counselling in obese patients with type 2 diabetes at community-based health centres. | 19 / 273 | •Primary care •Community-based clinic (2/2) | CCDSS provided individualised feedback, based on patient self-reports, to increase motivation and readiness to make lifestyle changes, and identify barriers to change. Physicians received a companion report with patient-specific counselling recommendations. | Health education materials |
| Cleveringa, 2008[14-17], The Netherlands | 6 | Private | Management of type 2 diabetes in primary care. | ... / 3391 | •Primary care (55/55) | The Diabetes Care Protocol (DCP) included a CCDSS that contained a diagnostic and treatment algorithm based on the Dutch type 2 Diabetes guidelines that provided patient-specific treatment advice, a diabetes consultation with a practice nurse, a recall system and feedback every three months regarding the percentage of patients meeting the treatment targets. | Usual care |
| Peterson, 2008[18], USA | 10 | Public | Organization of care for primary care patients with type 2 diabetes. | 238 / 7101 | •Primary care •Community-based clinic (24/1) | CCDSS was embedded in an electronic registry and provided visit reminders, patient-specific physician alerts, a monthly progress review, and proactive support of patients at risk. This was part of a multicomponent intervention directed at patients, physicians, and clinic staff to:  •Target high-risk patients  •Develop Registry  •Set-up Administration for staff changes  •Notify patients of targets & appointments; give practitioners patient-specific reminders at visit.  •Identify site coordinator  •Identify local physician champion  •Audit & feedback monthly  •Track outcomes and activity  •Educate staff | Data collection same as for intervention. Sites received baseline data on process and outcome measures and continued usual quality improvement practices. |
| Quinn, 2008[19], USA | 6 | Private | Diabetes management, with remote monitoring of blood glucose, in primary care patients with type 2 diabetes. | 26 / 30 | •Subspecialty clinic •Primary care •Community-based clinic (3/...) | WellDoc System (WDS) is a cell phone-based diabetes management software system that incorporates real-time patient coaching based on blood glucose (BG) measures taken with a bluetooth-adapted One Touch Ultra™ BG meter. The WDS also provided feedback for practitioners, including patient BG logbooks with automated analysis and suggested medication changes. Patients were provided with cell phones and adapted BG meters. | Usual provider care patients were also given One Touch Ultra™ BG meters (LifeScan, Milpitas, CA) and asked to fax or call in their BG logbooks to their providers for review. |
| Augstein, 2007[20], Germany | 8 | Public, Private | Management of diabetes in outpatients. | 5 / 49 | •Hospital outpatients •Subspecialty clinic •Primary care •Community-based clinic (5/5) | The Karlsburg Diabetes Management System (KADIS) used patient-specific data to produce a model of each patient’s glucose metabolism and to simulate patient’s therapeutic regime to optimize blood glucose. Practitioners also received continuous glucose monitoring system data. | Use of continuous glucose monitoring system |
| Filippi, 2003[21], Italy | 7 | ... | Prescribing of anti-platelet medications to diabetic primary care patients with ≥1 additional cardiovascular risk factor. | 300 / 15343 | •Primary care (.../...) | CCDSS was integrated into a standard clinical practice management system, and displayed an electronic reminder when GPs opened medical records of diabetic patients ≥ 30 years of age. Physicians could deactivate the reminder. A letter summarizing practice guidelines, including the benefits of anti-platelet drugs in high-risk diabetics, was also sent to practitioners. | Usual care plus the letter summarizing practice guidelines |
| Meigs, 2003[22], USA | 6 | Public, Private | Management of type 2 diabetes in a hospital-based internal medicine clinic. | 66 / 598 | •Primary care (1/1) | Web-based CCDSS (Diabetes Management Application [DMA]) had to be initiated by providers (included physicians and nurses). It displayed patient-specific information, including laboratory data, on a single screen in real time, allowing for decision support at time of patient contact. The CCDSS interactively linked to evidence-based treatment recommendations and other provider and patient care resources. | Usual care |
| Lobach, 1997[23], USA | 6 | Public | Primary care of diabetes mellitus for outpatients, including screening, vaccination, and monitoring of haemoglobin A1c. | 58 / 497 | •Academic centre •Primary care (1/1) | Rule-based CCDSS used routinely collected data from individual patient EMRs to generate 8 personalised care recommendations for diabetes mellitus based on established guidelines. The recommendations were printed on ‘encounter forms’ used by clinicians to record consultation results. The program was invoked upon request for an encounter form. | Usual care |
| Nilasena, 1995[24], USA | 7 | Public | Screening (foot examination, retinal examination, renal tests), CVD prevention, neurological assessment, and glycaemic control in diabetic outpatients. | 35 / 164 | •Academic centre (2/1) | CCDSS generated reminder reports describing diabetes preventive-health status and listing upcoming or past due preventive health activities for patients with diabetes. Clinical alerts were issued for high-risk aspects of patient’s profile. These were placed at the front of patients’ charts. | Generic reports without patient-specific recommendations were generated |
| Mazzuca, 1990[25], USA | 7 | Public | Management of non-insulin dependent diabetes mellitus in outpatients. | 114 / 279 | •Academic centre (4/4) | 3 treatment groups: CCDSS patient-specific reminders + seminar (B); B + seminar-related clinical materials (C); and C + diabetes patient education service (D).  CCDSS reminders were generated from the medical record system and placed in patients' clinic records whenever the computer detected history, physical, laboratory, or pharmacy data indicating that a seminar recommendation should be considered. | A 3.5-hour seminar covering blood sugar regulation in non-insulin dependent diabetes mellitus was offered to all physicians. All participants received a course syllabus, key reprints, and a reference book. |
| Thomas, 1983[26], USA | 2 | ... | Modification of physician actions at control points (diagnostic test ordering, prescribing treatment, early clinical problem recognition) in ambulatory care process in primary care. | ... / 185 | •Academic centre •Primary care (1/1) | CCDSS (Automated Medical Record Audit System [AMRAS]) updated medical records using data entered by research staff, performed audits based on patient data and protocol-based algorithms, and generated recommendations which were printed in patient reports for physicians before each clinic session. Most recommendations related to general medicine and preventive care. | Usual care |
| **Diabetes and Other** | | | | | | | |
| Derose, 2005[27], USA | 7 | Private | Prescription of ACE-Is, angiotensin receptor blockers, and statins in outpatients with diabetes mellitus or atherosclerotic vascular disease who are at risk for cardiovascular events. | 1089 / 8557 | •Hospital outpatients •Subspecialty clinic •Primary care (.../...) | CCDSS generated recommendations for cardiovascular medications (ACE-Is or statins) in patients at high-risk for CVD. A single-page patient summary sheet, including the recommendations, was faxed to physicians on the morning of a patient visit and attached to the patient’s medical chart. | Usual care. Physicians were faxed the patient summary sheet without recommendations. |
| Sequist, 2005[28], USA | 6 | Public | Management of diabetes and coronary artery disease in primary care. | 194 / 6243 | •Academic centre •Hospital outpatients •Primary care •Community-based clinic (20/20) | When clinicians opened patient charts within EMRs, the CCDSS determined whether the patient had received care in accordance with the recommended evidence-based practice guidelines for care of diabetes or coronary artery disease. Appropriate reminders were then displayed on the patient summary screen of the EMR. Physicians could also choose to have the reminders printed.  All physicians received electronic reminders for overdue preventive care services. | Electronic reminders were suppressed but printing of paper reminders was an option.  All physicians received electronic reminders for overdue preventive care services. |
| Martin, 2004[29], USA | 8 | Public, Private | Drug prescribing, disease management (for congestive heart failure, falls, nutrition, depression, and diabetes mellitus), and case management for patients ≥ 65 years of age in a health maintenance organization setting. | 104 / 8504 | •Primary care•  (.../...) | The Senior Life Management (SLM) program created an electronic health care management record, integrating lab test results and data from claims, prescriptions, and patient surveys and phone calls. CCDSS algorithms generated alerts for program staff about changes in patient clinical status and need for case management screening or service intervention. Program staff included a full-time medical director, an administrator, a social worker, a nurse care coordinator, and 2 non-clinical personal service representatives. The nurse care coordinator was responsible for communication with hospitals, home health care, and physicians (including primary care physicians). Based on published guidelines, the CCDSS also identified when any of 30 medications contraindicated for the elderly were prescribed, and faxed the prescribing physician to suggest reconsideration. | Usual care |
| Demakis, 2000[30], USA | 7 | Public | Screening, monitoring, and counselling in accordance with predefined standards of care in ambulatory care. | 275 / 12989 | •Other •Academic centre •Hospital outpatients (12/12) | Residents received CCDSS-generated reminders relating to 13 prespecified standards of care in 2 ways. 1) On entering a patient name into a computer terminal in the examining room, applicable reminders were automatically displayed in bold letters. 2) Applicable reminders were printed on the standard patient health summary which is attached to patient charts at visits. | Control residents only received the standard health summaries without the reminders. |
| Hetlevik, 1999[31-33], Norway | 8 | Public | Diagnosis and management for hypertension, diabetes mellitus, and hypercholesterolemia in primary care. | 56 / 3273 | •Primary care (56/...) | CCDSS provided guidance for diagnosis, history taking, physical exams, tests, and treatment based on Norwegian clinical guidelines for patients with hypertension, diabetes, or hypercholesterolemia in primary care. The CCDSS was external to, but accessible from, the main computerized medical record system and was initiated by the physician at their discretion. | Usual care |
| **Hypertension** | | | | | | | |
| Bosworth, 2009[34], USA | 9 | Public | Management of hypertension at a Veteran’s affair primary care clinic. | 32 / 588 | •Primary care (1/1) | CCDSS used EMR data to produce and display electronic patient-specific blood pressure (BP) treatment recommendations, including recommendations to increase dose or use a preferred drug. Providers were also given quarterly audit and feedback profiling of their entire panel of patients with respect to guideline-recommended BP targets and medication choices (CCDSS). Some CCDSS patients (CCDSS+ behavioural intervention [BI]) were randomized to also receive a nurse-delivered, telephone BI. | The EMR displayed the patient’s most recent BP, their current hypertension drug regimen, and an optional box for logging an updated BP without any advisories or recommendations for medication management. Some patients in the control group also received the nurse-delivered, telephone, BI. |
| Hicks, 2008[35], USA | 7 | Public | Management of hypertension in a racially diverse group of adult patients in primary care. | ... / 2027 | •Academic centre •Hospital inpatients •Hospital outpatients •Primary care •Community-based clinic (14/...) | CCDSS generated reminders of hypertension treatment recommendations and displayed them to clinicians at patient visits as part of main EMR screen. Paper version of reminders could be printed. 1 of the 7 clinics in the CCDSS group was also randomized to receive additional visits from a nurse practitioner. | Usual care. Same reminders were triggered but were not delivered to clinicians. 1 of the 7 clinics in the usual care group was also randomized to receive additional visits from a nurse-practitioner. |
| Borbolla, 2007[36], Argentina | 7 | ... | Surveillance and monitoring of blood pressure in outpatients and primary care patients with chronic disease (including hypertension, diabetes, CVD, and lipid disorders). | 182 / 2315 | •Academic centre •Hospital outpatients •Primary care (.../25) | CCDSS uses information from both EHRs and Appointment Scheduling Software to detect patients without blood pressure registries (condition I) or with high blood pressure measurements (condition II) and generate reminder lists for receptionists. Receptionists sent identified patients to assistants who assessed blood pressure, weight, height, and risk factors, reminded patients to measure blood pressure weekly and follow treatment directions, and provided educational material. All data was entered in EHRs before physician appointments. | Usual care |
| Mitchell, 2004[37], Scotland | 7 | Public | Identification, treatment, and control of hypertension in elderly patients in primary care. | … / 30345 | •Primary care (52/52) | Audit only (A) practices received “rule of halves” feedback on patients 65 to 79 years of age, including numbers of patients with blood pressure recorded, receiving antihypertensives, and with additional risk factors. Audit plus Strategic (S) practices received “rule of halves” feedback plus color-coded, patient-specific list ranked according to absolute risk of death from stroke in next 10 years for patients with a risk of ≥10%. (this is not very clear in article) | Usual care (no feedback) |
| Murray, 2004[38], USA | 5 | Public | Treatment suggestions for patients with uncomplicated hypertension managed in a primary care internal medicine practice. | ... / 712 | •Academic centre •Primary care (4/4) | 2x2 factorial trial (physician intervention, pharmacist intervention, intervention for physician and pharmacist, no intervention).  Existing computer workstations were programmed to provide treatment suggestions to physicians and pharmacists based on evidence-based guidelines for hypertension management and data in patient EMRs. Physicians received CCDSS-generated care suggestions on paper medication lists at patient visits and on computer workstations when writing orders. Pharmacists received them electronically and could choose to fill the prescription or discuss suggestions with patients or physicians. On-line and printed treatment suggestions were available for all study groups. | CCDSS generated suggestions for both intervention and control groups but they were not displayed for patients in the control group. |
| Montgomery, 2000[39], UK | 10 | Public | Management of hypertension in primary care. | 85 / 614 | •Primary care (27/1) | CCDSS used patient-specific data to calculate the patient’s 5-year risk of a cardiovascular event (newly diagnosed angina, myocardial infarction, coronary heart disease, stroke, or transient ischemic attack) based on New Zealand guidelines for management of high blood pressure. Cardiovascular risk chart, which provides similar risk information, was also provided. | 2 groups: Cardiovascular risk chart alone or usual care |
| Rossi, 1997[40], USA | 9 | ... | Treatment of hypertension in patients treated with calcium channel blockers in primary care. | 71 / 719 | •Academic centre •Subspecialty clinic (1/1) | CCDSS automatically generated reminders which were placed in patient charts by the clinic pharmacist and attached to the medication refill forms given to primary care providers. The reminder highlighted the prescription and offered alternative drugs and doses to calcium channel blockers. | Usual Care |
| McAlister, 1986[41], Canada | 7 | Public | Management of hypertension in primary care. | 50 / 2231 | •Primary care (50/...) | 25 practices in each group.  Physicians recorded patient-specific data, including information about medications and date of next scheduled visit, on encounter forms after visits with hypertensive patients. Forms were mailed to a central test centre, data entered into a CCDSS, and feedback generated for physicians including a chart of diastolic blood pressure, intra- and inter-practice blood pressure percentile rankings, and treatment suggestions based on the “stepped care” protocol. Appointment reminders were also mailed to patients and if a patient missed the appointment, a reminder letter was sent. | Filled out the same collection forms as the study group and mailed them to the study centre. No feedback/reminder was sent to doctors or patients. |
| Rogers, 1984[42-44], USA | 4 | Public | Management of hypertension, obesity and renal disease in outpatients. | ... / 484 | •Academic centre •Subspecialty clinic (1/1) | CCDSS summarized patient demographics, status, and health records and made suggestions based on deficiencies in patient’s care. The 8-page patient medical summary (Northwestern University Computerised Medical Record Summary System, [NUCRSS]) was available to the physician at each visit. | Usual care |
| Coe, 1977[45], USA | 4 | Public | Treatment of hypertension in patients attending hypertension clinics. | ... / 116 | •Academic centre •Subspecialty clinic (2/2) | CCDSS created a compact sequential record of all visits, including a graphic display of blood pressure and drugs in use and provided physicians with hypertension treatment recommendations based on an adaptive algorithm. Physicians were free to follow or reject these recommendations. | Usual care |
| **Asthma and COPD** | | | | | | | |
| Fiks, 2009[46],USA | 8 | Public | Influenza vaccination for children and adolescents with asthma in primary care. | ... / 11919 | •Primary care (20/...) | EHR-based alerts were generated for influenza vaccination in children 5-19 years of age, based on recommendations of the Advisory Committee on Immunization Practices. Bolded and highlighted alerts appeared at the top of the computer screen when an EHR encounter form was opened for an eligible patient, along with a link for ordering vaccine. An influenza education session, with information on the alert system, was provided by 2 expert primary care paediatricians. | Routine care and an influenza education session (without information on the alert system) provided by 2 expert primary care paediatricians |
| Poels, 2009[47], The Netherlands | 10 | Public | Diagnosis and management of chronic airway diseases in primary care. | ... / 868 | •Primary care (44/44) | CCDSS (SpidaXpert®) used algorithms based on patient data, including FEV1, to present pre-and post-bronchodilator values of FEV1 and FEV1/FVC with 95% CIs. This was presented to practitioners graphically and with a textual interpretation. | The Chest Physician Support Group practitioners faxed a print-out of the spirometric test from standard spirometry software (Spida5®) to a chest-physician who responded via standard forms. The Usual Care Group used standard spirometry software (Spida5®) as usual with no additional support. |
| Martens, 2007[48, 49], The Netherlands | 9 | Private | Reminders to change GP's prescribing behaviour for antibiotics, asthma/COPD, and cholesterol prescriptions. | 53 / 3496 | •Academic centre •Primary care •Solo practice (23/...) | CCDSS generated 1 of 2 types of reminders: a) antibiotic/asthma/COPD prescriptions, or b) cholesterol-lowering drug prescriptions. Reminders were based on evidence-based prescribing guidelines and patient data stored in the GPs medical information system; the system included a computerized prescription module. | Physicians in the antibiotic/asthma/COPD reminder group acted as controls for the cholesterol-lowering drug reminder group and vice versa. |
| Kattan, 2006[50], USA | 8 | Public | 5- to 11-year-old children with moderate to severe asthma receiving health care in hospital and community-based clinics and private practices in inner city urban areas. | 435 / 937 | •Hospital outpatients •Solo practice •Community-based clinic  •Primary care(.../7) | Information was collected from each child’s caretaker using a standardised computer-assisted interview every 2 months. The CCDSS used this information and national guidelines to generate a single-page feedback letter that was mailed directly to the child's primary care physician. The letter included a colour photograph of the child, identifying information, details about medication use, asthma symptoms, and health service use, and a 1-sentence treatment recommendation to step up, step down, or don’t change medications. | Usual care. Data were also collected from child caretakers bimonthly but letters were not sent to physicians. The information from the calls was used to determine what recommendation would have been generated. |
| Kuilboer, 2006[51], The Netherlands | 10 | Public | Monitoring and treatment of asthma and COPD in daily practice in primary care. | 40 / 156772 | •Primary care •Solo practice (32/32) | CCDSS uses data in EHR and clinical guidelines to provide feedback on treatment to physicians for patients with asthma or COPD. | Usual care |
| Plaza, 2005[52], Spain | 9 | Private | Management and cost-effectiveness of asthma management in primary care. | 20 / 198 | •Subspecialty clinic •Primary care (.../5) | CCDSS provided recommendations to general practitioners and pneumologists for asthma treatment based on the Global Initiative for Asthma (GINA) guidelines. GINA based intervention included information about chronic inflammatory illness, technique when using an inhaler, maximum expiratory flow (FEM), FEM self-monitoring techniques and GINA recommendations. | Usual care |
| Tierney, 2005[53], USA | 9 | Public | Management of asthma and COPD in adults in primary care. | 266 / 706 | •Academic centre •Primary care (4/...) | Existing computer workstations were programmed to provide care suggestions to physicians and pharmacists based on evidence-based guidelines for asthma and COPD management and data in patient EMRs. Physicians received CCDSS-generated care suggestions on paper medication lists at patient visits and on computer workstations when writing orders. Pharmacists received them electronically and could choose to do nothing, or discuss suggestions with patients or physicians. They received the same educational material as the control group. | Physicians and pharmacists received a printed summary of asthma and COPD management guidelines and could attend rounds about the guidelines but did not receive care suggestions. |
| Eccles, 2002[54, 55] ^d^, UK | 10 | Public, Private | Management of asthma and angina in adults in primary care. | ... / 4506 | •Primary care (62/...) | CCDSS provided internally-developed evidence-based guidelines and care suggestions to general practitioners and practice nurses for management of adults with asthma or angina in primary care, based on electronic patient records. CCDSS was triggered when EMRs of eligible patients were opened or a relevant morbidity code was entered. | Physicians receiving asthma guidelines did not receive angina guidelines and vice versa. |
| McCowan, 2001[56], UK | 8 | Public | Management of asthma in primary care. | 46 / 477 | •Primary care (.../...) | CCDSS (Asthma Crystal Byte) used current asthma guidelines and data entered during consultation to provide management recommendations and reminders. Patient-specific self-management plans and advice sheets could be printed for patients. Physicians and practice nurses evaluated the CCDSS. | Usual care. Practices informed they would have to report on patient outcomes after 6 months. |
| **Dyslipidaemia** | | | | | | | |
| Bertoni, 2009[57, 58], USA | 9 | Public | Guideline-consistent screening and treatment of dyslipidaemia in primary care. | ... / 3821 | •Primary care (59/59) | CCDSS ran on personal digital assistants (PDAs) given to providers (physicians, physician assistants, and nurse practitioners) in the intervention group. CCDSS generated a 1-page report summarizing patient data, low-density lipoprotein cholesterol (LDL-C) level goals, and treatment recommendations, based on National Cholesterol Education Program Third Adult Treatment Panel (ATP III) guidelines. Providers also received print copies of guidelines, education, and academic detailing. | Comparison group were given automatic blood pressure measurement devices, print copies of guidelines, education, and academic detailing. |
| Gilutz, 2009[59], Israel | 7 | Public | Lipid monitoring and treatment of patients previously hospitalised with coronary artery disease (CAD) and followed up in primary care. | 600 / 7448 | •Primary care •Community-based clinic (112/112) | CCDSS collected data from 3 databases (discharge and diagnosis; laboratory; and pharmacy) and automatically generated reminders for management of dyslipidaemia in patients with coronary artery disease based on National Cholesterol Education Program-III and Israeli guidelines. The patient-specific reminders were mailed to physicians and nurses at primary care clinics. The reminders indicated the patient's risk factors, lipoprotein values, and known medications and recommended lipid lowering drug treatment if appropriate. Physicians and nurses could accept or reject CCDSS recommendations. | Usual care |
| Lester, 2006[60, 61] , USA | 8 | Private | Management of patients at high risk for hyperlipidaemia in primary care. | 14 / 235 | •Primary care (1/1) | CCDSS identified high-risk patients with elevated LDL cholesterol levels (> 100mg/dL 6 to 24 months before study initiation) for cholesterol management and sent a single, customised e-mail to physicians. Via emails, users could review patient information and, with a single click, generate a statin prescription, repeat fasting lipid profile, or decline change in medical management. CCDSS recommendations were based on evidence-based guidelines. Existing EHRs were automatically updated. | Usual care |
| Cobos, 2005[62], Spain | 10 | Private | Management of patients with hypercholesterolemia in primary care. | ... / 2221 | •Primary care (42/44) | CCDSS generated recommendations for hypercholesterolemia therapy, follow-up visit frequency, and laboratory test ordering, based on patient data entered by physicians, including CV risk and LDL cholesterol goals. Recommendations were adapted from the European Society of Cardiology and other societies for Hypercholesterolemia Management’s (ESCHM) guidelines. Physicians could adopt or ignore the recommendations. The intervention included availability of patient education promotions such as tablecloths and refrigerator magnets. | Usual care |
| **Cardiac Care** | | | | | | | |
| Goud, 2009[63, 64], The Netherlands | 8 | Public | Use of guideline-concordant care plans for the outpatient rehabilitation of cardiac patients. | 50 / 2787 | •Hospital outpatients (35/35) | The cardiac rehabilitation decision support system (CARDSS) used electronic patient records, needs assessment data (collected and entered into CARDSS by one of the multidisciplinary team), and guideline information (Netherlands Heart Foundation and Netherlands Society for Cardiology) to automatically formulate therapeutic recommendations for each of 4 treatments: exercise training, education therapy, lifestyle change therapy, and relaxation and stress management training. The team was responsible for final therapeutic decisions. CARDSS also provided information management services. | CARDSS control interface comprised all the information management services, but did not provide therapeutic recommendations. |
| Feldman, 2005[65, 66], USA | 9 | Public | Nurse-coordinated management of patients with heart failure receiving home care in an urban setting. | 354 / 628 | •Other •Long term care (includes nursing home) (.../...) | CCDSS identified eligible patients based on initial assessment data and generated patient-specific e-mails highlighting 6 heart failure clinical recommendations for the patient’s assigned nurse. The recommendations were chosen by an expert panel from heart failure clinical practice guidelines. The CCDSS was provided alone (basic intervention) or with provider prompts (laminated card on medication management and prompter card for physician-nurse communication), patient education material, and follow-up outreach from a clinical nurse specialist (augmented intervention). | Usual care |
| Tierney, 2003[67], USA | 10 | Public | Management of heart disease in primary care. | 115 / 706 | •Academic centre •Primary care •Community-based clinic (4/...) | 3 intervention groups: physician, pharmacist, or both. All physicians used an EMR system with computerized order entry.  Physician intervention: CCDSS generated cardiac care suggestions approved by local cardiologists and general internists and based on EMR data, data entered by physicians after visits, and evidence-based guidelines (Agency for Health Care Policy and Research). Suggestions were printed on the patient encounter form and displayed on physician workstations. Physicians could follow or disregard the suggestions. Pharmacist intervention: CCDSS (Pharmacist Intervention Recording System [PIRS] printed a note (rather than bottle labels) when prescriptions were filled for eligible patients, directed pharmacists to care suggestions in PIRS and provided 3 options for action: fill the prescription as usual, discuss care suggestions with the patient, or contact the physician by telephone or PIRS-facilitated e-mail which would be displayed for the physician at next workstation log in. | Usual care with the same EMR and order entry system but without cardiac care suggestions. Longstanding computer-generated preventive care reminders were presented. |
| Eccles, 2002[54, 55] ^d^, UK | 10 | Public, Private | Management of asthma and angina in adults in primary care. | ... / 4506 | •Primary care (62/...) | CCDSS provided internally-developed evidence-based guidelines and care suggestions to general practitioners and practice nurses for management of adults with asthma or angina in primary care, based on electronic patient records. CCDSS was triggered when EMRs of eligible patients were opened or a relevant morbidity code was entered. | Physicians receiving asthma guidelines did not receive angina guidelines and vice versa. |
| **Other** | | | | | | | |
| Lee, 2009[68, 69] , USA | 6 | Public | Diagnosis of obesity in acute and primary care. | 29 / 1874 | •Academic centre •Hospital inpatients •Hospital outpatients •Primary care •Community-based clinic •Emergency Department (.../1) | Personal digital assistant (PDA) based CCDSS enabled adherence to obesity guidelines (undefined). Registered nurses completing advanced practice nurse training used the clinical log to enter patient data into the system, which generated decision support for screening, diagnosis and obesity care planning. The system also provided information on obesity based guidelines through a context specific link. | PDA with clinical log but no decision support features for diagnosis of obesity. |
| Locatelli, 2009[70], Bulgaria, Croatia, Germany, Italy, Latvia, Poland, Romania, Serbia, Montenegro | 8 | Private | Management of chronic kidney disease (CKD) in nephrology units. | ... / 599 | •Subspecialty clinic •Primary care (53/53) | EMR – embedded CCDSS provided management advice, based on European Best Practices Group (EBPG) guidelines, for patients with CKD at nephrology units. | Usual care, access to published guidelines available to all |
| Javitt, 2008[71], USA | 6 | ... | Detecting and correcting medical errors in a health maintenance organization setting. | 1378 / 49988 | •Hospital inpatients •Hospital outpatients •Primary care •Solo practice (1/...) | CCDSS collected information on patients > 11 years of age from billing records, lab feeds, and pharmacies, created a virtual EMR, and applied decision rules to produce patient-specific care considerations (CCs) if indicated. CCs fell into three categories (stop a drug, do a test, and add a drug) and included 3 severity levels. Each CC included issues of concern, suggested actions, and relevant literature citations. CCDSS-associated physicians reviewed each CC. Those that passed review were forwarded to patient physicians by telephone (level 1 severity) or to HMO nurses (level 2 or 3 severity), who reviewed them and could choose to fax them to patient’s physicians. | Usual care |
| Verstappen, 2007[72], The Netherlands | 6 | ... | Management of methotrexate for early rheumatoid arthritis in adult outpatients. | ... / 299 | •Academic centre •Hospital outpatients •Subspecialty clinic (6/1) | CCDSS used information on swollen joint count, tender joint count, erythrocyte sedimentation rate, and visual analogue scale for general well-being to determine whether criteria of response to treatment was met. Changes to treatment were made based on response to treatment according to algorithm. Patients attended outpatient clinic every 4 weeks. | Usual care |
| Downs, 2006[73], UK | 9 | ... | Investigation and management of dementia in primary care. | ... / 450 | •Primary care •Solo practice (35/35) | CCDSS was built into the EMR software for real-time, real case learning. It produced prompts for the investigation and management of dementia. (group 1). | 1. Electronic tutorial on CD Rom (self directed learning)(group 2); 2. Practice based workshops with a standard curriculum designed by a multidisciplinary expert group (peer reflection about real cases). (group 3); 3. control (usual care) (group 4) |
| Feldstein, 2006[74], USA | 8 | Public | Guideline-recommended osteoporosis care for 50-89 year old women in primary care who experience a fracture. | 159 / 311 | •Primary care (15/1) | Patient-specific advice, based on guidelines for osteoporosis management (ordering a BMD measurement and prescribing osteoporosis medication), was delivered via EMR to primary care physicians. Providers who had not ordered a BMD measurement or medication within 3 months of first reminder received a second reminder. In 1 of 2 intervention arms, patients also received a mailed reminder with educational materials. | Usual care |
| McDonald, 2005[75], USA | 8 | ... | Home care nurses' adherence to cancer pain assessment and management guidelines. | 336 / 673 | •Other •Long term care (includes nursing home) (.../1) | Home Care nurses assessed cancer pain and adhered to management guidelines by either responding to a patient-specific, one-time e-mail reminder highlighting six pain-specific clinical recommendations, or the basic intervention augmented by patient education material including a pocket card providing instruction on pain assessment with a 1-10 visual scale to measure patient pain, a prompter card to help improve nurse-physician communication, a self-care guide to review with patients, as well as clinical nurse specialist outreach. | Usual care |
| Dexter, 1998[76], USA | 8 | Public | Reminders to discuss and complete advanced directives in outpatients. | 10 / 1042 | •Academic centre •Primary care (4/...) | Primary care physicians routinely received computer-generated reminders for patients with scheduled visits. These reminders recommend one or both of two types of advance directives for a total of 3 intervention groups: instruction directive and proxy directive reminders, instruction directive reminders only, and proxy directive reminders only. | Usual care, no reminders |
| Rubenstein, 1995[77], USA | 7 | Public | Computer-generated feedback designed to identify and suggest management for functional deficits in primary care. | 73 / 557 | •Academic centre •Primary care (1/1) | After physicians attended a ½ hour education session, they started to receive CCDSS-generated patient-specific functional status reports, which included bar graphs, summarized functional deficits and assessment findings, and provided problem-specific resource and management suggestions. The reports were attached to the front of each new patient’s medical record. Physicians received a booster education session after 3 months, and patients were mailed post-intervention functional status surveys 6 months after their enrolment. | Usual care |
| Petrucci, 1991[78], USA | 6 | Public | Recommendations for nurse management of urinary incontinence in elderly patients in nursing homes. | 50 / 27 | •Long term care (includes nursing home) (.../...) | CCDSS (Urological Nursing Information System [UNIS]) asked questions and provided recommendations for nurses caring for elderly, incontinent patients in nursing homes. Nurses had UNIS for 10 weeks with user support for either 2 (A) or 10 (B) weeks. Patient information was taken by nurses and recommendations were delivered via computers in nurses stations. | Usual care |
| McDonald, 1984[79], USA | 6 | Public | Cancer screening (stool occult blood, mammogram), counselling (weight reduction), immunization (influenza, pneumococcal) in addition to >1000 physician behaviour rules for outpatients. | 130 / 12467 | •Academic centre •Primary care (1/1) | CCDSS used 1491 physician-developed rules to review data in EMR and produce reminder messages for physicians. Printed reports of reminders were attached to patient charts before visits. | Usual care |

Abbreviations: ACE-I, angiotensin-converting enzyme inhibitors; BMD, bone mineral density; CCDSS, computerized clinical decision support system; COPD, chronic obstructive pulmonary disease; CVD, cardiovascular disease; HER, electronic health record; EMR, electronic medical record; GP, general practitioner; LDL, low-density lipoprotein.

^a^Ellipses (…) indicate item was not assessed.

^b^Based on five individual items (score 2 = yes, 1 = partly, and 0 = no) and a summed total score (range 0-10). Because this review update included only randomized, controlled trials, the total score differs from that reported in the previous version of this review[4]: the item evaluating study type (randomized, quasi-randomized, or concurrent controls) has been replaced by one that evaluates use of concealed allocation (concealed, unclear, not concealed).

^c^Diabetes clinic is an example of a subspecialty clinic.

^d^Study included in 2 categories.
