# Supplementary material for: Computerized clinical decision support systems for chronic disease management: A decision-maker-researcher partnership systematic review
Source: Implement Sci. 2011 Aug 3;6:92. doi: 10.1186/1748-5908-6-92 (PMC3170626; doi:10.1186/1748-5908-6-92)
Supplement: Additional file 4 — Table S4. Results for CCDSS trials of chronic disease management. Details results of the included studies. [file 1748-5908-6-92-S4.DOCX]

**Additional file 4, Table S4. Results for CCDSS trials of chronic disease management^a^**

| **Study Name** | **Process of care outcomes** | **CCDSS vs control data** | **Patient outcomes** | **CCDSS vs control data** | **CCDSS process of care effect^b^** | **CCDSS patient effect^b^** |
| --- | --- | --- | --- | --- | --- | --- |
| **Diabetes** | | | | | | |
| Holbrook, 2009[2, 3] | Median follow-up, 5.9 months  **1. 8-item process composite score (out of maximum 10, higher scores better). (primary);** **mean (SD) before/after intervention; mean difference (95%CI).**  Each individual component is reported in the same way (range -2 to +2).  1a. Glycated Hb, measured semiannually. 1b. BP, measured quarterly. 1c. LDL-C, measured semiannually 1d. Albuminuria, measured semiannually. 1e. BMI, measured quarterly. 1f. Foot surveillance, measured semiannually. 1g. Exercise, measured quarterly. 1h. Smoking, measured quarterly.  **1i. Composite score**  2. ABC (Hb, BP, and LDL-C) composite; mean (SD) before/after intervention; mean difference (95%CI) (secondary).  Not prespecified  3. Patients with improvement for total process composite score; n/N, %, mean % difference.  4. Difference (95% CI) in number of recommended visits to primary care provider. | 1a. 0.60 (0.49)/0.88 (0.33) vs 0.62 (0.49)/0.70 (0.46); 0.19 (0.09 to 0.29) 1b. 1.03 (0.79)/1.52 (0.68) vs 1.12 (0.77)/1.27 (0.74); 0.34 (0.19 to 0.49) 1c. 0.49 (0.50)/0.78 (0.42) vs 0.45 (0.50)/0.56 (0.50); 0.18 (0.07 to 0.28) 1d. 0.29 (0.46)/0.70 (0.46) vs 0.30 (0.46)/0.43 (0.50); 0.27 (0.16 to 0.39) 1e. 0.49 (0.64)/0.75 (0.75) vs 0.45 (0.64)/0.54 (0.69); 0.17 (0.02 to 0.32) 1f. 0.28 (0.45)/0.51 (0.50) vs 0.28 (0.45)/0.36 (0.48); 0.16 (0.06 to 0.25) 1g. 1.00 (0.00)/0.69 (0.46) vs 1.00 (0.00)/0.69 (0.46); –0.01 (–0.09 to 0.07) 1h. 1.00 (0.06)/0.69 (0.46) vs 0.97 (0.17)/0.69 (0.46); –0.03 (–0.12 to 0.06) 1i. 5.19 (2.14)/6.52 (2.30) vs 5.19 (2.16)/5.25 (2.52); 1.27 (0.79 to 1.75), *P*<.001  2. 1.80 (1.10)/2.55 (0.83) vs 1.82 (1.08)/2.08 (1.06); 0.49 (0.27 to 0.70)  3. 156/253, 61.7% vs 110/258, 42.6%; 19.1%, *P*<.001  4. 0.66 (0.37 to 1.02), *P*<.001 | Median follow-up, 5.9 months  **1. Clinical composite score; mean (SD) change from baseline; mean difference (95%CI) for 238 vs 241 patients. (secondary)**  Each individual component is also reported in the same way; mean (SD) before/after intervention; mean difference (95%CI). 1a. SBP, mm Hg, for 178/226 vs 195/213 patients. 1b. DBP, mm Hg, for 178/226 vs 195/213 patients. 1c. LDL-C, mmol/L, for 124/197 vs 115/144 patients.  1d. Glycated Hb for 153/222 vs 159/180 patients  1e. Albuminuria, mg/mol, for 63/171 vs 67/101 patients.  1f. BMI for 101/140 vs 92/108 patients. 1g. Exercise, min/wk, median (IQR), for 170/170 vs 178/178 patients. 1h. Feet, no neuropathy for 70/128 vs 72/91 patients.  1i. Nonsmoker for 252/175 vs 250/179 patients.   2. Mean (SD) change in ABC (Hb, BP, LDL-C) clinical composite score at 6 months; difference (95% CI) for 201 vs 193 patients. (secondary)  **3. Change in quality of life (SF-12 and Diabetes-39 questionnaires) at 6 months (secondary).**  4. Number of variables on target (maximum=8); mean (SD) before/after intervention; mean difference (95%CI) for 253/252 vs 258/248 patients (not prespecified).  4a. SBP on target for 178/226 vs 195/213 patients.  4b. DBP on target for 178/226 vs 195/213 patients.  4c. LDL-C on target for 124/197 vs 115/144 patients 4d. Glycated Hb on target for 153/222 vs 159/180 patients 4e. Albuminuria on target for 63/171 vs 67/101 patients 4f. BMI on target for 101/140 vs 92/108 patients 4g. Exercise on target for 253/170 vs 258/178 patients 4h. Feet, no neuropathy on target for 70/128 vs 72/91 patients 4i. Nonsmoker on target for 252/175 vs 250/179 patients  5.Number of ABC variables on target; mean (SD) before/after intervention; mean difference (95%CI) for 211/241 vs 218/227 patients (not prespecified). | 1. 0.33 (1.64) vs -0.16 (1.48); 0.55 (0.04 to 1.07), *P*=.04 1a. 135.2 (17.6)/130.5 (16.4) vs 134.8 (18.4)/135.1 (18.4); –3.95 (–7.64 to –0.26), *P*=.04 1b. 76.1 (11.1)/73.6 (9.9) vs 74.7 (10.3)/75.4 (10.5); –2.38 (–4.60 to 0.17), *P*=.049 1c. 2.41 (0.65)/2.43 (0.78) vs 2.59 (0.87)/2.54 (0.81); –0.002 (–0.14 to 0.14) 1d. 7.0% (1.4)/6.8% (1.2) vs 7.1% (1.6)/7.3% (1.6); –0.20 (–0.38 to –0.02), *P*=.03 1e. 5.80 (15.0)/6.89 (17.9) vs 5.13 (13.2)/5.95 (15.6); 0.65 (–1.11 to 2.41)  1f. 32.1 (8.2)/31.6 (7.5) vs 31.6 (7.0)/31.9 (7.0); 0.02 (–1.24 to 1.28) 1g. 60.0 (180.0)/127.5 (230.0) vs 90.0 (150.0)/122.5 (240.0); 5.18 (–43.50 to 53.86) 1h. 0.94 (0.23)/0.92 (0.27) vs 0.96 (0.20)/0.90 (0.30); 0.01 (–0.08 to 0.10) 1i. 0.88 (0.33)/ 0.87 (0.33) vs 0.84 (0.37)/0.85 (0.36); –0.02 (–0.09 to 0.04)  2. 0.01 (0.41) vs -0.39 (1.26); 0.34 (0.04 to 0.65), *P*=.03  3. no data shown, NS  4. 2.51 (1.44)/3.33 (1.66) vs 2.34 (1.45)/2.49 (1.56); 0.16 (–0.12 to 0.44), *P*=.26 4a. 0.31 (0.47)/0.45 (0.50) vs 0.34 (0.47)/0.34 (0.48);  4b. 0.53 (0.50)/0.69 (0.47) vs 0.57 (0.50)/0.56 (0.50); 0.13 (0.02 to 0.25) for both systolic and DBP on target 4c. 0.66 (0.48)/0.61 (0.49) vs 0.57 (0.50)/0.60 (0.49); –0.02 (–0.14 to 0.10) 4d. 0.56 (0.50)/0.63 (0.48) vs 0.57 (0.50)/0.51 (0.50); 0.08 (–0.01 to 0.17) 4e. 0.83 (0.38)/0.71 (0.45) vs 0.64 (0.48)/0.69 (0.46); –0.01 (–0.11 to 0.09) 4f. 0.30 (0.46)/0.26 (0.44) vs 0.28 (0.45)/0.23 (0.42); –0.001(–0.11 to 0.11)  4g. 0.22 (0.42)/0.36 (0.48) vs 0.18 (0.39)/0.32 (0.47); –0.01 (–0.10 to 0.08)  4h. 0.94 (0.23)/0.92 (0.27) vs 0.96 (0.20)/0.90 (0.30); 0.01 (–0.08 to 0.10) 4i. 0.88 (0.33)/0.87 (0.33) vs 0.84 (0.37)/0.85 (0.36); –0.02 (–0.09 to 0.04)  5. 0.99 (0.81)/1.44 (0.86) vs 0.96 (0.88)/1.02 (0.92); 0.19 (0.004 to 0.38), *P* =.049 | + | + |
| Maclean, 2009[11, 12] | Mean of 32 months follow-up: **1. Proportion of tests that were timely according to guidelines (%); adjusted OR* (95% CI), (secondary). 1a. A1C (testing within 6 months if A1C<7% and 3 months otherwise).  1b. Lipids (yearly if LDL-C<100 mg/dl; 6 months if LDL-C 100-129 mg/dl; and 3 months otherwise). 1c. Serum creatinine (yearly). 1d. Urine microalbumin (yearly unless previous testing was abnormal).**  *Adjusted for baseline patient value, baseline practice performance, and clustering within practices.  Subgroup of patients completed follow-up surveys within 6 months of study completion (not prespecified):  2. Patient’s recall of healthcare utilization in past year (n=704); Mean?**; adjusted effect*** (95% CI) 2a. Primary care visits/year 2b. Specialty visits/year  **Unclear if reported value represents mean.  ***Adjusted for age, sex, marital status, education, health literacy, race, insulin use, comorbidity, hospital clustering within practices. | 1a. 56% vs 55%; 1.17 (0.80 to 1.72), *P*=.43 1b. 74% vs 71%; 1.39 (1.08 to 1.80), *P*=.01 1c. 84% vs 80%; 1.40 (1.06 to 1.84), *P*=.02 1d. 40% vs 32%; 1.74 (1.13 to 2.69), *P*=.01  2a. 2.04 vs 2.86, -0.81 (-1.42 to -0.20), *P*=.01 2b. 0.15 vs 0.23, -0.08 (-0.15 to -0.002), *P*=.04 | Mean of 32 months follow-up: Non-imputed data, n=4998 for A1C (Missing lab results 32% vs 34%, *P=*.09); n=5,450 for LDL-C (Missing lab results 20% vs 23%, *P<*.001). Imputed data, n=7412.   **1. Mean A1C (%); adjusted absolute difference* (95% CI) (primary). 1a. non-imputed data** 1b. imputed data  **2. Proportion of patients with A1C <7% (%); adjusted OR* (95% CI) (primary). 2a. non-imputed data** 2b. imputed data  3. Mean LDL-C (mg/dL); adjusted absolute difference* (95% CI) (secondary). 3a. non-imputed data 3b. imputed data  4. Proportion of patients with LDL-C <100 mg/dL (%); adjusted OR* (95% CI) (secondary). 4a. non-imputed data 4b. imputed data  5. Number, %, deaths (not prespecified).   Subgroup of patients completed follow-up surveys w/thin 6 months of study completion (not prespecified):  6. Physical status (n=672); Mean?**, adjusted effect*** (95% CI) 6a. Systolic BP(mmHg)  6b. Diastolic BP(mmHg)  6c. Body mass index (kg/m²)  7. Functional status (n=688) (Range 0-100); Mean?**, adjusted effect*** (95% CI) 7a. SF-12 Physical  7b. SF-12 Mental  8. Self-care activity (n=564) (Range 0-100); Mean?**, adjusted effect*** (95% CI) 8a. General diet  8b. Specific diet 8c. Exercise 8d. Blood testing 8e. Foot care   9. Audit of Diabetes Dependant Quality of Life (n=658) (range -9 to +9, lower scores = lower quality of life); Mean?;** adjusted effect*** (95% CI)  10. Patient’s recall of healthcare utilization in past year (n=704); Mean?**; adjusted effect**** (95% CI) 10a. Hospital days/year 10b. Emergency room visits/year  *Adjusted for baseline patient value, baseline practice performance, and clustering within practices. **Unclear if reported value represents mean.  ***Adjusted for baseline patient value, age, sex, marital status, education, health literacy, race, insulin use, comorbidity and clustering within practices. ****Adjusted for age, sex, marital status, education, health literacy, race, insulin use, comorbidity, hospital clustering within practices. | 1a. 7.16% vs 7.01%, +0.12 (-0.01 to +0.25), *P*=.08  1b. 7.25% vs 7.10%, +0.10 (-0.05 to +0.24), *P*=.17  2a. 54% vs 59%, 0.84 (0.66 to 1.08), *P*=.18 2b. 54% vs 59%, 0.84 (0.66 to 1.08), *P*=.18  3a. 93.5 vs 93.4, +0.4 (-2.2 to +3.1), *P*=.74 3b. 95.0 vs 95.8, +0.2 (-2.5 to +3.0), *P*=.86   4a. 64% vs 63%, 1.04 (0.87 to 1.23), *P*=.68 4b. 64% vs 63%, 1.04 (0.88 to 1.23), *P*=.65  5. 301/3886 (7.7%) vs 222/3526 (6.3%), *P*=..27  6a. 137.4 vs 138.4, -1.7 (-4.0 to +0.6), *P*=.14 6b. 76.3 vs 76.4, 0.0 (-1.2 to +1.3), *P*=.94 6c. 33.7 vs 33.7, -0.1 (-0.5 to +0.03), *P*=.52  7a. 40.8 vs 40.6, +0.2 (-0.9 to +1.3), *P*=.68 7b. 50.7 vs 50.5, -0.4 (-1.6 to +0.8), *P*=.50  8a. 59.2 vs 61.0, -2.7 (-6.9 to +1.6), *P*=.22 8b. 54.4 vs 51.9, +1.7 (-2.0 to +5.4), *P*=.35 8c. 39.4 vs 33.5, +5.0 (+0.9 to +9.1), *P*=.02 8d. 55.4 vs 63.4, -5.5 (-11.7 to +0.6), *P*=.08 8e. 48.8 vs 52.9, -2.5 (-7.0 to +2.0), *P*=.28  9. -1.2 vs -1.4, +0.12 (-0.04 to +0.28), *P*=.13  10a. 1.18 vs 1.89, -1.01 (-2.02 to -0.01), *P*=.047 10b. 0.55 vs 0.72, -0.23 (-0.42 to -0.04), *P*=.02  Note: there is a question out to the author as to whether or not these numbers are means and whether a higher number in the ranges is better. | + | 0 |
| Christian, 2008[13] | ... | … | **Primary 1. Mean (SD) weight change at 12 months. 2. Proportion (number) of patients with ≥5% weight loss at 12 months.** Secondary 3. Mean (SD) change in physical activity (metabolic-equivalent task minutes/wk) at 12 months. 4. Mean (SD) reduction in calorie intake (kcal/wk) over 12 months. 5. Mean (SD) change in total cholesterol (mg/dL) at 12 months. 6. Mean (SD) change in HDL-C (mg/dL) at 12 months.  7. Mean (SD) change in LDL-C (mg/dL) at 12 months. 8. Mean (SD) change in triglycerides (mg/dL) at 12 months. 9. Mean (SD) change in HbA1c levels at 12 months. Not specified 10. Change (SD) in mean SBP (mm Hg) at 12 months. 11. Change (SD) in mean DBP (mm Hg) at 12 months. 12. Change (SD) in waist circumference (cm) at 12 months. 13. Proportion with ≥6 lbs loss at 12 months. 14. Proportion with weight change +/- 5.9 lbs at 12 months  15. Proportion with ≥6 lbs gain at 12 months. 16. Proportion of patients with HbA1c ≤6.0 at 12 months. 17. Number of patients who had adverse events. | 1. -0.18 (10.92) vs 1.39 (10.60), *P*=.23 2. 21% (30/141) vs 11% (14/132), *P*=.02 3. 354 (574) vs 51 (443), *P*<.001 4. 947 (1936) vs 507 (1963), *P*=.07 5. -15.84 (44.76) vs -3.93 (45.15), *P*=.03 6. -0.43 (17.10) vs 1.56 (11.60), *P*=.26 7. -14.62 (38.52) vs -3.81 (38.51), *P*=.01 8. -13.60 (97.06) vs -9.48 (95.67), *P*=.72  9. -0.14% (1.76) vs -0.46% (1.63), *P*=.12  10. -2.55 (20.37) vs -4.66 (20.81), *P*=.40 11. -2.60 (13.79) vs -2.54 (11.63), *P*=.97 12. -1.764 (7.045) vs -0.543 (6.498), *P*=.14  13. 32% vs. 19%, *P*=.01 14. 41% vs 48%, *P*=.27 15. 26% vs 33%, *P*=.25 16. 22% vs 17%, *P*=NR  17. 1 vs 2 | … | + |
| Cleveringa, 2008[14-17] | **1. Mean (SD) score on diabetes treatment satisfaction questionnaire: baseline / 1 year CCDSS vs baseline / year Control: Per protocol mean difference (95% CI): ITT mean difference (95% CI).** Secondary outcome in unpublished manuscript accepted for publication at Diabetic Medicine.) | 1. 32.4 (±4.7) / 32.8 (± 4.1) vs 32.2 (± 5.1) / 32.6 (±4.8): 0.116 (-0.51 to 0.75): 0.106 (-0.25 to 0.47) | **1. 1-year difference in mean (SD) A1C (%); baseline / 1-year; difference between groups (95% CI) (primary)**  2. Percentage of patients with A1C ≤7%: baseline / 1-year; OR (95% CI) (secondary)  3. Percentage of patients with SBP ≤140 mmHg: OR(95% CI) (secondary)  4. Percentage of patients with total cholesterol ≤4.5 mmol/l: OR (95% CI) (secondary)  5. Percentage of patients with LDL-C ≤2.5 mmol/l: OR (95% CI) (secondary)  6. Percentage of patients with all treatment targets: OR (95% CI) (secondary)  7. SBP (mmHg); baseline / 1-year; difference between groups (95% CI) (not prespecified)  8. DBP (mmHg); baseline / 1-year; difference between groups (95% CI) (not prespecified)  9 Total cholesterol (mmol/l); baseline / 1-year; difference between groups (95% CI) (not prespecified)  10. HDL-Cl (mmol/l); baseline / 1-year; difference between groups (95% CI) (not prespecified)  11. LDL-C (mmol/l); baseline / 1-year; difference between groups(95% CI) (not prespecified)  12. 10-year UK Prospective Diabetes Study CHD risk estimate (%); baseline / 1-year; difference between groups(95% CI) (secondary)  13. Quality adjusted life-years: difference between CCDSS and control (95% CI) (Not prespecified)  13a. all patients  13b. patients with history of CVD  13c. patients without history of CVD  14. Life-years: difference between CCDSS and control (95% CI) (Not prespecified)  14a. all patients  14b. patients with history of CVD  14c. patients without history of CVD  15. Number of CV events: difference between CCDSS and control (95% CI) (Not prespecified)  15a. all patients  15b. patients with history of CVD  15c. patients without history of CVD  Not prespecified  16. Total costs per quality-adjusted life year gained (Euros): difference between CCDSS and control  16a. all patients  16b. patients with history of CVD  16c. patients without history of CVD  17. Total costs per life-year gained (Euros): difference between CCDSS and control  17a. all patients  17b. patients with history of CVD  17c. patients without history of CVD  (**All data below reported as secondary outcomes in an unpublished manuscript accepted for publication at Diabetic Medicine.)  18. Mean (SD) Health Status Questionnaire score: baseline / 1 year CCDSS vs baseline / year Control: Per protocol mean difference (95% CI): ITT mean difference (95% CI) (*Note: non-inferiority threshold above delta=-2%)  18a. Diabetes Health Profile (DHP) total score  18b. DHP Barriers to activity  18c. DHP Psychological distress  18d. DHP Disinhibited eating  18e. SF-36 Physical functioning  18f. SF-36 Social functioning  18g. SF-36 Role physical  18h. SF-36 Role emotional  18i. SF-36 Mental health  18j. SF-36 Vitality  18k. SF-36 Bodily pain  18l. SF-36 General health  18m. SF-36 Health change  19. Mean (SD) score on Other Health Status Questionnaires: baseline / 1 year CCDSS vs baseline / year Control: Per protocol mean difference (95% CI): ITT mean difference (95% CI)  19a. EuroQol-VAS  19b. EuroQol-5 dimensions scale  20. Mean (SD) score on diabetes empowerment scale-short form: baseline / 1 year CCDSS vs baseline / year Control: Per protocol mean difference (95% CI): ITT mean difference (95% CI) | 1. 7.1 (1.3) / 6.9 (1.1) vs 7.0 (1.1) / 6.9 (1.0); 0.07 (-0.02 to 0.16), *P*=NS  2. 60.8 / 68.0 vs 61.6 / 64.2, 1.4 (1.0-1.8), *P*<.05  3. 41.0 / 53.9 vs 39.5 / 42.2; 1.7 (1.2-2.2), *P*<.05  4. 36.2 / 49.0 vs 38.5 / 45.3; 1.3 (1.0-1.6), *P*<.05  5. 41.1 / 53.5 vs 43.8 / 49.8; 1.3 (1.0-2.8), *P*<.05  6. 10.3 / 18.9 vs 10.9 / 13.4; 1.6 (1.3-2.1), *P*<.05  7. 149 (22) / 143 (20) vs 149 (21) / 147 (20.8); 3.3 (0.5-6.0), *P*<.05  8. 83 (11) / 80 (11) vs 82 (11) / 82 (10.6); 2.2, (1.0-3.5), *P*<.05  9. 5.0 (1.0) / 4.6 (0.9) vs 4.9 (1.1) / 4.8 (1.1); 0.2 (0.1-0.3), *P*<.05  10. 1.36 (0.36) / 1.37 (0.37) vs 1.32 (0.35) / 1.33 (0.36); -0.007 (-0.038 to 0.023), *P*=NS  11. 2.8 (0.92) / 2.5 (0.88) vs 2.8 (0.95) / 2.6 (0.97); 0.15 (0.07 to 0.23), *P*<.05  12. 22.5(16.5) / 20.6 (15.0) vs 21.7 (15.8) / 21.6 (15.6); 1.5 (0.3-2.6), *P*<.05  13a. 0.037 (-0.066 to 0.14)  13b. 0.07 (-0.051 to 0.19)  13c. 0.014 (-0.141 to 0.169)  14a. 0.14 (-0.12 to 0.40)  14b. 0.19 (-0.07 to 0.45)  14c. 0.10 (-0.26 to 0.46)  15a. -0.11 (-0.18 to -0.04)  15b. -0.08 (-0.17 to 0.007)  15c. -0.14 (-0.25 to -0.036)  16a. 38 243  16b. 14 814  16c. 121 285  17a. 10 107  17b. 5457  17c. 16 980  18a. 83.1 (±11.9) / 82.9 (±12.0) vs 83.6 (±11.4) / 84.3 (±11.5): -0.880 (-1.94 to 0.12): -0.439 (-1.01 to 0.08)  18b. 85.7 (±13.7) / 84.7 (±13.7) vs 86.1 (± 13.2) / 86.3 (±13.3): -1.163 (-2.34 to 0.03): -0.676 (-1.30 to -0.03)  18c. 89.6 (±11.1) / 89.0 (±12.4) vs 90.7 (±10.6) / 90.8 (±11.1): -0.634 (-1.72 to 0.43): -0.366 (-0.97 to 0.22)  18d. 71.7 (±20.7) / 71.9 (±21.1) vs 72.4 (± 20.9) / 74.4 (±19.6): -1.832 (-3.64 to -0.07): -0.920 (-1.99 to 0.07)  18e. 72.5 (±25.4) / 71.5 (±25.7) vs 73.6 (±23.3) / 72.0 (±24.0): 0.530 (-1.07 to 2.16): 0.154 (-0.73 to 1.05)  18f. 85.4 (±19.9) / 82.6 (±22.4) vs 85.8 (±19.2) / 84.6 (±19.6): -1.569 (-4.30 to 0.72): -1.031 (-2.52 to 0.25)  18g. 71.8 (±39.8) / 70.5 (±39.4) vs 75.3 (±37.0) / 71.8 (±39.6): 2.258 (-1.61 to 6.31): 0.983 (-1.21 to 3.27)  18h. 80.4 (±36.4) / 81.0 (±35.4) vs 83.4 (±33.9) / 83.8 (±33.9): 0.107 (-3.25 to 4.10): 0.112 (-1.79 to 2.35)  18i. 76.7 (±17.4) / 76.4 (±18.4) vs 77.7 (±16.5) / 77.6 (±16.6): -0.240 (-1.52 to 1.15): -0.152 (-0.86 to 0.61)  18j. 63.3 (± 20.2) / 62.9 (± 20.4) vs 64.8 (±19.7) / 64.8 (±19.8): -0.344 (-2.48 to 1.66): -0.211 (-1.43 to 0.95)  18k. 79.7 (±23.4) / 77.8 (±23.8) vs 81.2 (±21.8) / 77.7 (±24.1): 1.629 (-0.48 to 3.78): 0.636 (-0.57 to 1.85)  18l. 60.4 (±17.9) / 59.8 (±18.5) vs 62.3 (±18.4) / 61.8 (±19.0): -0.136 (-1.71 to 1.46): -0.137 (-0.98 to 0.74)  18m. 50.6 (±18.8) / 52.0 (±19.2) vs 51.9 (±18.2) / 49.8 (±17.5): 3.514 (1.23 to 5.82): 1.913 (0.62 to 3.23)  19a. 76.5 (±15.7) / 76.1 (±15.3) vs 78.2 (±14.0) / 76.5 (±15.1): 1.235 (-0.62 to 2.85): 0.573 (-0.48 to 1.48)  19b. 0.817 (±0.22) / 0.813 (±0.23) vs 0.838 (±0.20) / 0.827(±0.21): 0.007 (-0.01 to 0.03): 0.003 (-0.008 to 0.01)  20. 3.78 (±0.64) / 3.78 (±0.69) vs 3.73 (±0.65) / 3.69 (±0.67): 0.042 (-0.06 to 0.14): 0.019 (-0.03 to 0.07) | 0 | 0 |
| Peterson, 2008[18] | Pre-specified 1. Mean (SEM) change in proportion of patients having foot exams over 12 months.  2. Mean (SEM) change in proportion of patients having eye exams over 12 months.  3. Mean (SEM) change in proportion of patients having renal testing over 12 months. 4. Mean (SEM) change in proportion of patients having BP monitoring over 12 months.  5. Mean (SEM) change in proportion of patients having HbA1c testing over 12 months.  6. Mean (SEM) change in proportion of patients having LDL-C testing over 12 months.  **7. Mean (SEM) improvement in process of care index (PCI) at 12 months.**  PCI = annual BP monitoring; eye and foot exams; renal, HbA1c, and LDL-C testing. | 1. 29.4% (5.6) vs -5.6% (5.4), *P*<.001 2. 27% (2.9) vs 1.2% (2.3), *P*<.001 3. 23.2% (5.0) vs -5.3% (4.6), *P*<.001 4. 1.3% (0.9) vs -2.1% (1.4), *P*=.05 5. 2.8% (0.9) vs -5.3% (1.2), *P*<.001 6. 8.9% (1.3) vs 0.3% (1.6), *P*<.001 7. 1.29 (0.042) vs 0.22 (0.038), *P*<.001 | **1. Proportion of patients with target composite clinical outcome at 12 months. (primary)** Not prespecified 2. Proportion of patients with target HbA1c (<7.0%) at 12 months. 3. Proportion of patients with target SBP (<130 mm Hg) at 12 months.  4. Proportion of patients with target LDL-C (<100 mg/dL) at 12 months.  Composite clinical outcome = SBP <130 mm Hg, HbA1c <7.0%, and LDL-C <100 mg/dL. | 1. 12.6% vs 8.5%, *P*<.001 2. 49% vs 43.8%, *P*<.001 3. 45% vs 40.6%, *P*<.001 4. 43% vs 35.5%, *P*<.001 | + | + |
| Quinn, 2008[19] | **Secondary**  **1. Medications intensified at 3 months (% patients). 2. Medication errors identified at 3 months (% patients).** Not prespecified  3. Physician received patient logbooks at 3 months.  Prespecified  4. Patients reporting provider diabetes management improved at 3 months by receipt of blood sugars. | 1. 84.62% vs 23.08%, *P=*.002 2. 53.38% v 0%, *P=*.002 3. 100% vs 7.69%, *P<*.001  4. 100% vs 37.5%, *P=*.004 | **Primary 1. Mean HbA1c levels; Baseline/follow-up at 3 months; difference.**   Prespecified 2. Diet self-care (mean days/week); Baseline/follow-up at 3 months. 3. Medications self-care (mean days/week); Baseline/follow-up at 3 months.  4. Exercise self-care (mean days/week); Baseline/follow-up at 3 months.  5. Patients reporting improved knowledge of food choices at 3 months.  6. Patients reporting improved confidence about diabetes control at 3 months.  7. New depression diagnosis at 3 months (%patients) | 1. 9.51%/7.48% vs 9.05%/8.37%; 2.03% vs 0.68%, *P<*.04  2. 3.15/5.5 vs 3.15/3.86, *P=*.036 3. 5.92/6.64 vs 6.3/6.75, *P=*.495 4. 2.08/2.92 vs 1.23/1.57, *P=*.657 5. 90.91% vs 50%, *P=*.062  6. 100% vs 75%, *P=*.167 7. 9.09% vs 20%, *P=*.37 | + | + |
| Augstein, 2007[20] | ... | … | N randomized/completed study: 24/22 vs 25/24  **Primary outcomes for 3-month follow-up (A1c subgroup by baseline % not prespecified).**  1. A1c. 1a. Mean ± SD A1c % [before vs after] vs [before vs after]; change ± SD**. 1b. Multiple regression analysis for change in A1c associated with CCDSS: beta coefficient, SE, p-value, R2. 2. Mean Sensor Glucose (MSG) levels (mmol/L), mean change ± SD [before vs after] vs [before vs after]** Secondary outcomes for 3-mo follow-up [before vs after] vs [before vs after] 3. Duration of: 3a. hyperglycaemic excursions (hours/day), mean (IQR). 3b. hypoglycaemic excursions (hours/day), mean (IQR). 4. Bread exchange unit intake (BU), mean ± SD. 5. Daily insulin dose (IU), mean (IQR).  Note: euglycemic range = 4.4 to 8.9 mmol/L | 1a. [7.75 ± 1.21 vs 7.41 ± 1.07] vs [7.18 ± 1.42 vs 7.44 ± 1.50];  -0.34 ± 0.49% vs 0.27 ± 0.67%, *P<*.01 1b. -0.608, 0.175, *P=*.001, 21.5% 2. [8.43 ± 1.33 vs 7.59 ± 1.47] vs [7.75 ± 1.33 vs 8.45 ± 2.46] 3a. [4.6 (1.8 to 8.3) vs 1.0 (0.0 to 3.5]) vs [3.2 (0.4 to 6.0) vs 3.5 (1.0 to 9.0)] 3b. [0.0 (0.0 to 0.0) vs 0.0 (0.0 to 0.0)] vs [0.0 (0.0 to 0.1) vs 0.0 (0.0 to 0.0)] 4. [12.6 ± 3.8 vs 12.6 ± 3.9] vs [11.8 ± 4.4 vs 12.9 ± 5.3] 5. [53 (37 to 77) vs 48 (35 to 72)] vs [50.5 (35 to 66) vs 54 (33 to 71)] | … | + |
| Filippi, 2003[21] | **1. n (%) patients with antiplatelet drug prescription: baseline (12 months pre-study/follow-up (over 7 month study); difference (%); OR (95% CI).** 1a. Patients with 1 cardiac risk factor and without CVD. (N=2,651 vs 2,578) 1b. Patients with ≥ 2 cardiac risk factors and without CVD. (N=1,577 vs 1,440) 1c. Patients with CVD. (N=3,802 vs 3,295) **1d. All patients (primary). (N=8,030 vs 7,313)** | 1a. 358 (13.5%)/736 (27.8%) vs 263 (10.2%)/440 (17.1%); 378 (14.3%) vs 177 (6.9%); 2.38 (1.97 to 2.87)  1b. 224 (14.2%)/508 (32.2%) vs 180 (12.5%)/276 (19.2%); 284 (18.0%) vs 9.6 (6.7%); 3.22 (2.52 to 4.12)  1c. 1,304 (34.3%)/1,768 (46.5%) vs 1,229 (37.3%)/1,526 (46.3%); 464 (12.2%) vs 297 (9.0%); 1.36 (1.16 to 1.59)  1d. 1,886 (23.5%)/3,012 (37.5%) vs 1,672 (22.9%)/2,242 (30.7%); 1,126 (14.0%)* vs 570 (7.8%)*; 1.99 (1.79 to 2.22); * = *P<*.001 for change from baseline. | ... | … | + | … |
| Meigs, 2003[22] | N = 307 vs 291 patients. All secondary **1. Glycaemic control outcomes 1a. Patients with ≥ 1 HbA1c test in the last 12 months; baseline n (%), % change from baseline.  1b. Mean (SE) number of preintervention HbA1c tests/year, change from baseline.**  **2. Cholesterol control outcomes 2a. Patients with ≥ 1 LDL-C test in the last 12 months; baseline n (%), % change from baseline. 2b. Mean (SE) number of preintervention LDL-C tests/year, change from baseline.**  **3. Patients with ≥ 1 BP measurement in the last 12 months; baseline n (%), % change from baseline.  4. Patients with ≥ 1 eye examination by an eye-care professional in the last 12 months; baseline n (%), % change from baseline.  5. Patients with ≥ 1 foot examination in the last 12 months; baseline n (%), % change from baseline.** | 1a. 264 (86.0%), +1.6% vs 256 (88.0%), -1.0%; *P=*.3 1b. 1.7 (0.1), +0.3 vs 1.8 (0.1), -0.04; *P=*.008  2a. 177 (57.7%) ,+7.2% vs 167 (57.4%), +3.4%; *P=*.5 2b. 0.8 (0.1), +0.2 vs 0.9 (0.1), +0.01; *P=*.02  3. 299 (97.4%), +1.0% vs 287 (98.6%), -1.4% *P=*.3  4. 90 (29.3%), +5.5% vs 120 (41.2%), +1.7%; *P=*.5  5. 201 (65.5%), +9.8% vs 231 (82.1%), -0.7%; *P=*.003  Note: proportions, means, and comparison of changes were adjusted for clustering and weighted by number of patients per provider. | N = 307 vs 291 patients. **1. Patients with HbA1c <7%; baseline n (%), % change from baseline (primary). 2. Mean (SE) preintervention HbA1c (% of Hb), change from baseline (primary).**  All others are secondary 3. Patients with LDL-C <130 mg/dL; baseline n (%), % change from baseline. 4. Mean (SE) preintervention LDL-C (mg/dL), change from baseline. 5. Patients with BP <130/85 mmHg; baseline n (%), % change from baseline.  6. Mean (SE) preintervention SBP (mmHg), change from baseline. 7. Mean (SE) preintervention DBP (mmHg), change from baseline.  Not prespecified. 8. Increase in proportion of patients taking lipid-lowering drugs who had LDL-C < 130mg/dL. | 1. 51 ( 21.7%), +1.7% vs 61 (26.6%), -2.8%; *P=*.2 2. 8.4 (0.1), -0.23 vs 8.1 (0.1), +0.14; *P=*.09 3. 62 (54.8%), +20.3% vs 78 (63.5%), +10.5%; *P=*.5  4. 126.7 (3.1), -14.7 vs 122.1 (3.2), -9.4; *P=*.3 5. 76 (25.4%), +1.4% vs 79 (29.6%), -2.2%; *P=*.8 6. 138.1 (1.2), +0.8 vs 136.9 (1.2), -2.2; *P=*.03 7. 78.3 (0.6), -1.8 vs 76.4 (0.6), -0.8; *P=*.8 8. 30% vs 10%, *P=*.008  Note: proportions, means, and comparison of changes were adjusted for clustering and weighted by number of patients per provider. | 0 | 0 |
| Lobach, 1997[23] | **1. Overall compliance with diabetes management recommendations during an encounter (median % compliance; p-value) (Primary)** and for individual recommendations:  1a. Foot examination  1b. Complete physical examination  1c. Chronic glycaemia monitoring  1d. Urine protein determination  1e. Cholesterol level  1f. Ophthalmologic examination  1g. Influenza vaccination  1h. Pneumococcal vaccination   2. Median rate (%) overall clinician adherence to guidelines (prespecified); p-value. | 1. 32.0 vs 15.6 (from abstract); *P*=.01 1a. 55.6 vs 30.0; *P*>.1 1b. 33.3 vs 6.7; *P=*.05 1c. 57.4 vs 52.8; *P*>.1 1d. 73.3 vs 3.9; *P=*.01 1e. 43.7 vs 13.4; *P<*.02 1f. 18.8 vs 3.2; *P*>.1 1g. 29.2 vs 22.7; *P*>.1 1h. 19.8 vs 0.0; *P*>.1  2. 65 vs 40 (from fig. 4); *P*=.01 | ... | … | + | … |
| Nilasena, 1995[24] | **1. Compliance with preventive care guidelines.** | 1. 16.9% vs 16.4% (Not significant) | ... | … | 0 | … |
| Mazzuca, 1990[25] | Pre-specified. 3 treatment groups (B - CCDSS reminder + seminar; C = B + seminar-related clinical materials; D = C + diabetes patient education service) vs control (seminar only).   **1. Adherence to 5 recommendations for care of non-insulin dependent diabetes (11 months follow up): number of physicians/number of eligible patients; mean (SE) for B vs C vs D vs A. 1a. Lab order for glycosylated Hb. 1b. Lab order for fasting blood sugar. 1c. Initiation of home-monitored blood glucose.** **1d. Diet clinic referral. 1e. Initiation of oral hypoglycaemic therapy.** | 1a. 114/1591; 0.24 (0.04) vs 0.37 (0.04) vs 0.25 (0.03) vs 0.21 (0.04); *P<*.05 overall, *P<*.05 C vs B, *P<*.05 D vs C  1b. 47/125; 0.80 (0.08) vs 0.69 (0.10) vs 0.70 (0.11) vs 0.68 (0.10), *P*=NS overall 1c. 114/1454; 0.11 (0.03) vs 0.16 (0.03) vs 0.14 (0.03) vs 0.06 (0.02), *P<*.05 overall but NS for individual comparisons 1d. 111/707; 0.18 (0.03) vs 0.15 (0.03) vs 0.22 (0.04) vs 0.14 (0.03); *P*=NS overall 1e. 99/292; 0.24 (0.07) vs 0.26 (0.06) vs 0.31 (0.07) vs 0.20 (0.06); *P*=NS overall | ... | … | 0 | … |
| Thomas, 1983[26] | **Prespecified 1. Mean (unclear if SD or SE) number of visits to diabetic clinic in 1 year.**  Not clearly prespecified.  2. n/N, % suggestions followed for 58 vs 75 patients over 1 year.  Note: This is a preliminary study report. The full report does not appear to have been published. | 1. 4.6 (1.5) vs 4.8 (2.05), *P*=NS  2. 394/784, 50.25% vs 482/1291, 37.5%, *P<*.001 | **Prespecified 1. Number of emergency department visits.**  **2. n/N, %, of patients hospitalised at 1 year. 3. Number of hospitalizations at 1 year. 4. Total days hospitalised 5. Mean (SD) days hospitalised. 6. Change in BP at 1 year.**  **7. Change in obesity at 1 year.  8. Change in glucose at 1 year.** | 1. Data NR, *P*=NS 2. 12/58, 20.7% vs 20/75, 26.7% 3. 20 vs 41 4. 196 vs 594, *P=*.005 5. 9.8 (11.6) vs. 14.5 (16.7) 6. Data NR, *P*=NS 7. Data NR, *P*=NS  8. Data NR, *P*=NS | 0 | … |
| **Diabetes and Other** | | | | | | |
| Derose, 2005[27] | 1-4 primary outcomes 1. Rate of dispensed prescriptions for ACE-Is or ARBs within 2 weeks after the 1st visit by an eligible patient: n/N (%, 95% CI), p-value.  2. Rate of dispensed prescriptions for statins within 2 weeks after the 1st visit by an eligible patient: n/N (%, 95% CI), p-value.  3. Rate of dispensed prescriptions for either type of medication (ACE-I/ARB or statin) within 2 weeks after the 1st visit by an eligible patient: n/N (%, 95% CI); OR, 95% CI, p-value. (OR>1 = benefit for CCDSS)  **4. OR (95% CI) for prescribing ACE-I, ARB, or statins in intervention vs control group, controlling for number of visits, medication recommended, and patient age, sex, and past medication use.**  Subgroup analyses (not clearly prespecified).  5. OR for intervention vs control specialists/primary care physicians.  6. Interaction for number of visits (1 vs >1) and treatment group (CCDSS vs control).  Note: Included pts were those eligible for ACE-I/ARB but not dispensed drug in past 12 months or eligible for statins or other lipid-lowering drug but not dispensed drug in past 6 months. | 1. 164/2311 (7.1%, 6.1 to 8.2) vs 134/2367 (5.7%, 4.8 to 6.7), *P*=.048 2. 171/2103 (8.1%, 7.6 to 10.2 vs 160/2080 (7.7%, 6.6 to 8.9, *P*= .61 3. NR/4414 (7.6%, 6.8 to 8.4) vs NR/4447 (6.6%, 5.9 to 7.4), *P=*.08 4. 1.192 (1.01 to 1.40), *P=*.04  5. 1.16/1.20, *P=*.92 for interaction. 6. No significant interaction for number of visits and treatment group. | ... | ... | + | … |
| Sequist, 2005[28] | **1. Receipt of recommended care for diabetes using the 5-item composite outcome during the 6 month study, % patients; OR (95% CI). (Primary)  2. Receipt of recommended care for CAD using the 4-item composite outcome during the 6 month study, %; OR (95% CI). (Primary)**  3. Receipt of recommended components of diabetes care during the 6 month study; HR (95% CI): 3a. Annual cholesterol exam.  3b. Biennial HbAlc exam  3c. Annual dilated eye exam  3d. Hypertension/ACE-I use  3e. Statin use for LDL-C ≥ 130 mg/dL   4. Receipt of recommended components of CAD care during the 6 month study; HR (95% CI): 4a. Annual cholesterol exam  4b. Aspirin use 4c. β-blocker use  4d. Statin use for LDL-C ≥130 mg/dL  Not prespecified 5. Mean number of diabetes reminders per patient. 6. Mean number of CAD reminders per patient.  Note: HR>1 = benefit for CCDSS | 1. 19% vs 14%; 1.30 (1.01 to 1.67) 2. 22% vs 17%; 1.25 (1.01 to 1.55)  3a. 1.41 (1.15 to 1.72), *P=*.001 3b. 1.14 (0.89 to 1.46), *P=*.29 3c. 1.38 (0.81 to 2.32), *P=*.23 3d. 1.42 (0.94 to 2.14), *P=*.10 3e. 1.10 (0.65 to 1.85), *P=*.73  4a. 0.99 (0.75 to 1.29), *P=*.92 4b. 2.36 (1.37 to 4.07), *P=*.002 4c. 1.09 (0.72 to 1.63), *P=*.69 4d. 1.51 (1.05 to 2.17), *P=*.03  5. 6.1 vs 6.7, *P=*.004 6. 4.3 vs 5.4, *P<*.001 | ... | ... | + | … |
| Martin, 2004[29] | 1. Disenrollment from plan (secondary).  **2. Self-reported patient satisfaction with health plan (change from baseline to 18 months); score range 0=worst, 10=best) (primary)** | 1. No differences (no data reported).  2. 0.32 vs 0.12; *P<*.01 | **Primary outcomes over 18 months. 1. SF-36 domains (change from baseline to 18 months) 1a. General health.** 1b. Bodily pain. 1c. Mental health. 1d. Physical function. 1e. Role limitation — emotional. 1f. Role limitation — physical. 1g. Social function. 1h. Vitality. 1i. Mental component — summary score. 1j. Physical component — summary score. **2. Inpatient admissions per 1000 per year. 3. Inpatient days per 1000 per year. 4. Skilled nursing facility admissions per 1000 per year.** **5. Skilled nursing facility days per 1000 per year.**  Secondary 6. Number (proportion) of deaths over 18 months. | 1a. -1.50 vs -2.29; *P=*.09 1b. -0.78 vs -1.42; *P=*.35 1c. -0.13 vs 0.01; *P=*.74 1d. -4.29 vs -4.04; *P=*.67 1e. -2.73 vs -2.24; *P=*.66 1f. -3.09 vs -4.45; *P=*.28 1g. -1.42 vs -2.77; *P=*.04 1h. -1.53 vs -2.28; *P=*.14 1i. -0.16 vs -0.23; *P=*.79 1j. -1.25 vs -1.56; *P=*.21  2. 430 vs 421; *P=*.89 3. 1929 vs 1989; *P=*.46 4. 36 vs 37; *P=*.73 5. 616.3 vs 747.7; *P=*.02 6. 191/4257 (4.5%) vs 211/4247 (5.0%), *P=*.18 | + | 0 |
| Demakis, 2000[30] | Primary outcomes **1. Proportion of patients in compliance with all 13 standards of care over 17 months. N, % adherent; OR (95% CI). 1a. All standards.** 1b. Coronary artery disease, lipid levels. 1c. Hypertension: weight, exercise, sodium. 1d. Diabetes: glycosylated hb level. 1e. Diabetes: nutrition counselling. 1f. Diabetes: urinalysis. 1g. Diabetes: eye exam. 1h. Diabetes of peripheral vascular disease: foot exam. 1i. Smokers: cessation counselling. 1j. Age ≥65 or high risk: pneumonoccal vaccination. 1k. Warfarin treatment monitoring. 1l. Atrial fibrillation: warfarin, aspirin, or ticlopidine. 1m. Myocardial infarction: β-blocker. 1n. Gastrointestinal bleeding/NSAID therapy: switch drugs.   **2. Proportion of all visits for which care was indicated and residents provided proper care over 17 months. N, % adherent; OR (95% CI). 2a. All standards.** 2b. Coronary artery disease: lipid levels. 2c. Hypertension: weight, exercise, sodium. 2d. Diabetes: glycosylated Hb level. 2e. Diabetes: nutrition counselling. 2f. Diabetes: urinalysis. 2g. Diabetes: eye exam. 2h. Diabetes or peripheral vascular disease: foot exam. 2i. Smoking cessation counselling. 2j. Age ≥65y or high risk: pneumococcal vaccination. 2k. Warfarin treatment: monitoring.  2l. Atrial fibrillation: warfarin, aspirin, or ticlopidine. 2m. Myocardial infarction: beta-blocker. 2n. Gastrointestinal bleeding/NSAID therapy: switch drugs. | 1a. 19,373, 58.8% vs 20,575, 53.5%; 1.24 (1.08 to 1.42, P = 0.002). 1b. 1813, 79.0% vs 1894, 78.3%; 1.05 (0.82 to 1.34, *P=*.72) 1c. 4244, 55.2% vs 4471, 49.3%; 1.27 (0.92 to 1.75, *P=*.14) 1d.1904, 70.6% vs 2089, 65.9%; 1.24 (0.89 to 1.73, *P=*.19) 1e. 1896, 61.6% vs 2064, 53.3%; 1.29 (0.93 to 1.79, *P=*.12) 1f. 1614, 69.8% vs 1804, 62.6%; 1.38 (1.13 to 1.68, *P=*.001) 1g. 1760, 73.5% vs 1942, 63.4%; 1.60 (1.29 to 2.00, *P<*.001) 1h. 2160, 48.6% vs 2330, 42.8%; 1.26 (1.02 to 1.56, *P=*.03) 1i. 935, 63.5% vs 968, 54.8%; 1.44 (1.01 to 2.05, *P=*.04) 1j. 1759, 12.7% vs 1688, 4.3%; 3.26 (2.09 to 5.09, *P<*.001) 1k. 287, 67.3% vs 276, 64.5%; 1.13 (0.68 to 1.88, *P=*.63) 1l. 236, 75.0% vs 241, 81.7%; 0.67 (0.41 to 1.09), *P=*.10 1m. 275, 44.7% vs 334, 41.3%; 1.15 (0.81 to 1.62, *P=*.42) 1n. 490, 65.5% vs 474, 67.9%; 0.90 (0.65 to 1.23, *P=*.49)   2a. 12,759, 17.9% vs 14,013 12.2%; 1.57 (1.45 to 1.71, *P*<.001) 2b.833, 30.4% vs 815, 24.4%; 1.35 (1.07 to 1.71, *P=*.01) 2c. 3540, 17.0% vs 3896, 10.3%; 1.77 (1.53 to 2.05, *P<*.001) 2d. 1037, 26.5% vs 1184, 20.1%; 1.43 (1.17 to 1.77, *P=*.001) 2e. 1596, 17.0% vs 1800, 13.7%; 1.29 (1.05 to 1.58, *P=*.02) 2f. 972, 20.3% vs 1190, 16.0%; 1.34 (1.06 to 1.68, *P=*.01) 2g. 796, 17.7% vs 1094, 9.0%; 2.19 (1.63 to 2.94, *P<*.001) 2h. 2169, 13.1% vs 2201,5.5%; 2.57 (2.02 to 3.26, *P<*.001) 2i. 471, 12.5% vs 514, 8.2%; 1.61 (1.02 to 2.53, *P=*.04) 2j. 883, 7.9% vs 829, 1.1%; 7.85 (3.83 to 16.08, *P<*.001) 2k. 105, 32.4% vs 122, 42.6%; 0.64 (0.36 to 1.15, *P=*.13) 2l. 62, 54.8% vs 66, 53.0%; 1.08 (0.51 to 2.28, *P=*.85) 2m. 150, 18.0% vs 189, 18.0%; 1.00 (0.54 to 1.85, *P*>0.99) 2n. 145, 24.8% vs 113, 31.0%; 0.74 (0.40 to 1.34, *P=*.31) | ... | … | + | … |
| Hetlevik, 1999[31-33] | Prespecified **1. Proportion of hypertension patients (total N = 2239) without recorded data, difference (95% CI) 1a. BP over last 12 months  1b. Serum cholesterol over last 12 months  1c. BMI over 18 months  1d. Smoking status over 18 months  1e. CHD risk score over 18 months  1f. CV inheritance over 18 months   2. Proportion of diabetic patients (total N = 1034) without recorded data, difference (95% CI). 2a. BP over last 12 months 2b. Serum cholesterol over last 12 months  2c. BMI over 18 mo  2d. Smoking status over 18 months  2e. CHD risk score over 18 months 2f. CV inheritance over 18 months  2g. HbA1c over last 12 months** | 1a. 14.3% vs 14.2%, 0.1 (-3.0 to 3.2) 1b. 62.3% vs 56.8%, 5.5 (1.2 to 9.8) 1c. 81.5% vs 89.2%, -7.7 (-10.8 to -4.6)  1d. 82.9% vs 87.1%, -4.2 (-7.4 to -1.0) 1e. 91.7% vs 91.9%, -0.2 (-2.6 vs 2.2) 1f. 79.5% vs 73.4%, 6.1 (2.4 to 9.8)  2a. 18.7% vs 18.5%, 0.2 (-5.2 to 5.6) 2b. 56.3% vs 62.7%, -6.4 (-13.2 to 0.4) 2c. 78.2% vs 93.0%, -14.8 (-19.5 to -9.9)  2d. 82.6% vs 94.5%, -11.9 (-16.3 to -7.5) 2e. 91.1% vs 98.3%, -7.2 (-10.3 vs -4.1) 2f. 78.7% vs 83.4%, -4.7 (-10.2 to 0.8) 2g. 20.5% vs 18.8%, 1.7 (-3.8 to 7.2) | For hypertension patients at 18 mo (total N = 2239) Prespecified 1. Mean (SD) and change for SBP (mm Hg) in last 12 months (n=1727).  2. Mean (SD) and change for DBP (mm Hg) in last 12 months (n=1727).  3. Mean (SD) and change for serum cholesterol (mmol/L) in last 12 months (n=821).  4. Mean (SD) and change for BMI (kg/m2) in last 18 months (n=286).  5. Proportion and change in proportion of smokers at 18 months (n=297).  6. Mean (SD) and change in CHD risk score at 18 months  6a. Women (n=89).  6b. Men (n=76).   7. Proportion and change in proportion of patients with CV inheritance at 18 months (n=482).   **For hypertension patients at 21 months (after feedback on missing data at 18 months). 8. Mean (SD) and change for SBP (mm Hg) (n=1839).  9. Mean (SD) and change for DBP (mm Hg) (n=1839).  10. Mean (SD) and change for serum cholesterol (mmol/L) (n=1349).  11. Mean (SD) and change for BMI (kg/m2) (n=1053).  12. Proportion and change in proportion of smokers (n=1160).  13. Mean (SD) and change in CHD risk score 13a. Women (n=500).  13b. Men (n=391).  14. Proportion and change in proportion of patients with CV inheritance (n=1235).**   Note: Mean (SD) SBP higher in CCDSS group at baseline. 159.1 (20.3) vs 156.4 (19.7), difference 2.7 (1.0 to 4.5).  Prespecified For diabetic patients at 18 months (total N = 1034) 15. Mean (SD) and change (95% CI) for SBP (mm Hg) in last 12 months (n=648).  16. Mean (SD) and change for DBP (mm Hg) in last 12 months (n=648).  17. Mean (SD) and change for serum cholesterol (mmol/L) in last 12 months (n=321).  18. Mean (SD) and change for BMI (kg/m2) in last 18 months (n=112).  19. Proportion and change in proportion of smokers at 18 months (n=89).  20. Mean (SD) and change in CHD risk score at 18 months  20a. Women (n=19).  20b. Men (n=22).  21. Proportion and change in proportion of patients with CV inheritance at 18 months (n=150).   22. Mean (SD) and change in HbA1c level in last 12 months (n=640).   **For diabetic patients at 21 months (after feedback on missing data at 18 months) 23. Mean (SD) and change (95% CI) for SBP (mm Hg) (n=697).  24. Mean (SD) and change for DBP (mm Hg) (n=697).  25. Mean (SD) and change for serum cholesterol (mmol/L) (n=535).  26. Mean (SD) and change for BMI (kg/m2) (n=427).  27. Proportion and change in proportion of smokers (n=460).  28. Mean (SD) and change in CHD risk score 28a. Women (n=184).  28b. Men (n=142).  29. Proportion and change in proportion of patients with CV inheritance (n=452).  30. Mean (SD) and change in HbA1c level (n=689).**   After 18 months, CCDSS had been used in treatment of 104 hypertension patients (12%) and 52 of diabetic patients (14%). | 1. 156.7 (19.5) vs 155.5 (18.7), 1.2 (-0.6 to 3.0) 2. 88.6 (9.7) vs 89.6 (8.8), -1.0 (-1.9 to -0.2) 3. 6.6 (1.2) vs 6.7 (1.3), -0.1 (-0.3 to 0.1) 4. 28.9 (4.3) vs 28.6 (4.9), 0.3 (-0.9 to 1.3) 5. 23% vs 29%, -6 (-16 to 4) 6a. 18.3 (19.8) vs 25.2 (24.2), -6.9 (-16.3 to 2.5) 6b. 56.0 (42.0) vs 65.1 (83.4), -9.1 (-40.7 to 22.6) 7. 76% vs 89%, -13.0 (-20.1 to 5.9) 8. 156.8 (19.4) vs 155.6 (19.0), 1.2 (-0.6 to 3.0) 9. 88.8 (9.7) vs 89.8 (8.9), -1.0 (-1.9 to -0.2) 10. 6.64 (1.2) vs 6.57 (1.3), 0.07 (-0.1 to 0.2) 11. 27.8 (4.5) vs 27.7 (4.8), 0.1 (-0.4 to 0.7) 12. 21% vs 19%, 2.0 (-2.6 to 6.6) 13a. 17.9 (17.9) vs 20.6 (23.5), -2.7 (-6.3 to 1.0) 13b. 67.9 (83.9) vs 66.8 (73.4), 1.1 (-14.6 to 6.9) 14. 62% vs 66%, -4.0 (-14.5 to 6.5) 15. 151.4 (22.2) vs 153.7 (20.5), -2.3 (-5.6 to 1.0) 16. 82.8 (10.7) vs 85.3 (9.9), -2.4 (-4.0 to -0.9) 17. 6.2 (1.5) vs 6.3 (1.2), -0.1 (-0.3 to 0.2) 18. 29.6 (5.0) vs 29.8 (5.7), -0.2 (-2.4 to 2.0) 19. 23% vs 30%, -7 (-28.3 to 14.3) 20a. 30.2 (32.8) vs 12.5 (9.3), 17.7 (-18.0 to 53.4)  20b. 39.8 (33.9) vs 68.7 (83.4), -28.9 (-229.1 to 171.3) 21. 84% vs 94%, -10.0 (-19.8 to -0.3) 22. 7.9 (1.6) vs 8.0 (1.6), -0.1 (-0.4 to 0.1)  23. 151.5 (22.1) vs 152.7 (19.0), -1.2 (-4.4 to 2.0) 24. 82.8 (10.6) vs 85.1 (10.1), -2.3 (-3.8 to -0.8) 25. 6.2 (1.3) vs 6.2 (1.3), 0 26. 28.6 (5.1) vs 28.3 (6.3), 0.3 (-0.8 to 1.4) 27. 19% vs 16%, 3.0 (-4.0 to 10.0) 28a. 14.3 (17.7) vs 14.2 (17.5), 0.1 (-5.1 to 5.2)  28b. 51.4 (53.5) vs 48.7 (44.1), 2.6 (-14.2 to 19.5) 29. 66% vs 63%, 3.0 (-5.8 to 11.8) 30. 7.8 (1.6) vs 7.9 (1.6), -0.1 (-0.4 to 0.1) | 0 | 0 |
| **Hypertension** | | | | | | |
| Bosworth, 2009[34] | … | … | 1. % (SEM) of patients in BP control over 24-mo: baseline / 24 mo / difference: p value for expected baseline to 24-month change within each group (primary) 1a. CCDSS+nurse-delivered behavioural Intervention (BI) 1b. CCDSS alone 1c. Control+BI 1d. Control alone  2. Change in BP control between groups (intervention groups compared to CTRL alone group) (primary).  3. % (SEM) of patients in systolic BP control over 24-mo: baseline / 24 mo / difference: p value for expected baseline to 24-month change within each group (secondary) 3a. CCDSS+BI 3b. CCDSS alone 3c. Control+BI 3d. Control alone  4. Change in systolic BP control between groups (intervention groups compared to control alone group).  **5. Change in control. CCDSS vs Control 5a. BP (primary)** 5b. systolic BP (secondary) | 1a. 36.2 (4.8) / 48.1 (8.4) / 11.8 (9.8): *P=*.23 1b. 44.9 (5.1) / 43.7 (7.7) / -1.2 (9.1): *P=*.89 1c. 44.2 (5.1) / 59.5 (7.6) / 15.7 (8.9): *P=*.08 1d. 32.0 (4.6) / 43.9 (7.7) / 11.9 (8.8): *P=*.18 1d. 1.8(9.8), 0.23  2. Overall intervention group by time effect *P=*.56  3a. 139.2 (1.4) / 136.8 (1.7) / -2.3 (2.1): *P=*.26 3b. 139.1 (1.4) / 136.9 (1.6) / -2.1 (1.9): *P=*.27 3c. 138.8 (1.4) / 136.3 (1.6) / -2.5 (2.0): *P=*.20 3d. 141.6 (1.4) / 136.8 (1.6) / -4.9 (1.9): *P=*.01  4. Overall intervention group by time effect *P=*.73  5a. *P=*.34  5b. *P=*.46 | … | 0 |
| Hicks, 2008[35] | At 18 months.  **1. Proportion of visits with triggered or suppressed reminders that had adherence to guideline medication prescribing within 1 week; adjusted OR (95% CI). (primary)** | 1. 7% vs 5%; 1.32 (1.09 to 1.61); *P=*.002  No interaction for intervention effect by race/ethnicity. | **At 18 months**  **1. n/N (%) patients with BP controlled; adjusted OR (95% CI). (primary)**  2. Mean BP at 18 months (mm Hg) (prespecified).  2a. SBP.  2b. DBP. | 1. 410/859 (48%) vs 527/1168 (45%); 0.96 (0.78 to 1.19); *P*=NS  Secondary analyses excluding patients without documented BP at index or outcome visit was consistent and analysis by race/ethnicity showed no difference in intervention effects (data not reported).  2. 138 vs 137, *P=*.67 2b. 77 vs 78, *P=*.05  Secondary analysis: no difference in intervention effects by race/ethnicity. | + | 0 |
| Borbolla, 2007[36] | **Primary outcome 1. Proportion of patients (without BP registries) with at least one BP measurement during the three months period, n (%, 95% CI).**  **2. Proportion of patients (with high BP measurements) with at least one BP measurement during the three months period, n (%, 95% CI).** | 1. 207(49.9%, 45 to 55) vs 195 (37%, 33 to 41), *P<*.001  2. 224 (61%, CI NR) vs 239 (50%, CI NR), *P=*.002 | **Secondary outcome 1. Mean SBP and DBP, mm Hg.** | 1. 140/78 vs 138/78, *P=*.162/*P=*.914 | + | 0 |
| Mitchell, 2004[37] | No specific outcomes prespecified All outcomes: (A) pre/post vs (S) pre/post vs (C) pre/post unless otherwise stated.  **1. Mean percentage of patients identified with BP and no record of BP**   **2. Among known hypertensives, mean percentage with no record of BP** | 1. 34.2%/26.3% vs 18.8%/14.2% vs 22.4%/17.9% Between group differences not significant.  2. 19.6%/14.0% vs 3.9%/3.4% vs 10.4%/7.7% Between group differences not significant. | **1. Mean final SBP (adjusted values*); difference, 95% CI 1a. audit (A) vs control (C) 1b. audit plus strategic (S) vs control (C)** 1c. audit (A) vs audit plus strategic (S)  **2 Final proportion of patients with hypertension controlled; RR adjusted for initial hypertension control*, 95% CI. 2a. audit (A) vs control (C) 2b. audit plus strategic (S) vs control (C)**  *Adjusted for gender, smoking, and social deprivation and practice level factors, training | 1a. 152.3 vs 150.8; 1.51, -0.57 to 4.41, *P=*.707 1b. 149.2 vs 150.8; -1.54, -4.06 to.0.49, *P=*.555 1c. 152.3 vs 149.2; 3.05, 1.26 to 5.81, *P=*.026  2a. 35.4% vs 46.5%; 0.93, 0.57 to 1.53, *P=*.770 2b. 49.4% vs 46.5%; 1.72, 1.06 to 2.79, *P=*.028  **For outcome 5 above, adjusted RRs do not appear consistent with the reported data. | 0 | 0 |
| Murray, 2004[38] | All reported as pharmacist vs physician vs pharmacist + physician vs control groups at 12 months (n=180 vs 181 vs 180 vs 171 patients): **1. Compliance with treatment suggestions (secondary): n (%) patients with suggestions; mean (SD) adherence rate. 1a. All antihypertensive drug suggestions.** 1b. Start or increase ACE-I. 1c. Start diuretic. 1d. Start or increase calcium channel blocker. 1e. Start or increase β-blocker.  Not prespecified 2. Total number of antihypertensive drug suggestions/mean (SD) per patient.  **Secondary**  **3. Patient satisfaction with physicians and pharmacists.** | 1a. 117 (65%) vs 123 (68%) vs 125 (69%) vs 114 (67%); 25 (33) vs 29 (36) vs 35 (39) vs 26 (33); *P=*.13 1b. 89 (42%) vs 92 (51%) vs 96 (53%) vs 91 (53%); 33 (47) vs 44 (50) vs 41 (49) vs 30 (46); *P*=NS 1c. 54 (30%) vs 55 (30%) vs 52 (29%) vs 58 (34%); 22 (42) vs 22 (42) vs 25 (44) vs 31 (47); *P*=NS 1d. 38 (21%) vs 56 (31%) vs 46 (26%) vs 51 (30%); 47 (51) vs 34 (48) vs 39 (49) vs 49 (51); *P*=NS 1e. 35 (14%) vs 31 (17%) vs 34 (19%) vs 20 (12%); 29 (46) vs 45 (51) vs 47 (51) vs 45 (51); *P*=NS  2. 234/2.0 (1.1) vs 255/2.1 (1.1) vs 243/1.9 (1.0) vs 245/2.1 (1.1)  3. No data reported. | All reported as pharmacist vs physician vs pharmacist + physician vs control groups at 12 months.  **1. Mean (SD) overall composite quality of life score (primary). (n=116 vs 124 vs 116 vs 127 patients)**  All other outcomes were secondary.  2. Mean (SD) short-form 36 subscale scores (n=116 vs 124 vs 116 vs 127 patients).  2a. Physical function.  2b. Role physical.  2c. Pain.  2d. General health.  2e. Vitality.  2f. Social function.  2g. Role emotional.  2h. Mental health.  3. Bulpitt subscales (%) (n=116 vs 124 vs 116 vs 127 patients).  3a. Faint.  3b. Faint on standing.  3c. Faint in the morning.  3d. Sleepy.  3e. Weak.  3f. Blurry vision.  3g. Short of breath.  3h. Swollen ankles.  3i. Walk slowly.  3j. Loose bowel movements.  3k. Dry mouth  3l. Dysphagia.  3m. Bad taste in mouth.  3n. Runny nose.  3o. Poor concentration.  3p. Flushing of face or neck.  3q. Nightmares.  3r. Nausea or vomiting.  3s. Rash.  3t. Itching.  3u. White fingers.  3v. Finger pain.  3w. Headache.  3x. Dry cough.  3y. Libido decreased.  3z. Erectile dysfunction.  4. Mean (SD) number of emergency department visits per patient (n=180 vs 181 vs 180 vs 171 patients).  4a. All.  4b. Heart disease specific.  5. Mean (SD) number of hospitalizations per patient (n=180 vs 181 vs 180 vs 171 patients).  5a. All.  5b. Heart disease specific.  6. Mean (SD) SDBP (mm Hg) (n=128 vs 126 vs 129 vs 124 patients).  6a. Baseline.  6b. Last 6 months.  7. Mean (SD) DBP (mm Hg) (n=128 vs 126 vs 129 vs 124 patients).  7a. Baseline.  7b. Last 6 months.    Not prespecified  8. Deaths (n=180 vs 181 vs 180 vs 171 patients). | 1. 37 (21) vs 35 (20) vs 38 (22) vs 36 (21); *P*=NS  2a. 48 (29) vs 52 (28) vs 45 (30) vs 49 (28), *P*=NS 2b. 53 (41) vs 49 (42) vs 46 (44) vs 44 (44), *P*=NS 2c. 51 (29) vs 53 (27) vs 45 (28) vs 48 (27), *P*=NS 2d. 46 (23) vs 51 (24) vs 45 (24) vs 46 (24), *P*=NS 2e. 46 (21) vs 48 (23) vs 43 (24) vs 45 (23), *P*=NS 2f. 72 (29) vs 75 (27) vs 68 (32) vs 70 (29), *P*=NS 2g. 66 (43) vs 70 (41) vs 64 (44) vs 66 (43), *P*=NS 2h. 66 (23) vs 70 (21) vs 62 (24) vs 65 (22), *P*=NS  3a. 42% vs 43% vs 47% vs 42%, *P*=NS 3b. 17% vs 19% vs 23% vs 23%, *P*=NS 3c. 22% vs 12% vs 14% vs 18%, *P*=NS 3d. 72% vs 71% vs 73% vs 75%, *P*=NS 3e. 52% vs 59% vs 61% vs 54%, *P*=NS 3f. 38% vs 40% vs 44% vs 38%, *P*=NS  3g. 49% vs 36% vs 45% vs 44%, *P*=NS 3h. 51% vs 46% vs 49% vs 43%, *P*=NS 3i. 45% vs 42% vs 47% vs 39%, *P*=NS 3j. 46% vs 40% vs 44% vs 38%, *P*=NS 3k. 49% vs 49% vs 59% vs 50%, *P*=NS 3l. 24% vs 20% vs 29% vs 28%, *P*=NS 3m. 45% vs 40% vs 43% vs 48%, *P*=NS 3n. 53% vs 53% vs 58% vs 54%, *P*=NS 3o. 22% vs 18% vs 21% vs 23%, *P*=NS 3p. 20% vs 20% vs 23% vs 22%, *P*=NS 3q. 31% vs 30% vs 34% vs 35%, *P*=NS 3r. 32% vs 26% vs 26% vs 25%, *P*=NS 3s. 15% vs 16% vs 18% vs 16%, *P*=NS 3t. 30% vs 36% vs 44% vs 37%, *P*=NS 3u. 25% vs 17% vs 22% vs 17%, *P*=NS 3v. 13% vs 13% vs 15% vs 13%, *P*=NS 3w. 52% vs 46% vs 49% vs 51%, *P*=NS 3x. 37% vs 37% vs 37% vs 34%, *P*=NS 3y. 34% vs 40% vs 29% vs 28%, *P*=NS 3z. 33% vs 41% vs 42% vs 42%, p=NS *P*=NS  4a. 1.11 (1.94) vs 1.02 (1.67) vs 1.01 (3.03) vs 1.21 (2.04); *P*=NS 4b. 0.02 (0.13) vs 0.01 (0.07) vs 0.01 (0.07) vs 0.04 (0.20); *P=*.02 for intervention groups vs control group  5a. 0.25 (0.62) vs 0.25 (0.69) vs 0.19 (0.74) vs 0.25 (0.89); *P*=NS 5b. 0.01 (0.07) vs 0.01 (0.10) vs 0.01 (0.11) vs 0.02 (0.13); *P*=NS 6a. 144 (18) vs 143 (20) vs 143 (17) vs 142 (16); *P*=NS 6b. 144 (21) vs 144 (18) vs 142 (23) vs 143 (18); *P*=NS  7a. 78 (10) vs 75 (12) vs 76 (11) vs 78 (10); *P*=NS 7b. 77 (11) vs 75 (12) vs 77 (14) vs 78 (11); *P*=NS  8. 1% vs 2% vs 1% vs 1% | 0 | 0 |
| Montgomery, 2000[39] | **1. Number (%) patients prescribed CV drugs (secondary outcome although primary follow-up period is 12 months):**  baseline (%) / 6 months (%). CCDSS + chart (n=207) vs chart only (n=208) vs usual care (n=137).  1a. 0-1 drug classes prescribed.  1b. 2 drug classes prescribed.  1c. ≥3 drug classes prescribed. | 1. chi square (4 df)=5.46; *P=*.24.  1a. 88 (43%)/81 (39%) vs 98 (47%)/68 (33%) vs 58 (42%)/50 (37%)  1b. 75 (36%)/74 (36%) vs 58 (28%)/67 (32%) vs 45 (33%)/47 (34%)  1c. 44 (21%)/52 (25%) vs 52 (25%)/73 (35%) vs 34 (25%)/40 (29%) | **1. Number (%) of patients with 5-year CV risk ≥10% (secondary): Baseline/ 12 months; adjusted OR (95% CI).**  1a. CCDSS + chart vs chart only.  **1b. CCDSS + chart vs usual care.**  1c. Chart only vs usual care.  2. Number (%) of patients with 5-year CV risk by group: Baseline / 12 months. CCDSS + chart vs Chart only vs Usual care.  2a. <10% CV risk.  2b. 10-19.9% CV risk.  2c. ≥20% CV risk.  3. CV risk score: Mean (SD) baseline / 12 months; mean difference [SE].  CCDSS + chart vs chart only vs usual care.  **4. Mean (SD) SBP (secondary): baseline / 12 months; difference [SE]. CCDSS + chart vs chart only vs usual care**  **5. Mean (SD) DBP (secondary): baseline / 12 months; difference [SE]. CCDSS + chart vs chart only vs usual care.**  Not prespecified  6. Change in mean absolute risk at 12 months. CCDSS + chart vs chart only vs usual care.  6a. Baseline risk <10%.  6b. Baseline risk 10-19.9%.  6c. Baseline risk ≥ 20%.  6d. All. | 1. Number (%) calculated by RA from data in article.  1a. 189/229 (83%) / 179/202 (89%) vs 198/228 (87%) / 169/199 (85%); 2.3 (1.1 to 4.8), *P=*.02  1b. 189/229 (83%) / 179/202 (89%) vs 138/157 (88%) / 114/130 (88%); 1.7 (0.7 to 3.9), *P=*.22  1c. 198/228 (87%) / 169/199 (85%) vs 138/157 (88%) / 114/130 (88%); 0.7 (0.3 to 1.6), *P=*.43  2a. 40 (17%) / 23 (11%) vs 30 (13%) / 30 (15%) vs 19 (12%) / 16 (12%)  2b. 112 (49%) / 114 (56%) vs 107 (47%) / 91 (46%) vs 82 (52%) / 60 (46%)  2c. 77 (34%) / 65 (32%) vs 91 (40%) / 78 (39%) vs 56 (36%) / 54 (46%).  3. 16.0 (8.3) / 16.7 (7.8); 0.65 [0.39] vs 17.9 (8.4) / 17.5 (8.2); -0.48 [0.35] vs 17.3 (8.6) / 17.8 (9.3); 0.77 [0.37]  4. 153 (19) / 153 (17); -0.04 [1.4] vs 156 (19) / 153 (19); -2.66 [1.4] vs 158 (21) / 159 (22); 0.25 [1.7]  Chart only vs usual care mean difference 4.6 mm Hg; 95% CI = 0.8 to 8.4 mm Hg, *P=*.02  5. 85 (9) / 85 (9); 0.36 [0.74] vs 87 (9) / 86 (10); -1.1 [0.78] vs 86 (11) / 84 (11); -1.64 [1.03]  6. Test for interaction between trial arm and baseline risk: F(2, 524)=4.88, *P<*.01  6a. 3.8 vs 2.3 vs 0.9  6b. 1.5 vs 0.7 vs 1.8  6c. -1.7 vs -1.7 vs -0.3  6d. 0.7 vs -0.5 vs 0.8 | 0 | 0 |
| Rossi, 1997[40] | Main outcome for 6-month study. **1. Prescription changes from a calcium channel blocker to another antihypertensive agent: n/N of patients (%). 1a. Overall.** 1b. Changed to β-blockers. 1c. Changed to diuretics. 1d. Increased ACE-I dose. 1e. Changed to both β-blockers and diuretics. 1f. No other medication substituted. | 1a. 39/346 (11.3%) vs 1/373 (<1%), *P<*.0001 1b. 26/346 vs 1/373 1c. 7/346 vs 0/373 1d. 3/346 vs 0/373 1e. 2/346 vs 0/373 1f. 1/346 vs 0/373 | … | … | + | … |
| McAlister, 1986[41] | No outcomes clearly prespecified  **1. Mean length of follow up (days) by physicians with patients from first to last visit (95% CI) (16 month Follow Up)  1a. All patients** 1b. Moderate hypertension 1c. Mild hypertension 1d. Newly diagnosed  **2. Mean % of patients treated for hypertension (95% CI) (16 month Follow Up) 2a. All patients** 2b. Moderate hypertension 2c. Mild hypertension 2d. Newly diagnosed  **3.Mean no. of office visits per patient-year (95% CI) (16 month follow up) 3a. All patients** 3b. Moderate hypertension 3c. Mild hypertension 3d. Newly diagnosed  All patients: baseline DBP > 90 mmHg or prescribed antihypertensive medication. Moderate hypertension: baseline DBP >104 mmHg Mild hypertension: baseline DBP >90 to <105 mmHg | 1a.199.3 (173.0-225.6) vs 167.0 (148.8 -193.2); *P<*.09 (not significant at *P=*.05 but significant at *P<*.1) 1b.168.0 (141.0-195.0) vs 152.7 (121.1-184.3) ; NS 1c.190.9 (163.6 – 218.1) vs 169.3 (137.7-209.9); NS 1d.162.0 (137.5-186.5) vs 132.1 (108.0-156.2); *P<*.1  2a.95.4 (87.1-100*) vs 95.7 (87.7 -100) ; NS 2b.95.1 ( 86.6-100) vs 84.5 (70.3-98.7) ; NS 2c.91.4 (80.4-100) vs 90.2 (78.5-100) ; NS 2d.79.4 (63.5-95.3) vs 76.1 (59.4 -92.8) ; NS  3a.10.8 (9.2-12.4) vs 12.4 (9.8 – 15.0); NS 3b.13.3 (11.0-15.6) vs 17.4 (13.9-20.9); *P<*.09 3c.11.6 (11.2-12.0) vs 12.7 (12.1-13.3; NS 3d.13.1 (11.5-14.7) vs 14.7 (11.7-17.7); NS  *Upper 95% CI truncated at 100% | No outcomes clearly prespecified  **1.Mean % of patients with DBP ≤90 mmHg on last visit at 16 months (95% CI) 1a. All patients** 1b. Moderate hypertension 1c. Mild hypertension 1d. Newly diagnosed  **2.Mean number of days with DBP ≤90 mmHg per patient-year at 16 months (95% CI) 2a. All patients** 2b. Moderate hypertension 2c. Mild hypertension 2d. Newly diagnosed  **3.Mean change in median DBP (mmHg) from baseline to last visit (95% CI) 3a. All patients** 3b. Moderate hypertension 3c. Mild hypertension 3d. Newly diagnosed | 1a. 88.9 (76.5-100) vs 87.5 (74.5-100); NS 1b. 86.0 (72.4-99.6) vs 76.2 (59.5-92.9); NS 1c. 87.9 (75.1-100) vs 88.3 (75.7-100); NS 1d. 92.4 (82.0-100) vs 91.5 (80.6-100); NS  2a. 215.6 (175.1-256.1) vs 202.6 (160.8-244.4); NS 2b. 191.7 (136.6-246.8) vs 175.7 (119.1-232.3); NS 2c. 251.0 (205.7-296.3) vs 274.0 (229.5-318.5); NS 2d. 323.2 (299.7-346.7) vs 258.5 (212.8-304.2); *P<*.03   3a. -4.9 (-6.6 to -3.2) vs -4.1 (-6.1 to -2.1); NS 3b. -21.7 (-25.1 to -18.3) vs -16.7 (-19.9 to -13.5); *P<*.06 3c. -9.8 (-11.9 to -7.7) vs -8.5 (-10.8 to -6.2); NS 3d. -15.1 (-18.2 to -12.0) vs -11.3 (14.2 to -8.4); NS | 0 | 0 |
| Rogers, 1984[42-44] | Prespecified **1. Proportion of hypertension patients with medical care event at 1 year / 2 year / both years / not done. 1a. Renal function exam.  1b. Potassium exam.  1c. Fundoscopic exam.  1d. Intravenous pyelogram.**   **2. Proportion of obesity patients with medical care event at 1 year / 2 years / both years / not done. 2a. Number of diets given or reviewed overall.**  2b. Number of diets given or reviewed for men.  2c. Number of diets given or reviewed for women.   **3. Proportion of patient with renal disease and medical care events at 1 year / 2 years / both years / not done. 3a. Renal function exam (blood urea nitrogen, creatinine or creatinine clearance).  3b. Urine analysis.  3c. Urine culture.**  **4. Mean perceived quality of communication score over 1 year adjusted for financial status, chart weight, prior clinic attendance length, and age (high scores better).**  Not clearly prespecified:  5. Mean (±95% CI) number of events in subgroup of patients with hospitalization data at year 1 / year 2 / combined. (p-value for years 1 and 2 combined) 5a. Procedures and referrals carried out.  5b. Diets by Cardiac, Pulmonary, and Renal Clinics.  5c. New problems indicated by Cardiac, Pulmonary, and Renal (CPR) Clinics clinics.  5d. Resolved problems.  5e. New abnormal lab results.  5f. Worse abnormal lab results.   Note: Data inconsistency. Text (p.67, 1982 paper) indicates urine analysis significant and urine culture not significant. Table 3 states the opposite. Text appears to be correct  6. Proportion of times a diagnostic intervention result was recorded for patients with length of hospitalization available (year 1 / year 2). 6a. chest x-ray 6b. electrocardiogram 6c. urine analysis 6d. red blood cells 6e. Hb 6f. Haematocrit test (cell pack) 6g. White blood count 6h. blood smear 6i. Venereal disease research laboratory 6j. Blood urea nitrogen 6k. uric acid 6l. creatinine 6m. fasting blood sugar 6n. PCS (2 hr) 6o. cholesterol 6p. sodium 6q. potassium 6r. chlorides 6s. carbon dioxide 6t. pap smear 6u. all tests | 1a. 22.3% vs 20.5% / 9.1% vs 14.1% / 60.9% vs 50.3% / 7.6% vs 15.1%, *P=*.03 1b. 23.3% vs 20.5% / 10.2% vs 13.0% / 60.4% vs 52.5% / 6.1% vs 14.1%, *P=*.042  1c. 9.5% vs 3.2% / 59.8% vs 52.6% / 7.0% vs 4.7% / 27.9% vs 37.8%, *P*>.05  1d. 6.5% vs 6.8% / 22.6% vs 31.6% / 39.2% vs 31.1% / 31.0% vs 28.6%, *P*>.05  2a. 16.2% vs 11.4% / 29.4% vs 20.3% / 33.8% vs 20.3% / 20.6% vs 48.1%, *P=*.007  2b. 15.0% vs 6.7% / 45.0% vs 6.7% / 30.0% vs 46.7% / 10.0% vs 40.0%, *P*>.05 2c. 16.7% vs 12.5% / 22.9% vs 23.4% / 35.4% vs 14.1% / 25.0% vs 50.0%, *P=*.018    3a. 18.8% vs 13.3% / 3.1% vs 13.3% / 70.3% vs 55.6% / 7.8% vs 17.8%, *P*>.05 3b. 32.8% vs 20.0% / 10.9% vs 20.0% / 46.9% vs 31.1% / 9.4% vs 28.9%, *P=*.015 3c. 48.4% vs 60.0% / 9.4% vs 11.1% / 25.0% vs 20.0% / 17.2% vs 8.9%, *P*>.05   4. No data reported (figure 2b in article), *P<*.05 in favour of CCDSS.  5a. 31.8 (5.8) / 40.9 (11.3) / 35.5 (5.7) vs 17.2 (5.3) / 32.4 (11.2) / 24.0 (5.9), *P<*.005 5b. 0.3 (0.2) / 0.3 (0.2) / 0.3 (0.1) vs 0.1 (0.1) / 0.1 (0.1) / 0.1 (0.1), *P<*.03 5c. 1.0 (0.4) / 0.9 (0.3) / 1.0 (0.3) vs 0.6 (0.3) / 0.4 (0.3) / 0.5 (0.2), *P<*.007 5d. 0.2 (0.1) / 0.3 (0.2) / 0.2 (0.1) vs 0.0 (0.1) / 0.0 (0.1) / 0.0 (0.1), *P*=NS 5e. 3.9 (1.0) / 5.4 (1.8) / 4.5 (0.9) vs 2.0 (0.9) / 5.0 (1.9) / 3.4 (1.0), *P*=NS 5f. 1.5 (0.6) / 1.6 (1.3) / 2.0 (0.6) vs 1.4 (0.9) / 1.7 (1.0) / 1.6 (0.6), *P*=NS  6a. 82.1% / 84.6% vs 54.3% / 55.6% 6b. 84.6% / 80.8% vs 57.1% / 63.0% 6c. 81.6% / 80.8% vs 48.6% / 66.7% 6d. 71.8% / 73.1% vs 42.9% / 59.3% 6e. 82.1% / 73.1% vs 51.4% / 70.4% 6f. 82.1% / 73.1% vs 57.1% / 66.7% 6g. 87.2% / 73.1% vs 51.4% / 70.4% 6h. 69.2% / --- vs 28.6% / --- 6i. 25.6% / 38.5% vs 20.0% / 22.2% 6j. 87.2% / 88.5% vs 57.1% / 77.8%  6k. 84.6% / 84.6% vs 37.1% / 63.0% 6l. 87.2% / 84.6% vs 42.9% / 63.0% 6m. 84.6% / 88.5% vs 42.9% / 81.5% 6n. 18.4% / 23.1% vs 08.6% / 18.5% 6o. 87.2% / 84.6% vs 45.7% / 63.0% 6p. 82.1% / 88.5% vs 54.3% / 74.1% 6q. 82.1% / 88.5% vs 65.7% / 77.8% 6r. 82.1% / 88.5% vs 54.3% / 74.1% 6s. 82.1% / 88.5% vs 51.4% / 74.1%  6t. 61.9% / 62.5% vs 40.0% / 22.7% 6u. 75.3% / 76.2% vs 45.6% / 61.3% *P*=NR | **Prespecified 1. Mean perceived health status over 1 year adjusted for financial status, chart weight, prior clinic attendance length, and age (high scores better).**   Not clearly prespecified. Data collected by retrospective chart review using a standardised evaluation form. Not clear which data were intended as outcomes for analysis or if some analyses were post-hoc decisions.. 2. Proportion of deaths by study end. 2a. Hypertension patients.  2b. Obesity patients.  2c. Renal disease patients.   3. Mean adjusted SBP / DBP in hypertension patients. Adjusted for BP at start of study, age, and previous time in cardiac-pulmonary-renal clinics. Unadjusted data with 95% CIs was also reported in 1982 paper. 3a. Men after 10-15 months.  3b. Women after 10-15 months.  3c. Men after 22-24 months.  3d. Women after 22-24 months.  4. Mean adjusted pounds overweight in obesity patients. Adjusted for pounds overweight at baseline, ideal weight, time in cardiac-pulmonary-renal clinics, concomitant diabetes, and total number of other concomitant diseases. Unadjusted data with 95% CIs was also reported in 1982 paper. 4a. Men / women at 10-15 months.  4b. Men / women at 22-24 months.   5. Proportion of patients with normal/abnormal renal test during year 2 (excluding those that did not have test).  5a. Renal function exam (blood urea nitrogen, creatinine or creatinine clearance).  5b. Urine analysis.  5c. Urine culture.   6. Number of patients hospitalised at 1 year / 2 years (adjusted for previous cardiac-pulmonary-renal clinic attendance, diabetes, and sex.).   7. Mean adjusted length of hospital stay (days) at 1year / 2 years.  7a. Outliers included 7b. outliers excluded  Not prespecified. 8. Proportion of patients newly diagnosed during study. 8a. Hypertension.  8b. Obesity.  8c. Renal disease.  Note: results for newly diagnosed hypertension and obesity patients generally consistent with those for all patients (4 and 5 above), although at 10-15 months CDSS patient less overweight (22.1-28.2 lbs vs 36.7-42.6, *P<*.04).   9. Proportion of admitted patients with various admission diagnoses – tests / pregnancy, cosmetic surgery / acute illness or surgery with no evidence of complications due to chronic disease / evidence of chronic disease with mild impairment of function / evidence of chronic disease with severe impairment of function / condition critical, evidence of life-endangering disease / total number of patients admitted 9a. year 1 9b. year 2 | 1. No data reported (figure 2b in article), *P<*.05 in favour of CCDSS.  2a. 14.2% vs 17.8%, *P*>.05 2b. 1.5% vs 8.6%, *P*>.05 2c. 15.6% vs 22.2%, *P*>.05   3a. 147.7 / 91.5 vs 151.8 / 91.4, *P*=NS 3b. 148.6 / 91.7 vs 146.5 / 91.3, *P*=NS 3c. 144.5 / 90.1 vs 146.8 / 94.0, *P*=NS 3d. 146.9 / 91.3 vs147.0 vs 90.1, *P*=NS   4a. 45.6 / 52.3 vs 48.6 / 55.3, *P=*.12 4b. 39.3 / 51.5 vs 52.2 / 55.8, *P=*.023   5a. 36.2% / 63.8% vs 22.6% / 77.4%, *P=*.10 5b. 45.9% / 54.1% vs 4.3% / 95.7%, *P<*.001 5c. 68.2% /31.8% vs 35.7% /64.3%, *P=*.028   6. 48/40 vs 41/40, *P*>.05  7a. 20.0 / 9.7 vs 16.5 / 20.7, *P*>.05 (*P<*.01 for interaction of CDSS and year).  7b. 17.8 / 13.5 vs 19.0 / 20.9  8a. 5.8% vs 4.2%, *P*>.05  8b. 5.4% vs 8.4%, *P*>.05 8c. 0% vs 0%  9a. 0.05 / 0.04 / 0.51 / 0.24 / 0.12 / 0.02 / 49 vs 0.10 / 0.00 / 0.37 / 0.20 / 0.32 / 0.02 / 41 (NS) 9b. 0.10 / 0.00 / 0.51 / 0.18 / 0.18 / 0.03 / 61 vs 0.05 / 0.00 / 0.39 / 0.18 / 0.34 / 0.05 / 44 (NS) | + | + |
| Coe, 1977[45] | ... | … | BP measures were prespecified; other measures were not clearly prespecified.   **1a. Number of patients that achieved adequate BP control (DBP <95 mmHg during treatment).** 1b. Number of patients that achieved incomplete but substantial BP control (DBP 95-105 mmHg during treatment). 1c. Number of patients that did not achieve BP control (DBP >105 mmHg during treatment).  2. Mean (SEM) BP measurements. 2a. SBP/DBP mmHg overall: pretreatment; reduction after treatment.  2b. Mean (SEM) SBP pretreatment/post-treatment in patients with DBP <95 mmHg during treatment. 2c. Mean (SEM) DBP pretreatment/post-treatment in patients with DBP <95 mmHg during treatment. 2d. Mean (SEM) SBP pretreatment/posttreatment in patients with DBP 95 to 105 mmHg during treatment. 2e. Mean (SEM) DBP pretreatment/posttreatment in patients with DBP 95 to 105 mmHg during treatment. 2f. Mean (SEM) SBP pretreatment/posttreatment in patients with DBP >105 mmHg during treatment. 2g. Mean (SEM) DBP pretreatment/posttreatment in patients with DBP >105 mmHg during treatment.  3. Time in compliance, %. 3a. For patients with DBP <95 mmHg during treatment. 3b. For patients with DBP 95 to 105 mmHg during treatment. 3c. For patients with DBP >105 mmHg during treatment.   4. Weeks of treatment, mean (value appears to be mean but this is not explicit) (SEM). 4a. For patients with DBP <95 mmHg during treatment. 4b. For patients with DBP 95 to 105 mmHg during treatment. 4c. For patients with DBP >105 mmHg during treatment.  5. Weeks of compliance, mean (value appears to be mean but this is not explicit) (SEM). 5a. For patients with DBP <95 mmHg during treatment. 5b. For patients with DBP 95 to 105 mmHg during treatment. 5c. For patients with DBP >105 mmHg during treatment.  6. Number of patients with side effects from different anti-hypertensive drugs. I.Thiazide (n=NR) a. Gout II.Alphamethyldopa (n=26 vs 21) a. Somnolence b. Syncope c. Depression d. Reaction e. Cannot take f. No higher dose III. Guanethidine (n=19 vs 9) a. Postural dizziness b. Syncope c. Impotence d. Diarrhoea e. Reaction  Note: Type of drugs prescribed in each group also reported by final DBP control (Table 3 in article). | 1a. 23/56 vs 30/60 1b. 17/56 vs 20/60 1c. 16/56 vs 10/60 Authors report “blood pressure…response was similar for both groups, as were drug side effects and overt non-compliance with treatment.” 2a. 172(3)/113(2) vs 167(4)/111(2); 19.5(2.5)/13.4(1.4) vs 18.3(3.3)/14.5(1.4) Note: *P<*.02 for difference in CCDSS and control regression slopes for SBP; no difference reported for DBP.  2b. 165(4)/142(3) vs 162(5)/136(3) 2c. 105(2)/90(0.9) vs 107(2)/89(0.9) 2d. 167(5)/151(6) vs 163(7) /154(4) 2e. 110(2)/100(0.7) vs 108(2)/98(0.6)  2f. 187(5)/168(5) vs 189(11)/173(7)  2g. 129(3)/112(2) vs 129(4)/116(3)   3a. 74.2% vs 79.6% 3b. 72.8% vs 58.6% 3c. 54.3% vs 44.6%  4a. 20.9 (3.3) vs 24.8 (2.8) 4b. 28.6 (3.7) vs 39.6 (2.3) 4c. 35.7 (2.9) vs 22.8 (6.1)   5a. 15.5 (2.7) vs 19.8 (2.6) 5b. 20.8 (3.4) vs 23.2 (3.1) 5c. 19.4 (2.8) vs 10.2 (1.7)    6Ia. 1 vs 2 6IIa. 16 vs 13 6IIb. 3 vs 0 6IIc. 2 vs 1 6IId. 0 vs 3 6IIe. 2 vs 1 6IIf. 1 vs 2 6IIIa.12 vs 2 6IIIb. 2 vs 0 6IIIc. 1 vs 0 6IIId. 1 vs 0 6IIIe. 1 vs 0 | … | 0 |
| **Asthma and COPD** | | | | | | |
| Fiks, 2009[46] | Primary outcomes over 6 month intervention. 1. Change in rates of captured opportunities for vaccination (visit-level analysis). Pre to post study, difference (95% CI).  1a. Unadjusted rates.  **1b. Rates adjusted for selected covariates.**   2. Up-to-date vaccination rates (patient-level analysis). Pre to post study, difference (95% CI). 2a. Unadjusted rates.  **2b. Rates adjusted for selected covariates.**   Secondary outcomes over 6 months.  3. Difference (95% CI) in proportion of children who had ≥1 vaccine dose (intervention vs control).   4. Improvement in vaccination rate at intervention sites versus control sites. 4a. Children who had received the influenza vaccine previously 4b. Children who had not received the influenza vaccine previously. | 1a. 14.4% to 19.2% vs 12.3% to 16.1%, 1% (-2.4 to 4.9)  1b. 14.4% to 18.6% vs 12.7% to 16.3%, 0.3% (-1.9 to 2.5).  2a. 45% to 53% vs 44.2% to 48.2%, 4.0% (-1.3 to 9.1)  2b. 45.7% to 51% vs 46% to 47.9%, 3.4% (-1.4 to 9.1)  3. 4.0% (-1.1 to 10.7)  4. Overall *P*=.61 4a. 6.5% 4b. 3.2% | ... | … | 0 | … |
| Poels, 2009[47] | **1. Proportion (95% CI) of diagnoses that changed after intervention; Odds ratio (95% CI), p-value, for CCDSS vs usual care(primary)**  2. Proportion of patients who were referred to a specialist; Odds ratio (95% CI), p-value, for CCDSS vs usual care (secondary)  3. Proportion of additional diagnostic tests ordered; Odds ratio (95% CI), p-value, for CCDSS vs usual care (secondary)  4. Proportion of patients who had their medication changed; Odds ratio (95% CI), p-value, for CCDSS vs usual care (secondary)  5. Shift in diagnosis from COPD to another diagnosis. Odds Ratio (95% CI) for CCDSS vs Usual Care (not pre-specified)  6. Shift in diagnosis from asthma to another diagnosis. Odds Ratio (95% CI) for CCDSS vs Usual Care (not pre-specified)  7. Shift in diagnosis from “no respiratory disease” to another diagnosis. Odds Ratio (95% CI) for CCDSS vs Usual Care (not pre-specified)  9. Number of patients with no diagnosis after interpretation. (Not pre-specified)  9. Of all patients for whom practitioner reported no diagnosis after interpretation, proportion with each reason (not pre-specified)  9a. Standard assessment form was lost  9b. Patients had left the practice  9c. Patients had died  9d. Patients were under treatment from a chest physician  9e. Practitioners could not interpret the spirometry results  9f. Other reasons  10. Odds ratio (95% CI) for change in diagnosis after intervention (CCDSS vs Usual Care) (not pre-specified)  10a.respiratory disease  10b. apparent respiratory disease | CCDSS vs Chest Physician Support vs. Usual care  **1. 45.0% (39.5 to 50.6) vs 47.8% (41.8 to 53.9) vs 53.3% (47.2 to 59.4); 0.72 (0.45 to 1.15),** *P=***.16**  2. 5.7% vs 7.6% vs 5.2%; 1.09 (0.53 to 2.36), *P=*.82  3. 18.1% vs. 8.7% vs 12.5%; 1.61 (0.76 to 3.41), *P=*.21  4. 38.9% vs 32.7% vs 39.0%; 0.99 (0.65 to 1.52), *P=*.97  5. 0.88 (0.48 to 1.61)  6. 0.55 (0.27 to 1.12)  7. 0.85 (0.34 to 2.13)  8. 19 vs 25 vs 43  9a. 26.3% vs 16% vs 39.5%  9b. 15.8% vs 16% vs 9.3%  9c. 5.3% vs 8% vs 4.7%  9d. 0% vs 16% vs 7%  9e. 0% vs 12% vs 18.6%  9f. 19% vs 32% vs 43%  10a. 0.83 (0.48 to 1.43)  10b. 0.52 (0.27 to 1.01) | … | … | 0 | … |
| Martens, 2007[48, 49] | All measured during 12 month intervention period. 1. Appropriate prescribing when no prescribing of a particular drug was advised: % not prescribing [in accordance with recommendation] (95% CI)  1a. No statins for newly diagnosed patients with diabetes or CVD between 18 and 70 years with cholesterol <3.5mmol divided by all same population considered for prescription.  **2. Appropriate prescribing for asthma/COPD when no prescribing of a particular drug was advised: volume per general practitioner per 1000 enlisted patients. (95% CI)**  2a. Prescriptions for intermittent asthma and maintenance treatment.  2b. Inhaled corticosteroids for newly diagnosed COPD in patients >40 years.  **2c. Sum score for asthma/COPD prescriptions (primary).**  **3. Appropriate prescribing of statins for patients with newly diagnosed diabetes mellitus or CVD, 18-70 years of age, and cholesterol <3.5mmol, when no prescribing of a particular drug was advised: volume per general practitioner per 1000 enlisted patients. (95% CI) (primary).**  4. Appropriate prescribing when prescribing of a particular drug was advised: % prescribing [in accordance with recommendation] (95% CI)  4a. benzolyperoxi and salicylacid for acne vulgaris divided by all prescriptions for acne vulgaris. 4b. erythromycin, minocyclin, cyproteronacetate for acne vulgaris divided by all prescriptions for acne vulgaris. 4c. minocyclin, benzoylperoxi, salicyl acid for acne vulgaris (comedones with inflammation, symptoms) divided by all prescriptions for acne. 4d. co-trimoxazol, ciprofloxacin and norfloxacin for chronical and recurrent symptoms on prostatitis divided by all antibacterial antiobiotic prescriptions for same condition. 4e. trimethoprim, nitrofurantoin for acute and recurrent cystitis among female patients >12 years divided by all prescriptions for same population. 4f. Terbutalin turbohaler/salbutamol diskus/salbutamoldosis-aerosol for intermittent/mildly persistent and moderate persistent asthma with acute complaints among patients >7 years divided by all asthma prescriptions for same population. 4g. Budesonide turbuhaler/fluticason discus/fluticasondosis-aerosol for mildly persistent asthma with maintenance treatment among patients >7 years divided by all asthma prescriptions for same population.  4h. Budesonide turbuhaler/fluticason diskus/fluticason dosis-aerosol AND: salmeterol discus/salmeterol dosis-aerosol/formoterol dosis-aerosol for severe persistent asthma with maintenance treatment among patients >7 years divided by all asthma prescriptions for same population. 4i. ipratropiumbromid powder inhaler, ipratropiumbromid dosis-aerosol, salbutamol discus, salbutamol dosis-aerosol for newly diagnosed COPD patients >40 years divided by all prescriptions for COPD patients >40 years of age.  4j. statins for newly diagnosed patients with diabetes or CVD between 18 and 70 years and cholesterol >5.5mmol divided by all statin prescriptions for newly diagnosed diabetes mellitus or CVD.  5. Appropriate prescribing of particular antibiotics: volume per general practitioner per 1000 enlisted patients. (95% CI)  5a. benzolyperoxi and salicylacid for acne vulgaris (mainly comedones).  5b. erythromycin, minocyclin, cyproteronacetate for acne vulgaris (mainly inflammation, symptoms).  5c. minocyclin, benzoylperoxi, salicyl acid for acne vulgaris (comedones with inflammation, symptoms).  5d. co-trimoxazol, ciprofloxacin and norfloxacin for chronical and recurrent symptoms on prostatitis.  5e. trimethoprim, nitrofurantoin for acute and recurrent cystitis among female patients >12 years.  **6. Appropriate prescribing of particular drugs for asthma/COPD treatment: volume per general practitioner per 1000 enlisted patients. (95% CI) (primary).**  6a. Terbutalin turbohaler/salbutamol diskus/salbutamol dosis-aerosol for intermittent/mildly persistent and moderate persistent asthma with acute symptoms among patients >7 years.  6b. Budesonide turbuhaler/fluticason discus/fluticason dosis-aerosol for mildly persistent asthma with maintenance treatment among patients >7 years.  6c. Budesonide turbuhaler/fluticason diskus/fluticason dosis-aerosol AND: salmeterol discus/salmeterol dosis-aerosol/formoterol dosis-aerosol for severe persistent asthma with maintenance treatment among patients >7 years.  6d. ipratropiumbromid powder inhaler, ipratropiumbromid dosis-aerosol, salbutamol discus, salbutamol dosis-aerosol for newly diagnosed COPD patients >40 years  **6e. Sum score for asthma/COPD drug prescriptions (primary).**  **7. Appropriate prescribing of particular cholesterol-lowering drugs: volume per general practitioner per 1000 enlisted patients. (95% CI) (primary).**  Note: also reports volume of prescriptions for all antibiotics, % of prescriptions for inhaled corticosteroids in asthma patients, and volume of prescriptions for inhaled corticosteroids in asthma patients; however, only reports data for ‘clinically meaningful’ results. | 1a. 100% (0) vs 98% (94–100): NS  2a. 1.1 (0.5 to 2.3) vs 1.7 (0.8 to 3.3) 2b. 0 (0.0 to 0.1) vs 0.5 (0.3 to 0.9), *P*<.05 2c. 1.1 (0.6 to 2.6) vs 2.2 (1.4 o 4.3), NS  3. 0 vs 0.1 (0.0 to 0.2), NS  4a. 19% (7 to 38) vs 24% (9 to 49): NS 4b. 59% (42 to 72) vs 68% (56 to 77): NS 4c. 50% (32 to 73) vs 35% (17 to 52): NS 4d. 47% (23 to 65) vs 53% (24 to 81): NS 4e. 73% (69 to 80) vs 57% (52 to 63); *P=*.01 4f. 47% (38 to 54) vs 51% (39 to 65): NS 4g. 44% (30 to 56) vs 27% (14 to 47): NS 4h. 36% (20 to 53) vs 51% (26 to 78): NS 4i. 15% (9 to 29) vs 15% (8 to 23): NS 4j. 88% (71 to 100) vs 72% (52 to 81): NS  5a. 0.3 (0.1 to 1.2) vs 0.3 (0.1 to 0.5)  5b. 1.9 (1.1 to 2.8) vs 2.0 (1.3 to 3.1) 5c. 0.6 (0.3 to 1.1) vs 0.4 (0.1 to 1.1) 5d. 0.8 (0.4 to 1.9) vs 0.4 (0.2 to 0.9) 5e. 10.1 (7.6 to 14.0) vs 11.5 (6.9 to 19.3)  6a. 3.3 (2.1 to 4.6) vs 4.8 (3.3 to 6.9)  6b. 1.7 (1.0 to 2.6) vs 1.4 (0.7 to 4.1) 6c. 0.3 (0.1 to 0.7) vs 0.5 (0.3 to 1.0) 6d. 0.7 (0.3 to 1.1) vs 1.0 (0.6 to 1.7)  6e. 5.9 (3.8 to 7.9) vs 7.7 (5.6 to 11.8), NS  7. 1.0 (0.5 to 2.2) vs 1.2 (0.7 to 1.8), NS | ... | … | 0 | … |
| Kattan, 2006[50] | Prespecified  **1. Number of weeks from the first scheduled provider visit after symptoms warranting a step-up in therapy to a step-up in medication use by percent of study participants. 1a. Entire 1 year period, p-value 2. Actions within 2 months of medication step-up recommendation.**  **2a. % scheduled visits.** | 1a.See figure 2 for graph, faster with CCDSS, *P=*.15  2a. 17.1% vs 12.3%, *P=*.005 | All reported as mean (SE); p-value **1. Maximum symptom days per 2 weeks (primary)** 2. Days limited in activities for more than half day per 2 weeks (not prespecified) 3. School days missed per 2 weeks (prespecified) 4. Number of emergency department visits per year (prespecified) 5. Number of unscheduled clinic visits per year (not prespecified) 6. Number of hospitalizations per year (prespecified)  Note: Data available for subgroup of 226 children who needed and received medication step-up. | 1. 3.43 (0.11) vs 3.52 (0.11); *P=*.54 2. 1.42 (0.07) vs 1.60 (0.08); *P=*.09 3. 0.67 (0.04) vs 0.72 (0.04); *P=*.38 4. 0.87 (0.07) vs 1.14 (0.08); *P=*.013 5. 1.14 (0.08) vs 1.31 (0.08); *P=*.14 6. 0.22 (0.03) vs 0.24 (0.03); *P=*.56 | + | 0 |
| Kuilboer, 2006[51] | **1. Median of paired differences of Delta values (the difference between the intervention and baseline periods) (p-value) for each age group: 0-11, 12-39, 40-59, ≥60.**  **Not clearly pre-specified**  **1a. Number of contacts**  **1b. Number of peak total flow measurements**  **1c. Number of peak flow ratio measurements**  **1d. Number of FEV1 total measurements**  **1e. Number of FEV1 ratio measurements**  **1f. Number of antihistamines prescriptions**  **1g. Number of cromoglycate prescriptions**  **1h. Number of deptropine prescriptions**  **1i. Number of oral bronchodilators prescriptions**  **1j. Number of oral corticosteroids prescriptions** | 1a. -0.164 (0.255), +0.154 (0.034), +0.068 (0.756), +0.257 (0.134)  1b. +0.020 (0.016), +0.029 (0.020), +0.028 (0.096), +0.005 (0.133)  1c. +0.000 (0.071), +0.402 (0.004), +0.181 (0.009), +0.000 (0.108)  1d. +0.005 (0.028), +0.005 (0.062), +0.004 (0.009), 0.000 (0.108)  1e. +0.000 (0.046), +0.056 ((0.010), +0.250 (0.010), +0.000 (0.016)  1f. 0.000 (0.875), 0.000 (0.500), -0.004 (0.080), -0.000 (0.317)  1g. 0.000 (0.144), -0.0004 (0.033), 0.000 (0.051), 0.000 (0.893)  1h. -0.003 (0.753), N/A, N/A, N/A  1i. 0.001 (0.807), 0.000 (0.655), 0.000 (0.121), 0.000 (0.225)  1j. -0.004 (0.050), -0.002 (0.836), -0.023 (0.109), -0.045 (0.679) | ... | ... | 0 | … |
| Plaza, 2005[52] | **Prespecified; 12-month follow-up. Use of the following health resources: 1. Spirometry 2. Conventional blood tests 3. Total immunoglobulin E 4. Skin allergy tests 5. Thorax radiography  6. Prescriptions of oral glucocorticoids**  **7. Number of medical visits during the study. 8. Number of home visits.  9. Number of visits to other physicians.** | 1. 79 vs 70, *P*>.10 2. 30 vs 18, *P* >.10 3. 21 vs 2, *P=*.10 4. 17 vs 7, *P* >.10 5. 23 vs 15, *P* >.10 6. 130 vs 727, *P=*.0135  7. 314 vs 367, *P* >.10 8. 42 vs 17, *P* >.10,  9. 96 vs 147, *P* >.10 | 12-month follow-up  1. Estimated increment of the cost-effectiveness coefficient (primary)  1a. From the social perspective.  1b. From the perspective of the one who pays.  Component of primary (prespecified outcome). 2. **St. George Respiratory Questionnaire score (score range 0 [no impairment] to 100 [maximum impairment]):  2a. total score (SE); difference (95% CI)** 2b. Activity (SE); difference (95% CI) 2c. Symptoms (SE); difference (95% CI) 2d. Impact (SE); difference (95% CI)  Prespecified  3. Number of emergency room visits. 4. Number of hospitalizations. 5. Days spent in intensive care unit. 6. Days hospitalised. 7. Days on rescue medication.  8. Number of short cycles of oral steroid use.  9. Number of patients symptom-free at the end of the study. | 1a. -135 per each point reduction in the SGRQ scale (95% CI -8374 to -38)  1b. -61 (95% CI -2323 to -14).  The negative sign of the coefficients was due to a minor cost and major reduction of the SGRQ in intervention group compared to the usual care group.  **2a. 27.3 (2.0) vs 34.1 (1.9), *P=*.002; 6.8 (2.5 to 11.1)** 2b. 35.6 (2.9) vs 44.4 (2.9), *P=*.005; 8.8 (2.7 to 14.8) 2c. 32.9 (1.9) vs 39.7 (1.8), *P=*.003; 6.8 (2.3 to 1.3) 2d. 20.7 (2.0) vs 26.3 (1.9), *P=*.001; 5.6 (1.2 to 10.1)  3. 49 vs 115, *P=*.09 4. 12 vs 15, *P*>.10 5. 8 vs 2, *P*>.10 6. 37 vs 166, *P*>.10 7. 3,478 vs 9,318, *P=*.03 8. 53 vs 95, *P*>.10  9. 49 vs 22 | 0 | + |
| Tierney, 2005[53] | Physician intervention vs pharmacist intervention vs both interventions vs control: Number of patients/grp, 194 vs 161 vs 182 vs 169  **Primary outcome 1. Number of suggestions adhered to/Number of patients with suggestions, %, of care suggestions adhered to over 3 yrs. 1a. Overall.**  1b. Influenza vaccination.  1c. Pneumococcal vaccination.  1d. Obtain pulmonary function test.  1e. Start ipratropium.  1f. Start inhaled β-agonist.  1g. Switch to cheaper β-agonist.  1h. Increase/decrease theophylline dose.  1i. Stop ipratropium.  1j. Start inhaled corticosteroid.  1k. Start oral corticosteroid.  Prespecified  2. Medication compliance measures.  2a. Mean Inui score (%).  2b. Mean (SD) Morisky score.  2c. N, %, of patients with ≥2 prescription refills.  2d. Mean (SD) medication possession ratio (measure referenced but not described).  3. Mean (SD) score for patient satisfaction with physician (American Board of Internal Medicine questionnaire; score range/direction not described).  4. Mean (SD) score for patient satisfaction with pharmacist (American Board of Internal Medicine questionnaire; score range/direction not described). | 1a. 161/498, 32% vs 123/382, 32% vs 173/471, 37% vs 135/416, 32%, *P*=NS 1b. 37/92, 40% vs 34/80, 43% vs 37/100, 37% vs 36/85, 42%, *P*=NS 1c. 7/89, 8% vs 6/76, 8% vs 15/95, 16% vs 7/78, 9%, *P*=NS 1d. 6/97, 6% vs 4/65, 6% vs 9/75, 12% vs 4/66, 6%, *P*=NS 1e. 30/71, 42% vs 15/59, 25% vs 23/65, 35% vs 17/67, 25%, *P*=NS  1f. 18/30, 60% vs 13/25, 52% vs 16/24, 67% vs 23/33, 70%, *P*=NS  1g. 23/30, 77% vs 13/20, 65% vs 30/33, 91% vs 17/24, 71%, *P*=NS 1h. 26/39, 67% vs 18/25, 72% vs 20/31, 65% vs 16/24, 67%, *P*=NS 1i. 7/22, 32% vs 10/18, 56% vs 16/28, 57% vs 12/21, 57%, *P*=NS 1j. 2/18, 11% vs 3/10, 30% vs 3/11, 27% vs 1/9, 11%, *P*=NS 1k. 5/10, 50% vs 2/4, 50% vs 3/9, 33% vs 2/9, 22%, *P*=NS  2a. 81% vs 80% vs 82% vs 80%  2b. 0.95 (1.1) vs 0.85 (1.0) vs 0.89 (1.1) vs 0.88 (1.0) 2c. 128, 95% vs 89, 81% vs 109, 92% vs 96, 87% 2d. 0.98 (0.8) vs 1.00 (2.7) vs 1.1 (2.0) vs 0.92 (1.0)  3. 1.9 (0.9) vs 2.0 (0.9) vs 2.1 (0.6) vs 2.1 (0.7) 4. 2.1 (0.7) vs 2.1 (0.8) 2.0 (0.6) vs 2.1 (0.7) | Physician intervention vs pharmacist intervention vs both interventions vs control  All prespecified with follow-up at 12 months. **1. Mean (SD) SF-36 subscale scores (N/grp: 135 vs 110 vs 118 vs 111). Higher scores better.  1a. Physical function.  1b. Role physical.  1c. Pain.  1d. General health.  1e. Vitality.  1f. Social function.  1g. Role emotional.  1h. Mental health.**   **2. Mean (SD) McMaster Asthma Quality of Life Questionnaire subscale scores (Number/group: 38 vs 31 vs 27 vs 20). Higher scores better.  2a. Overall health status.**  2b. Activity.  2c. Symptoms.  2d. Emotion.  2e. Environment.   **3. Mean (SD) McMaster Chronic Respiratory Disease Questionnaire subscale scores (Number/group: 72 vs 104 vs 91 vs 91). Higher scores better.  3a. Overall health status.**  3b. Dyspnoea.  3c. Fatigue.  3d. Emotion.  3e. Mastery.  4**. Mean (SD) number of emergency department visits.  4a. For any reason.**  4b. For reactive airways disease.  5**. Mean (SD) number of hospitalizations. 5a. For any reason.**  5b. For reactive airways disease. | All p=NS unless noted otherwise. 1a. 38 (23) vs 38 (27) vs 36 (24) vs 37 (26)  1b. 32 (40) vs 33 (40) vs 38 (41) vs 32 (40), *P<*.05 in favour of both interventions 1c. 49 (25) vs 47 (27) 48 (26) vs 44 (26) 1d. 37 (24) vs 29 (25) vs 35 (20) vs 34 (22) 1e. 37 (21) vs 39 (23) vs 36 (23) vs 36 (20) 1f. 69 (27) vs 63 (30) vs 61 (29) vs 63 (29)  1g. 65 (43) vs 60 (44) vs 59 (43) vs 60 (45) 1h. 62 (23) vs 62 (23) vs 50 (25) vs 61 (24)  2a. 4.0 (1.5) vs 4.2 (1.4) vs 4.2 (1.1) vs 3.7 (1.3) 2b. 4.5 (1.5) vs 4.6 (1.3) vs 4.4 (1.2) vs 3.9 (1.2)  2c. 4.0 (1.5) vs 4.0 (1.5) vs 4.2 (1.2) vs 3.6 (1.4) 2d. 3.8 (2.0) vs 4.3 (1.6) vs 4.4 (1.2) vs 3.6 (1.5), *P<*.05 in favour of pharmacist intervention 2e. 3.9 (1.6) vs 4.2 (1.5) vs 4.0 (1.4) vs 3.7 (1.4)   3a. 4.4 (1.2) vs 4.3 (1.3) vs 4.1 (1.1) vs 4.2 (1.1) 3b. 4.2 (1.6) vs 4.2 (1.7) vs 4.0 (1.6) vs 4.0 (1.5) 3c. 3.8 (1.3) vs 3.7 (1.5) vs 3.4 (1.2) vs 3.6 (1.2) 3d. 4.6 (1.3) vs 4.5 (1.4) vs 4.2 (1.2) vs 4.4 (1.3) 3e. 4.8 (1.4) vs 4.8 (1.5) vs 4.5 (1.4) vs 4.6 (1.4)  4a. 1.4 (1.7) vs 1.5 (2.3) vs 1.4 (2.1) vs 1.4 (1.9) 4b. 0.3 (0.7) vs 0.4 (0.8) vs 0.4 (0.8) vs 0.3 (0.8) 5a. 0.5 (1.6) vs 0.5 (1.1) vs 0.4 (1.1) vs 0.4 (0.8) 5b. 0.1 (0.5) vs 0.1 (0.5) vs 0.1 (0.5) vs 0.1 (0.3) | 0 | 0 |
| Eccles, 2002[54, 55] ^c^ | **Prespecified**  **1. Adherence to angina guideline recommendations for all patients (n=2335; n=1117 computerized system, n=1218 controls) proportion of patients 12 months before/12 months after intervention period; OR (95%CI). 1a. BP recorded.  1b. Exercise recorded or advised.  1c. Weight recorded or advised.  1d. Smoking status known. 1e. Smoking education given. 1f. 12 lead electrocardiogram recorded. 1g. Exercise electrocardiogram recorded. 1h. Hb concentration recorded. 1i. Thyroid function recorded. 1j. Cholesterol or other lipid concentrations recorded. 1k. Blood glucose or HbA1c concentrations recorded.**  2. Adherence to angina guideline recommendations for patients consulting during the intervention period (n=2276; n=1084 computerized system, n=1192 controls) proportion of patients 12 months before/12 months after intervention period; OR (95%CI). 2a. BP recorded.  2b. Exercise recorded or advised.  2c. Weight recorded or advised.  2d. Smoking status known. 2e. Smoking education given. 2f. 12 lead electrocardiogram recorded. 2g. Exercise electrocardiogram recorded. 2h. Hb concentration recorded. 2i. Thyroid function recorded. 2j. Cholesterol or other lipid concentrations recorded. 2k. Blood glucose or HbA1c concentrations recorded.  **3. Drugs prescribed for patients with angina (n=2881; n=1415 computerized system, n=1466 controls) proportion of patients 12 months before/12 months after intervention period; OR (95%CI). 3a. Short acting glyceryl trinitrate. 3b. β-blockers. 3c. Verapamil. 3d. Modified release glyceryl trinitrate. 3e. Transdermal glyceryl trinitrate. 3f. Isosorbide dinitrate (short acting and modified release). 3g. Isosorbide monomitrate (short acting and modified release). 3h. Diltiazem. 3i. Calcium channel blockers. 3j. Statins. 3k. β-blocker and dinitrate (guideline specifically recommended not using these combinations). 3l. Calcium blocker and dinitrate (guideline specifically recommended not using these combinations). 3m. Nitrate, calcium blocker and β-blocker (guideline specifically recommended not using these combinations).**  **4. Adherence to asthma guideline recommendations for all patients (n=2363; n=1200 computerized system, n=1163 controls); proportion of patients 12 months before/12 months after intervention period; OR (95%CI)). *4a. Lung function assessed. 4b. Compliance checked. 4c. Inhaler technique assessed. 4d. Asthma education, action plan, or both. 4e. Smoking status known. 4f. Smoking cessation advice or nicotine replacement therapy.**  5. Adherence to asthma guideline recommendations for patients consulting during the intervention period (n=2230; n=1129 computerized system, n=1101 controls); proportion of patients 12 months before/12 months after intervention period; OR (95%CI)). 5a. Lung function assessed. 5b. Compliance checked. 5c. Inhaler technique assessed. 5d. Asthma education, action plan, or both. 5e. Smoking status known. 5f. Smoking cessation advice or nicotine replacement therapy.  **6. Drugs prescribed for patients with asthma (n=2776; n=1391 computerized system, n=1385 controls) proportion of patients 12 months before/12 months after intervention period; OR (95%CI). 6a. Short acting β2 agonists. 6b. Inhaled corticosteroids. 6c. Long acting β2 agonists. 6d. Oral steroids. 6e. Oral bronchodilators.** | 1a. 77%/80% vs 77%/80%; 1.01 (0.74 to 1.39)  1b. 9%/10% vs 13%/13%; 0.91 (0.55 to 1.50) 1c. 23%/26% vs 24%/30%; 0.86 (0.54 to 1.35) 1d. 20%/22% vs 22%/32%; 0.68 (0.42 to 1.11) 1e. 3%/4% vs 3%/4%; 1.08 (0.86 to 1.77) 1f. 15%/14% vs 16%/14%; 1.01 (0.68 to 1.52) 1g. 4%/3% vs 4%/3%; 1.01 (0.56 to 1.80) 1h. 29%/33% vs 29%/33%; 1.01 (0.72 to 1.42) 1i. 17%/19% vs 18%/22%; 0.83 (0.62 to 1.12) 1j. 35%/43% vs 35%/47%; 0.85 (0.65 to 1.12) 1k. 20%/27% vs 22%/27%; 0.96 (0.67 to 1.39)  2a. 79%/82% vs 79%/82%; 1.95 (0.75 to 1.46 2b.9%/10% vs 13%/13%; 0.90 (0.54 to 1.46) 2c.23%/26% vs 24% vs 30%; 0.87 (0.55 to 1.37) 2d. 20%/22% vs 22%/32%; 0.68 (0.41 to 1.13) 2e. 3%/4% vs 3%/4%; 1.09 (0.66 to 1.78) 2f. Only post-intervention data: 9% vs 8%; 0.94 (0.58 to 1.53) 2g. Only post-intervention data; 2% vs 2%; 1.05 (0.56 to 1.98) 2h. Only post-intervention data: 29% vs 26%; 1.08 (0.74 to 1.56) 2i. Only post-intervention data: 16% vs 16%; 0.94 (0.67 to 1.33) 2j. Only post-intervention data: 45% vs 48%; 0.87 (0.66 to 1.14) 2k. Only post-intervention data: 28% vs 28%; 0.97 (0.67 to 1.41)  3a. 58%/57% vs 57%/55%; 1.11 (0.87 to 1.41) 3b. 47%/48% vs 49%/49%; 0.99 (0.73 to 1.33) 3c. 2%/2% vs 1%/1%; 1.02 (0.57 to 1.82) 3d. 3%/3% vs 3%/3%; 0.97 (0.50 to 1.54) 3e. 1%/1% vs 2%/2%; 1.03 (0.54 to 1.98) 3f. 5%/4% vs 6%/5%; 0.91 (0.63 to 1.31) 3g. 37%/37% vs 38%/37%; 1.11 (0.79 to 1.56) 3h. 19%/19% vs 21%/20%; 1.43 (0.87 to 2.34) 3i. 28%/27% vs 26%/25%; 1.12 (0.80 to 1.58) 3j. 29%/35% vs 30%/38%; 0.92 (0.67 to 1.25) 3k. 1%/1% vs 2%/2%; 1.24 (0.66 to 2.33) 3l. 2%/2% vs 3%/3%; 1.15 (0.68 to 1.95) 3m. 8%/7% vs 8%/8%; 0.75 (0.46 to 1.22)  4a. 43%/43% vs 42%/45%; 0.94 (0.67 to 1.33) 4b. 36%/37% vs 38%/41%; 0.82 (0.58 to 1.15) 4c. 17%/19% vs 20%/23%; 0.8 (0.5 to 1.28) 4d. 7%/5% vs 9%/7%; 0.84 (0.4 to 1.74) 4e. 24%/32% vs 26%/32%; 0.97 (0.65 to 1.45) 4f. 5%/7% vs 6%/9%; 0.75 (0.45 to 1.26)   5a. 45%/45% vs 45%/47%; 0.94 (0.66 to 1.34) 5b. 37%/39% vs 40%/43%; 0.82 (0.58 to 1.16) 5c. 18%/20% vs 21%/24%; 0.81 (0.5 to 1.28) 5d. 7%/5% vs 10%/7%; 0.81 (0.39 to 1.67) 5e. 25%/33% vs 28%/33%; 0.98 (0.66 to 1.46) 5f. 5%/8% vs 6%/9%; 0.76 (0.46 to 1.27)  6a. 82%/80% vs 84%/80%; 1.04 (0.83 to 1.31) 6b. 77%/72% vs 73%/70%; 0.95 (0.78 to 1.16) 6c. 13%/14% vs 12%/13%; 0.84 (0.59 to 1.20) 6d. 23%/23% vs 21%/21%; 1.0 (0.82 to 1.22) 6e. 7%/7% vs 9%/9%; 1.38 (0.56 to 3.39) | Prespecified **1. Change in overall quality of life (SF-36 and EQ-5D questionnaires) from 12 months before to 12 months after intervention. 2. Change in disease-specific quality of life (Seattle angina questionnaire, Newcastle asthma symptoms questionnaire, and the asthma quality of life questionnaire) from 12 months before to 12 months after intervention.**  **3. Mean (SD) number of consultations by angina patients; OR (95%CI),**  **3a. During intervention period.**  **3b. For angina.**  **4. Mean (SD) number of consultations by asthma patients; OR (95%CI),**  **4a. During intervention period.**  **4b. For asthma.** | 1. No difference between groups (data not reported) 2. No difference between groups (data not reported)  3a. 8.5 (6.4) vs 8.6 (6.2); 1.10 (0.91 to 1.11) 3b. 1.6 (2.4) vs 1.6 (2.3); 1.05 (0.83 to 1.33)  4a. 6.7 (6.3) vs 6.8 (5.8); 1.01 (0.92 to 1.11) 4b. 1.5 (2.3) vs 1.6 (2.2); 0.94 (0.81 to 1.06) | 0 | 0 |
| McCowan, 2001[56] | N = 147 vs 330 patients; 6 month follow-up **Main outcomes 1. Primary care consultations: number (proportion) of patients; OR (95% CI) 1a. Practice initiated review 1b. Issued peak flow meter 1c. Used a self-management plan  2. Number (proportion) of patients who received each assessment; OR (95% CI). (symptom outcome prespecified; rest not clearly prespecified) 2a. symptoms** 2b. Night time symptoms 2c. Symptoms on waking 2d. Symptoms on exercise 2e. Inhaler technique checked 2f. Compliance checked. 2g. Peak flow measured.  **3. Prescriptions for acute asthma exacerbations; number (proportion) of patients; OR (95% CI) (prespecified).**  **3a. Received oral corticosteroids.**  **3b. Received emergency nebulisations.** | 1a. 49 (33%) vs 139 (42%); 0.69 (0.21 to 2.21) 1b. 77 (52%) vs 158 (48%); 1.52 (0.58 to 4.01) 1c. 75 (50%) vs 173 (52%); 1.32 (0.42 to 4.16)  2a. 8 (5%) vs 44 (13%); 0.31 (0.03 to 3.32) 2b. 7 (5%) vs 52 (16%); 0.27 (0.01 to 6.98) 2c. 12 (11%) vs 60 (18%); 0.40 (0.06 to 2.78) 2d. 45 (31%) vs 133 (40%); 0.65 (0.14 to 3.16) 2e. 45 (31%) vs 133 (40%); 0.65 (0.14 to 3.16) 2f. 47 (32%) vs 155 (47%); 0.53 (0.11 to 2.50) 2g. data missing Note: rows may be offset (i.e symptoms as a header for 2b-2d rather than an item on its own. Checking with author).  3a.. 7 (5%) vs 35 (11%); 0.42 (0.14 to 1.29) 3b. 1 (1%) vs 17 (5%); 0.13 (0.01 to 0.91) | N=147 vs 330 patients; 6 month follow-up.  **1. Number (proportion) of patients with acute exacerbation of asthma; OR (95% CI) (primary)**  2. Primary care consultations: number (proportion) of patients; OR (95% CI) for patient initiated consultation  3. Number (proportion) of patients with hospital contacts for asthma; OR (95% CI) (prespecified)  3a. Admissions.  3b. Accident and emergency.  3c. Outpatients. | 1. 12 (8%) vs 57 (17%); 0.43 (0.21 to 0.85)  2. 34 (22%) vs 111 (34%); 0.59 (0.37 to 0.95)  3a. 0% vs 4 (1%); 0 (0 to 3.44) 3b. 0% vs 2 (1%); 0 (0 to 9.16) 3c. 2 (1%) vs 7 (2%); 0.64 (0.09 to 3.38) | 0 | + |
| **Dyslipidaemia** | | | | | | |
| Bertoni, 2009[57, 58] | 3-year follow-up 1. Lipid screening rates for patients (secondary)  For change from baseline to follow-up in proportion of patients; Difference; intra-class correlation  **2. Appropriate lipid management (met 1 of 7 criteria based on LDL-cholesterol level and risk strata) (primary)  For change from baseline to follow-up in proportion of patients; Difference; intra-class correlation**  3. Inappropriate prescription of lipid-lowering therapy (LLT) (secondary). For change from baseline to follow-up in proportion of patients; Difference  4. Appropriate prescription of LLT. (secondary) For change from baseline to follow-up in proportion of patients; Difference  Stratified subgroup analyses 5-7. Appropriate lipid management* of patient dyslipidaemia by Risk Category* 5. Low risk patients: baseline n=296 vs 357; follow-up n=309 vs 336 6. Intermediate low-risk or intermediate high-risk: baseline n=315 vs 281; follow-up n=253 vs 254 7. High risk patients: baseline n=231 vs 217; follow-up n=147 vs 181 For change from baseline to follow-up in proportion of patients; Difference; intraclass correlation  *Risk category defined by Framingham risk score (history and 10-year risk of CHD)  (1) Low risk (0-1 risk factor for CHD); (2) intermediate low risk (≥2 risk factors and a 10 year risk of <10%) (3) intermediate high risk (≥2 risk factors and a 10 year risk of 10% to 20% )(4) high risk (CHD risk equivalent [diabetes, CHD, stroke, or peripheral vascular disease] and/or ≥2 risk factors with a 10 year risk of >20%) | 1. +6.6 vs +10.7; -5.3; *P=*.22; 0.22  2. -1.1 vs -10.8; +9.7; *P*=<.01; 0.01  3. -2.7 vs +2.2; -4.9; *P=*.01  4. -14.0 vs. -21.2; +7.2; *P=*.37  5. -0.5 vs. -4.9; +4.4; *P=*.21; 0.01  6. +0.9 vs. -7.3; +8.2; *P=*.03; 0.01  7. -23.1 vs -26.9; +3.8; *P=*.65; 0.01 | … | … | + | … |
| Gilutz, 2009[59] | Mean 21-month follow-up **1. Appropriate initiation, up-titration, or continuation of statin therapy; % (unclear if represents patients); difference; OR (unclear if lower & upper ranges represent 95% CIs) (primary).**  2. Appropriate uptitration in patients with LDL-C≥110 mg/dL, % (unclear if represents patients - not prespecified).  **3. Rate of adequate lipoprotein monitoring: % (not clear if represents patients); OR (unclear if lower & upper ranges represent 95% CIs) (primary).** | 1. 59.1% vs 53.7%; 5.4% (2.5% drug initiation, 1.8% up-titration, and 1.1% avoiding drug cessation), *P<*.003; 1.232 (lower 1.112, upper 1.365), *P=*.001  2. 8.6% vs 7.4%, *P*=NS  3. 54.8% vs 48.7%, *P<*.001; 1.28 (lower 1.17, upper 1.41), *P<*.001 | Mean 21 month follow-up. **1. Change in LDL-C level in 52.5% of patients with initial LDL-C >120 mg/dL: Baseline/Final mean (SD), % reduction (primary). Note: data for 38.5% of patients with initial LDL <110 mg/dL and 9% with initial LDL-C 110-120 mg/dL were not reported.**  2. Proportion of patients who are live and have not had a CV rehospitalization during 1^st^ year, % (secondary). | 1. 145.5 (22.3) / 121.9 (34.2), 16.2% vs 145.8 (22.9) / 124.3 (34.6), 14.8%, *P<*.02  2. 57.1% vs 59.2%, *P<*.03 | + | + |
| Lester, 2006[60, 61] | **1. Patients with changes in statin prescriptions at 1 month (primary), n/N, %. 2. Patients with changes in statin prescriptions at 12 months (primary), n/N, %.**  3. median (IQR) time to first measured LDL-C after study initiation (not prespecified)  NOTE: the preliminary data in the 2004 paper reports 15 primary care providers and 256 pts randomized; 2006 publication only mentions 14 primary care providers and 235 patients, Author indicated that 1 physician (centre) was lost during the study, hence different numbers.  2004 also reports 1 outcome not in 2006 paper – looks like 1 month follow-up (but not explicit): Patients with repeat fasting lipid profile ordered. 12.9% vs 7.6%, *P=*.16 | 1. 18/118, 15.3% vs 2/117, 2%, *P=*.001  2. 29/118, 24.6% vs 20/117, 17.1%, *P=*.14  3. 99 (48 to 171) vs 121 (45 to 208), *P=*.48 | **1. Patients with change in LDL-C levels of all patients with LDL-C results (primary), n/N, %** 2. mean (SD) first LDL-C level after intervention (part of primary) 3. mean (SD) final LDL-C level (part of primary)  Prespecified subgroup analysis.  4. Patients with LDL-C level > 130mg/dL at baseline, n/N, %.  5. Of patients with LDL-C>130 mg/dL at baseline, mean (SD) first LDL after intervention. 6. Of patients with LDL-C >130 mg/dL at baseline, mean (SD) final LDL-C level. | 1. 81/118, 68.6% vs 82/117, 82%, *P=*.8 2. 111.7 (30.2) vs 118.1 ( 32.1), *P=*.2 3. 106.8 (26.8) vs 111.5 (30.0), *P=*.3  4. 41/118, 34.7% vs 39/117, 33.3%, *P=*.9 5. 119 (32.1) vs 138 (35.6), *P=*.04 6. 111.4 (29.3) vs 128.3 (35.7), *P=*.055 | + | 0 |
| Cobos, 2005[62] | Mean follow-up 12.2 vs 11.2 months  All secondary  **1. Mean number of scheduled physician visits.**  **2. Mean number of assessments.**  **2a. Lipid assessments.**  **2b. Aspartate amintransferase/ alanine aminotransferase measurements.**  **2c. Creatine kinase determinations.**  **3. Number (%) patients treated with lipid-lowering drugs; OR (95% CI).**  **3a. Patients with CHD.**  **3b. High-risk patients without CHD.**  **3c. Low-risk patients without CHD.** | 1. 1.8 vs 1.9, *P=*.311  2a.  1.83 vs 1.87, *P=*.298 2b. 1.41 vs 1.31, *P=*.033 2c. 0.54 vs 0.24, *P=*.053  3a. 102 (92.7%) vs 125 (85.0%); 2.54 (0.92 to 6.98) 3b. 201 (70.5%) vs 260 (76.9%); 0.69 (0.44 to 1.06) 3c. 124 (19.0%) vs 292 (44.2%); 0.25 (0.16 to 0.41) | Mean follow-up 12.2 vs 11.2 months.  **Primary outcome & analysis:**  **1. n/N (%) patients with successful management* in ITT analysis; difference (95% lower confidence limit); OR (95% CI).**  Secondary  2. Mean number of physician visits.  1b. Unscheduled and related to drug treatment or hypercholesterolemia.  Subgroup analyses - not prespecified.  3. Proportion of patients with successful management (ITT: 1046 vs 1145 patients).  3a. Patients with CHD and no previous lipid-lowering drug treatment.  3b. Patients with CHD and previous lipid-lowering drug treatment.  3c. High-risk patients without CHD and no previous lipid-lowering drug treatment.  3d. High-risk patients without CHD and previous lipid-lowering drug treatment.  3e. Low-risk patients without CHD and no previous lipid-lowering drug treatment.  3f. Low-risk patients without CHD and previous lipid-lowering drug treatment.  Secondary outcomes  4. Mean final lipid values (mg/dL) ; difference (95% CI) (ITT: 1046 vs 1145 patients).  4a. Total cholesterol.  4b. LDL-C.  4c. HDL-C.  4d. Tryglycerides.  *Management success:  a. If CV risk ≥20% over 10 yrs, success = LDL-C < 115mg/dL at study end for patients with CHD or < 130mg/dL for those without CHD.  b. If CV risk <20% over 10 years, success = CVR still <20% at study end. | 1. 565/1046 (54.02%) vs 578/1145 (50.48%); 3.53% (-4.97)*; 1.02 (0.58 to 1.77)  2. 0.03 vs 0.03, *P=*.855 3a. 23.69% vs 23.39%, *P*=NS 3b. 22.26% vs 21.98%, *P*=NS 3c. 21.53% vs 21.25%, *P*=NS 3d. 20.20% vs 19.94%, *P*=NS 3e. 73.68% vs 73.36% , *P*=NS 3f. 72.09% vs 71.76%, *P*=NS Note: No significant interactions for group by CV risk level or group by previous lipid-lowering drug treatment.  4a. 233.8 vs 231.0; 2.8 (-1.7, -7.3); *P=*.218 4b. 149.2 vs 146.5; 2.7 (-1.7, -7.1); *P=*.227 4c. 58.0 vs 56.3; 1.6 (-0.6, -3.6); *P=*.142 4d. 136.6 vs 135.2; 1.4 (-8.3, -11.2); *P=*.766 | 0 | 0 |
| **Cardiac Care** | | | | | | |
| Goud, 2009[63, 64] | **Main outcome**  **1. Concordance with guideline recommendations over 6 months: Number (%) of patients; crude difference, adjusted* difference (95% CI), intra-cluster correlation; Data not available, number of patients (%).  1a. Exercise training.  1b. Education therapy. 1c. Relaxation therapy.  1d. Lifestyle change therapy.**  2. Number (%) of patients undertreated. (Prespecified) 2a. Exercise training.  2b. Education therapy. 2c. Relaxation therapy.  2d. Lifestyle change therapy.  3. Number (%) of patients overtreated. (Prespecified) 3a. Exercise training.  3b. Education therapy. 3c. Relaxation therapy.  3d. Lifestyle change therapy.  * Adjusted for age, sex, diagnosis, weekly centre volume of new patients, and centre specialised or part of an academic hospital. Note: 5 of 15 control centres discontinued participation during trial; and data from 4 intervention and 1 additional control centre were excluded for poor data quality or missing data. | 1a. 1508/1629 (92.6%) vs 933/1102 (84.7%); 7.9%, 3.5% (0.1 to 5.2); 0.086, 56 (2.1%) 1b. 1411/1610 (87.6%) vs 709/1110 (63.9%), 23.7%, 23.7% (15.5 to 29.4); .0.187, 67 (2.4%) 1c. 959/1610 (59.6%) vs 373/1094 (34.1%); 25.5%, 41.6% (25.2 to 51.3); 0.479, 83 (3.0%) 1d. 924/1610 (57.4%) vs 601/1110 (54.1%); 3.3%, 7.1% (-2.9 to 18.3); 0.110, 67 (2.4%)  2a. 79/1629 (4.8%) vs 100/1102 (9.1%). 2b. 156/1610 (9.7%) vs 334/1110 (30.1%). 2c. 634/1610 (39.4%) vs 676/1094 (61.8%). 2d. 672/1610 (41.7%) vs 458/1110 (41.3%).  3a. 42/1629 (2.6%) vs 69/1102 (6.3%) 3b. 43/1610 (2.7%) vs 67/1110 (6.0%) 3c. 17/1610 (1.1%) vs 45/1094 (4.1%) 3d. 14/1610 (0.9%) vs 51/1110 (4.6%) | … | … | + | … |
| Feldman, 2005[65, 66] | Not prespecified as an outcome; data obtained from patient chart abstraction. **Augmented intervention (n=118 nurses) vs usual care (n=122);** basic intervention (n=114) vs usual care.  1) Proportion of nurses recording the following assessments over 45 days: %, p-value.  1a. Comprehensive heart failure assessment (weight, shortness of breath, and oedema) at all visits for all assigned patients.  1b. Current diet (≥ 1 time for each assigned patient).  1c. Medication knowledge (≥ 1 time for each assigned patient).  1d. Adherence to medication (≥ 1 time for each assigned patient).  1e. Medication side effects (≥ 1 time for each assigned patient).   2. Proportion of nurses instructing patients (or caregivers) on the following over 45 days: %, p-value.  2a. Heart failure signs and symptoms (shortness of breath, fluid weight gain, or fatigue, or general signs and symptoms ≥ 1 time for each assigned patient).  2b. Heart failure symptom: shortness of breath (≥ 1 time for each assigned patient).  2c. Heart failure symptom: fluid weight gain (≥ 1 time for each assigned patient).  2d. Heart failure symptom: fatigue (≥ 1 time for each assigned patient).  2e. Weighing self (≥ 1 time for each assigned patient).  2f. Managing fluid weight gain (≥ 1 time for each assigned patient).  2g. Low salt diet (≥ 1 time for each assigned patient).  2h. Medication management (≥ 1 time for each assigned patient).  2i. Methods to improve medication adherence (≥ 1 time for each assigned patient).  2j. When to contact a physician (≥ 1 time for each assigned patient).  2k. Provided Heart failure self-care guide (≥ 1 time for each assigned patient).  **3. Proportion of patients with the following self-management indicators: %, p-value  (Prespecified). 3a. Patient skips medicine.  3b. Patient is sure about when to take heart failure medication.  3c. Patient recognised own heart failure medicines: None/≤50%/>50%.  3d. Patient salts food.  3e. Patient’s weighing behaviour: no scale/weighs self < daily/weigh self daily.**  **4. Service use measures (prespecified). 4a. (Mean*) number of home care-related visits.  4b. Proportion for any outpatient doctor visit (appears to be proportion of patients but article is not explicit).  4c. (Mean*) number of outpatient doctors’ visits.**  5. Cost per patient to produce a 5% improvement in Kansas City Cardiomyopathy Questionnaire summary score: Augmented/Basic interventions  (not clearly prespecified). For home-care related costs.   6. Cost per patient to produce a 5% improvement in EuroQoL-5 Dimensions score: Augmented*/Basic interventions (not clearly prespecified).  For home-care related costs.  * Appears to be mean but the article is not explicit. | 1a. 23.9% vs 3.7%, *P<*.001; 13.8% vs 3.7%, *P=*.006 1b. 48.7% vs 27.6%, *P=*.001; 38.2% vs 27.6%, *P=*.076 1c. 34.4% vs 24.8%, *P=*.109; 31.1% vs 24.8%, *P=*.285 1d. 59.6% vs 48.2%, *P=*.077; 62.7% vs 48.2%, *P=*.024 1e. 23.6% vs 12.7%, *P=*.03; 15.3% vs 12.7%, *P=*.558  2a. 59.5% vs 42.1%, *P=*.007; 53.9% vs 42.1%, *P=*.07,  2b. 28.9% vs 18.1%, *P=*.053; 31.1% vs 18.1%, *P=*.021 2c. 39.7% vs 20.6%, *P=*.001; 29.9% vs 20.6%, *P=*.097 2d. 15.9% vs 11.8%, *P=*.353; 10.5% vs 11.8%, *P=*.752 2e. 48.7% vs 16.0%, *P<*.001; 37.2% vs 16.0%, *P<*.001 2f. 11.9% vs 5.7%, *P=*.116; 8.0% vs 5.7%, *P=*.505 2g. 49.6% vs 22.7%, *P<*.001; 40.4% vs 22.7%, *P=*.003 2h. 59.7% vs 51.2%, *P=*.195; 57.0% vs 51.2%, *P=*.385 2i. 18.0% vs 15.0%, *P=*.532; 26.5% vs 15.0%, *P=*.03 2j. 42.8% vs 27.3%, *P=*.014; 36.2% vs 27.3%, *P=*.147 2k. 46.2% vs 10.5%, *P<*.001; 17.6% vs 10.5%, *P=*.113  3a. 25.4% vs 27.6%, *P=*.604; 27.7% vs 27.6%, *P<*.99 3b. 69.6% vs 67.4%, *P=*.613; 70.3% vs 67.4%, *P=*.494 3c. 34.3%/30.6%/35.0% vs 43.9%/29.8%/26.3%, *P=*.023; 31.1%/30.5%/38.4% vs 43.9%/29.8%/26.3%, *P=*.002 3d. 23.3% vs 30.7%, *P=*.095; 27.6% vs 30.7%, *P=*.490 3e. 27.9%/44.7%/27.4% vs 34.6%/44.0%/21.4%, *P=*.082; 38.3%/43.0%/18.7% vs 34.6%/44.0%/21.4%, *P=*.352  4a. 44.1 vs 35.2, *P=*.053; 43.6 vs 35.2, *P=*.048 4b. 85.1% vs 82.2%, *P=*.404; 83.7% vs 82.2%, *P=*.639 4c. 2.62 vs 2.85, *P=*.546; 2.98 vs 2.85, *P=*.771  5. $235 / $183  6. NA*/$116 *Augmented intervention not effective for improving this outcome | Patients included/evaluated: Augmented (404/202), Basic (390/199), Usual Care (448/227).  All prespecified at 45 days after admission **(augmented intervention vs usual care;** basic intervention vs usual care): **1. Kansas City Cardiomyopathy Questionnaire mean score (score range 0-100, higher scores = better outcome), p-value.  1a. Summary score.**  1b. (Mean*) physical limitation score.  1c. (Mean*) symptom score.  1d. Proportion of patients with quality of life scores ≥ 50.  1e. Proportion of patients with social limitation scores ≥ 50.  1f. Proportion of patients with self efficacy scores ≥ 50.  2**. (Mean*) EuroQoL-5 Dimensions scale score.  3. Proportion of patients with Geriatric Depression Scale score ≥ 6 (high scores = depression).**   Notes: %’s estimated from regression analyses.  **4. Service use measures (prespecified). 4a. Proportion for any hospitalization (appears to be proportion of patients but article is not explicit)..  4b. (Mean*) number of inpatient nights.  4c. Proportion for emergency department visits (appears to be proportion of patients but article is not explicit)..  4d.( Mean*) number of emergency department visits.**  Notes: Data estimated from regression analyses.  5. Cost per patient to produce a 5% improvement in Kansas City Cardiomyopathy Questionnaire summary score: Augmented/Basic interventions  (not clearly prespecified). For overall costs.  6. Cost per patient to produce a 5% improvement in EuroQoL-5 Dimensions score: Augmented/Basic interventions (not clearly prespecified).  For overall costs.  *Appears to be mean but the article is not explicit. | 1a. 45.6 vs 40.4, *P=*.048; 46.6 vs 40.4, *P=*.013  1b. 43.0 vs 37.8, *P=*.231; 42.5 vs 37.8, *P=*.333 1c. 53.6 vs 48.6, *P=*.277; 55.6 vs 48.6, *P=*.091 1d. 53.3% vs 44.6%, *P=*.042; 48% vs 44.6%, *P=*.407 1e. 35.2% vs 27.8%, *P=*.064; 34.8% vs 27.8%, *P=*.09 1f. 86.3% vs 85.8%, *P=*.88; 86.8% vs 85.8%, *P=*.756 2. 40.2 vs 39.3, *P=*.777; 48.9 vs 39.3, *P=*.003 3. 36.9% vs 36.3%, *P=*.888; 37.4% vs 36.3%, *P=*.802.  4a. 24.2% vs 25%, *P=*.839; 30.3% vs 25%, *P=*.209 4b. 2.33 vs 1.8, *P=*.383; 1.97 vs 1.8, *P=*.729 4c. 32.1% vs 28.8%, *P=*.459; 28.2% vs 28.8%, *P=*.882 4d. 0.53 vs 0.4, *P=*.12; 0.44 vs 0.4, *P=*.573  5. $513 / $246  6. NA*/$181 *Augmented intervention not effective for improving this outcome | 0 | 0 |
| Tierney, 2003[67] | Primary outcome (Physician intervention vs pharmacist intervention vs both intervention vs control) **1. Adherence with care suggestions over 12 months. 1a. All cardiac care suggestions.** 1b. Start or increase an ACE-I. 1c. Pneumococcal vaccination. 1d. Start or increase a β-blocker. 1e. Start low-dose aspirin. 1f. Start or increase a diuretic. 1g. Start or increase a long-acting nitrate. 1h. Start an antihyperlipidemic drug. 1i. Start or increase a calcium blocker.  2. Patient satisfaction with physicians over 12 months (secondary). 3. Patient satisfaction with pharmacist over 12 months (secondary).  4. Medication compliance over 12 months (secondary). | 1a. n/N suggestions (%). 152/648 (23%) vs 125/535 (23%) vs 134/514 (23%) vs 130/589 (22%), *P*>.2 1b-1i. n/N patients (%) 1b. 41/109 (38%) vs 40/92 (44%) vs 39/94 (42%) vs 39/107 (36%), *P*>.2 1c. 10/104 (10%) vs 7/82 (9%) vs 7/87 (8%) vs 1/82 (1%), *P=*.09 1d. 15/96 (16%) vs 11/76 (14%) vs 18/91 (20%) vs 10/83 (12%), *P*>.2 1e. 18/74 (24%) vs 17/72 (24%) vs 13/68 (19%) vs 23/81 (28%), *P*>.2 1f. 17/71 (24%) vs 11/53 (21%) vs 13/62 (21%) vs 20/73 (27%), *P*>.2 1g. 6/30 (20%) vs 8/34 (24%) vs 8/44 (18%) vs 3/25 (12%), *P*>.2 1h. 7/22 (32%) vs 5/15 (33%) vs 11/22 (50%) vs 8/22 (36%), *P*>.2 1i. 7/21 (33%) vs 5/13 (39%) vs 6/23 (26%) vs 10/17 (59%), *P*>.2  2. Data not reported, *P*>.5 3. Data not reported, *P*>.4  4. Data not reported, *P*>.69 | Physician intervention vs pharmacist intervention vs both intervention vs control  **1. Mean (SD) quality of life (score SF-36) at 12 mo (primary).  1a. Physical function 1b. Role physical 1c. Pain 1d. General health 1e. Vitality 1f. Social function 1g. Role emotional 1h. Mental health**  **2. Mean (SD) quality of life (Chronic heart disease questionnaire subscale scores) at 12 months (primary). 2a. Overall health status** 2b. Dyspnoea 2c. Fatigue 2d. Emotion  3. Mean (SD) number of emergency department visits over 12 months (secondary). 3a. All. 3b. Heart disease specific.  4. Mean (SD) number of hospitalizations over 12 months (secondary). 4a. All. 4b. Heart disease specific.  5. Mortality over 12 months (not prespecified). | 1a. 36 (27) vs 38 (26) vs 39 (27) vs 42 (26), *P*=NS 1b. 35 (40) vs 37 (41) vs 40 (42) vs 43 (42), *P*=NS 1c. 47 (28) vs 53 (29) vs 52 (27) vs 53 (28), *P*=NS 1d. 38 (22) vs 41 (24) vs 39 (22) vs 42 (24), *P*=NS 1e. 40 (23) vs 40 (25) vs 44 (24) vs 44 (25), *P*=NS 1f. 65 (30) vs 66 (31) vs 64 (32) vs 69 (28), *P*=NS 1g. 61 (46) vs 64 (44) vs 71 (43) vs 61 (44), *P*=NS 1h. 64 (22) vs 64 (23) vs 65 (24) vs 63 (25), *P*=NS 2a. 4.5 (1.2) vs 4.6 (1.2) vs 4.6 (1.3) vs 4.6 (1.2), *P*=NS 2b. 5.0 (1.5) vs 5.3 (1.5) vs 5.2 (1.6) vs 5.2 (1.4), *P*=NS 2c. 3.8 (1.4) vs 3.8 (1.5) vs 4.0 (1.5) vs 4.0 (1.3), *P*=NS 2d. 4.5 (1.3) vs 4.6 (1.4) vs 4.7 (1.4) vs 4.6 (1.4), *P*=NS 3a. 1.1 (1.9) vs 1.1 (1.8) vs 1.1 (1.4) vs 1.0 (1.7), *P*=NS 3b. 0.2 (0.4) vs 0.2 (0.6) vs 0.1 (0.4) vs 0.2 (0.5), *P*=NS  4a. 0.4 (1.0) vs 0.5 (1.0) vs 0.5 (1.1) vs 0.5 (1.1), *P*=NS 4b. 0.2 (0.6) vs 0.2 (0.7) vs 0.2 (0.6) vs 0.2 (0.5), *P*=NS  5. Data not reported (2% overall), *P*>.9 | 0 | 0 |
| Eccles, 2002[54, 55] ^c^ | **Prespecified**  **1. Adherence to angina guideline recommendations for all patients (n=2335; n=1117 computerized system, n=1218 controls) proportion of patients 12 months before/12 months after intervention period; OR (95%CI). 1a. BP recorded.  1b. Exercise recorded or advised.  1c. Weight recorded or advised.  1d. Smoking status known. 1e. Smoking education given. 1f. 12 lead electrocardiogram recorded. 1g. Exercise electrocardiogram recorded. 1h. Hb concentration recorded. 1i. Thyroid function recorded. 1j. Cholesterol or other lipid concentrations recorded. 1k. Blood glucose or HbA1c concentrations recorded.**  2. Adherence to angina guideline recommendations for patients consulting during the intervention period (n=2276; n=1084 computerized system, n=1192 controls) proportion of patients 12 months before/12 months after intervention period; OR (95%CI). 2a. BP recorded.  2b. Exercise recorded or advised.  2c. Weight recorded or advised.  2d. Smoking status known. 2e. Smoking education given. 2f. 12 lead electrocardiogram recorded. 2g. Exercise electrocardiogram recorded. 2h. Hb concentration recorded. 2i. Thyroid function recorded. 2j. Cholesterol or other lipid concentrations recorded. 2k. Blood glucose or HbA1c concentrations recorded.  **3. Drugs prescribed for patients with angina (n=2881; n=1415 computerized system, n=1466 controls) proportion of patients 12 months before/12 months after intervention period; OR (95%CI). 3a. Short acting glyceryl trinitrate. 3b. β-blockers. 3c. Verapamil. 3d. Modified release glyceryl trinitrate. 3e. Transdermal glyceryl trinitrate. 3f. Isosorbide dinitrate (short acting and modified release). 3g. Isosorbide monomitrate (short acting and modified release). 3h. Diltiazem. 3i. Calcium channel blockers. 3j. Statins. 3k. β-blocker and dinitrate (guideline specifically recommended not using these combinations). 3l. Calcium blocker and dinitrate (guideline specifically recommended not using these combinations). 3m. Nitrate, calcium blocker and β-blocker (guideline specifically recommended not using these combinations).  4. Adherence to asthma guideline recommendations for all patients (n=2363; n=1200 computerized system, n=1163 controls); proportion of patients 12 months before/12 months after intervention period; OR (95%CI)). *4a. Lung function assessed. 4b. Compliance checked. 4c. Inhaler technique assessed. 4d. Asthma education, action plan, or both. 4e. Smoking status known. 4f. Smoking cessation advice or nicotine replacement therapy.**  5. Adherence to asthma guideline recommendations for patients consulting during the intervention period (n=2230; n=1129 computerized system, n=1101 controls); proportion of patients 12 months before/12 months after intervention period; OR (95%CI)). 5a. Lung function assessed. 5b. Compliance checked. 5c. Inhaler technique assessed. 5d. Asthma education, action plan, or both. 5e. Smoking status known. 5f. Smoking cessation advice or nicotine replacement therapy.  **6. Drugs prescribed for patients with asthma (n=2776; n=1391 computerized system, n=1385 controls) proportion of patients 12 months before/12 months after intervention period; OR (95%CI). 6a. Short acting β2 agonists. 6b. Inhaled corticosteroids. 6c. Long acting β2 agonists. 6d. Oral steroids. 6e. Oral bronchodilators.** | 1a. 77%/80% vs 77%/80%; 1.01 (0.74 to 1.39)  1b. 9%/10% vs 13%/13%; 0.91 (0.55 to 1.50) 1c. 23%/26% vs 24%/30%; 0.86 (0.54 to 1.35) 1d. 20%/22% vs 22%/32%; 0.68 (0.42 to 1.11) 1e. 3%/4% vs 3%/4%; 1.08 (0.86 to 1.77) 1f. 15%/14% vs 16%/14%; 1.01 (0.68 to 1.52) 1g. 4%/3% vs 4%/3%; 1.01 (0.56 to 1.80) 1h. 29%/33% vs 29%/33%; 1.01 (0.72 to 1.42) 1i. 17%/19% vs 18%/22%; 0.83 (0.62 to 1.12) 1j. 35%/43% vs 35%/47%; 0.85 (0.65 to 1.12) 1k. 20%/27% vs 22%/27%; 0.96 (0.67 to 1.39)  2a. 79%/82% vs 79%/82%; 1.95 (0.75 to 1.46 2b.9%/10% vs 13%/13%; 0.90 (0.54 to 1.46) 2c.23%/26% vs 24% vs 30%; 0.87 (0.55 to 1.37) 2d. 20%/22% vs 22%/32%; 0.68 (0.41 to 1.13) 2e. 3%/4% vs 3%/4%; 1.09 (0.66 to 1.78) 2f. Only post-intervention data: 9% vs 8%; 0.94 (0.58 to 1.53) 2g. Only post-intervention data; 2% vs 2%; 1.05 (0.56 to 1.98) 2h. Only post-intervention data: 29% vs 26%; 1.08 (0.74 to 1.56) 2i. Only post-intervention data: 16% vs 16%; 0.94 (0.67 to 1.33) 2j. Only post-intervention data: 45% vs 48%; 0.87 (0.66 to 1.14) 2k. Only post-intervention data: 28% vs 28%; 0.97 (0.67 to 1.41)  3a. 58%/57% vs 57%/55%; 1.11 (0.87 to 1.41) 3b. 47%/48% vs 49%/49%; 0.99 (0.73 to 1.33) 3c. 2%/2% vs 1%/1%; 1.02 (0.57 to 1.82) 3d. 3%/3% vs 3%/3%; 0.97 (0.50 to 1.54) 3e. 1%/1% vs 2%/2%; 1.03 (0.54 to 1.98) 3f. 5%/4% vs 6%/5%; 0.91 (0.63 to 1.31) 3g. 37%/37% vs 38%/37%; 1.11 (0.79 to 1.56) 3h. 19%/19% vs 21%/20%; 1.43 (0.87 to 2.34) 3i. 28%/27% vs 26%/25%; 1.12 (0.80 to 1.58) 3j. 29%/35% vs 30%/38%; 0.92 (0.67 to 1.25) 3k. 1%/1% vs 2%/2%; 1.24 (0.66 to 2.33) 3l. 2%/2% vs 3%/3%; 1.15 (0.68 to 1.95) 3m. 8%/7% vs 8%/8%; 0.75 (0.46 to 1.22)  4a. 43%/43% vs 42%/45%; 0.94 (0.67 to 1.33) 4b. 36%/37% vs 38%/41%; 0.82 (0.58 to 1.15) 4c. 17%/19% vs 20%/23%; 0.8 (0.5 to 1.28) 4d. 7%/5% vs 9%/7%; 0.84 (0.4 to 1.74) 4e. 24%/32% vs 26%/32%; 0.97 (0.65 to 1.45) 4f. 5%/7% vs 6%/9%; 0.75 (0.45 to 1.26)  5a. 45%/45% vs 45%/47%; 0.94 (0.66 to 1.34) 5b. 37%/39% vs 40%/43%; 0.82 (0.58 to 1.16) 5c. 18%/20% vs 21%/24%; 0.81 (0.5 to 1.28) 5d. 7%/5% vs 10%/7%; 0.81 (0.39 to 1.67) 5e. 25%/33% vs 28%/33%; 0.98 (0.66 to 1.46) 5f. 5%/8% vs 6%/9%; 0.76 (0.46 to 1.27)  6a. 82%/80% vs 84%/80%; 1.04 (0.83 to 1.31) 6b. 77%/72% vs 73%/70%; 0.95 (0.78 to 1.16) 6c. 13%/14% vs 12%/13%; 0.84 (0.59 to 1.20) 6d. 23%/23% vs 21%/21%; 1.0 (0.82 to 1.22) 6e. 7%/7% vs 9%/9%; 1.38 (0.56 to 3.39) | Prespecified **1. Change in overall quality of life (SF-36 and EQ-5D questionnaires) from 12 months before to 12 months after intervention. 2. Change in disease-specific quality of life (Seattle angina questionnaire, Newcastle asthma symptoms questionnaire, and the asthma quality of life questionnaire) from 12 months before to 12 months after intervention.**  **3. Mean (SD) number of consultations by angina patients; OR (95%CI),**  **3a. During intervention period.**  **3b. For angina.**  **4. Mean (SD) number of consultations by asthma patients; OR (95%CI),**  **4a. During intervention period.**  **4b. For asthma.** | 1. No difference between groups (data not reported) 2. No difference between groups (data not reported)  3a. 8.5 (6.4) vs 8.6 (6.2); 1.10 (0.91 to 1.11) 3b. 1.6 (2.4) vs 1.6 (2.3); 1.05 (0.83 to 1.33)  4a. 6.7 (6.3) vs 6.8 (5.8); 1.01 (0.92 to 1.11) 4b. 1.5 (2.3) vs 1.6 (2.2); 0.94 (0.81 to 1.06) | 0 | 0 |
| **Other** | | | | | | |
| Lee, 2009[68, 69] | **(Outcomes not prespecified) 1. n (%) of encounters with obesity-related diagnosis  2. n (%) of obesity -related diagnosis not screened and entered in CCDSS**  3. of obesity-related diagnoses not screened and entered by nurse 3a. n(%) with correct diagnosis based on Body Mass Index (BMI) 3b. n(%) wrong diagnosis based on BMI 3c. n (%) height and/or weight not entered  **4. n (%) encounters with missing obesity-related diagnosis (denominators are number of encounters including height and weight)** | 1. 91/807 (11.3%) vs 10/997 (1.0%), *P<*.05  2. 12/91 (13.2%) vs 10/10 (100%), *P=*.211  3a.3/12 (25%) vs 6/10 (60%), *P=*.192 3b.1/12 (8.3) vs 1/10 (10%), *P*>.99 3c.8/12 (66.7%) vs 3/10 (30%), *P=*.198  4. 51/208 (24.5%) vs 440/662 (66.5%), *P<*.05 | … | … | + | … |
| Locatelli, 2009[70] | **1. Iron usage at 4 week follow-up (secondary) 1a. n (%) patients receiving iron**  1b. % patients administered intravenous iron  1c. % patients administered oral iron  1d. % patients administered iv and oral iron 1e. % patients not given iron   **2. Erythropoetic therapy (ESA) usage at 4 week follow-up (secondary) 2a. % patients receiving ESA**  2b % patients administered intravenous ESA  2c. % patients administered subcutaneous ESA  **2d. Mean weekly combined intravenous dose 2e. Mean weekly combined subcutaneous dose**  **3. Number (%) of patients whose treatment followed guidelines at both study visits (secondary)** | 1a. 182/289 (63%) vs 142/258 (55%) 1b. 52% vs 49%, NS 1c. 8% vs 5%, NS  1d. 3% vs 1%, NS 1e. 37% vs 45%  2a. 96% vs 94% 2b. 46% vs 43% 2c. 54% vs 57% 2d. 8398 (n=127) vs 7431 (n=105) 2e. 8000 (n=147) vs 6406 (n=138)   3. 128 (21%) vs 134 (22%) | **1. Number (%) of patients achieving haematological targets: baseline / 6-8 month follow-up (N=321 vs 278)  1a. Hb >11 g/dL (primary)** 1b. Hb 11-12 g/dL 1c. Hb >13 g/dL **1d. Serrum ferritin >100 ng/mL (primary) 1e hypochromic red cell count (HRC) <10% or transferrin saturation TSAT >20% (primary)**  **2. Mean (SD) Hb (g/dl): baseline / 6-8 month follow-up (p-value for comparison of follow-up values between groups) For all patients (N=321 vs 278)** | 1a. 157 (49%) / 193 (67%) vs 140 (50%) / 181 (70%), *P*=not applicable (NA) 1b. 91 (28%) / 88 (31%) vs 58 (21%) / 82 (32%), *P*=NA 1c. 17 (5%) / 37 (13%) vs 29 (10%) / 35 (14%), *P*=NA 1d. 255 (84%) / 253 (90%) vs 221 (85%) / 237 (93%), *P=*.359 1e. 206 (79%) / 253 (86%) vs 222 (86%) / 227 (85%), *P=*.812  2. 11.0 (1.3) / 11.6 (1.3) vs 11.2 (1.4) / 11.7 (1.3), *P=*.134 | … | 0 |
| Javitt, 2008[71] | Not prespecified.  1. Resolution rate for problems identified by care considerations over 1 year: %, difference (% improvement).  **1a. Add a drug (n=601 total).**  **1b. Do a test (n=1354 total)**  **1c. Stop a drug (n=592 total).**  2. Resolution rate for 311 patients with a recommendation to use an ACE-I (based on Heart Outcomes Prevention Evaluation trial; n=155 vs 156) over 1 year.  Note: Number of care considerations issued differed between groups: 1299 vs 1519. | 1a. 26.6% vs 18%, 8.6% (48%), *P*≤.05 1b. 36.8% vs 31%, 5.8% (19%), *P*≤.05 1c. 28% vs 34%, -6% (-18%), *P*=NS  2. 27% vs 14% | ... | … | + | … |
| Verstappen, 2007[72] | … | … | **1. number (%) of patients in remission for ≥ 3 months**  1a. in first year **1b. in first two years (primary)** 2. area under the curve (IQR) standardised to time (lower = better outcome for CCDSS) (secondary) 2a. morning stiffness 2b. erythrocyte sedimentation rate 2c. tender joint count 2d. swollen joint count 2e. VAS general well-being 2f. VAS pain 2g. functional disability 3. Number (%) of patients meeting modified American College of Radiology 50 criteria (pre-specified) 3a. at one year 3b. at two years  4. mean (95% CI) time (months) until the first period of remission (not pre-specified) 5. duration (CI) (months) of all periods of remission together (not pre-specified)  6. median (IQR)/mean (95%CI) annual radiographic progression over 2 years (units/year) (not pre-specified)  Adverse events were evaluated at each visit according to a predefined protocol.  7. percentage of patients with adverse event) 8. number of adverse events/number of protocol visits after methotrexate initiated   3. percentage of total number of adverse events 9a. gastrointestinal 9b. mucocutaneous reaction 9c. neurological disorders 9d. renal events 9e. liver toxicity 9f. haematological abnormalities 9g. pulmonary symptoms 9h. post-dosing reactions of methotrexate 9i. other  4. mean (SD) change from baseline after 1 year (prespecified) CCDSS vs Control, Mean (95%CI) difference;  10a. erythrocyte sedimentation rate, mm/h1st – all patients 10b. erythrocyte sedimentation rate, mm/hlst - completers 10c. Morning stiffness, min. - all patients  10d. Morning stiffness, min. - completers  10e. Number of swollen joints – all patients  10f. Number of swollen joints - completers  10g. Number of tender joints - all patients 10h. Number of tender joints - completers  10i. VAS general well-being, mm – all patients 10j. VAS general well-being, mm - completers  10k VAS pain, mm - all patients  10l. VAS pain, mm - completers 10m. Functional disability, Health Assessment Questionnaire - all patients  10n. Functional disability, Health Assessment Questionnaire - completers   11. mean (SD) change from baseline after 2 years (prespecified) CCDSS vs Control, Mean (95%CI) difference  11a. erythrocyte sedimentation rate, mm/h1st – all patients 11b. erythrocyte sedimentation rate, mm/hlst - completers 11c. Morning stiffness, min. - all patients  11d. Morning stiffness, min. - completers  11e. Number of swollen joints – all patients  11f. Number of swollen joints - completers  11g. Number of tender joints - all patients 11h. Number of tender joints - completers  11i. VAS general well-being, mm – all patients 11j. VAS general well-being, mm - completers  11k VAS pain, mm - all patients  11l. VAS pain, mm - completers 11m. Functional disability, Health Assessment Questionnaire - all patients  11n. Functional disability, Health Assessment Questionnaire - completers | 1a. 53 (35%) vs 21 (14%), *P<*.001 1b. 76 (50%) vs 55 (37%), *P=*.029 2a. 17.0 (7.5 to 41.2) vs 23.7 (12.3 to 56.7), *P=*.009 2b. 17.7 (10.2 to 27.6) vs 21.6 (13.0 to 33.6), *P=*.007 2c. 3.6 (1.9 to 6.0) vs 5.5 (2.8 to 9.2), *P<*.001 2d. 2.7 (1.5 to 5.2) vs 4.7 (2.8 to 7.6), *P<*.001 2e. 19.0 (11.5 to 35.4) vs 31.2 (16.2 vs 44.6), *P<*.001 2f. 12.0 (5.0 to 24.3) vs 19.0 (9.5 to 34.1), *P=*.001 2g. 0.64 (0.3 to 1.3) vs 0.80 (0.3 to 1.2), *P=*.8 3a. 87 (58%) vs 64 (43%), *P=*.018 3b. 69 (46%) vs 67 (45%), *P*>.99 4. 10.4 (9.1 to 11.7) vs 14.3 (12.6 to 16.1), *P<*.001 5. 11.6 (10.1 to 13.1) vs 9.1 (7.6 to 10.6), *P=*.025 6. 0 (0 to 2.0) / 1.9 (1.0 to 2.7) vs 0 (0 to 2.5) / 2.1 (1.3 to 2.8), *P=*.9  7. 94% vs 87%   8. 2378/3190 vs 873/1132  9a. 24.6% vs 25.2% 9b. 14.8% vs 18.2% 9c. 18.8% vs 18.8% 9d. 2.4% vs 2.8% 9e. 23.2% vs 18.6% 9f. 7.1% vs 4.2% 9g. 2.0% vs 5.3% 9h. 1.8% vs 2.1% 9i. 5.2% vs 4.8%  10a. -18 (27) vs -15 (24), -3 (-9 to 2) 10b. -24 (27) vs -16 (24), -7 (-15 to -0.4) 10c. -63 (61) vs -56 (59), -7 (-21 to 6) 10d. -73 (56) vs -64 (57), -9 (-25 to 7) 10e. -11 (8) vs -9 (7), -2 (-4 to -1) 10f. -14 (7) vs -10 (8), -3 (-5 to -1) 10g. -11 (7) vs -8 (8), -3 (-6 to -1) 10h. -13 (8) vs -9 (8), -4 (-6 to -1) 10i. -32 (29) vs -21 (29), -11 (-17 to -4) 10j. -38 (27) vs -24 (29), -14 (-22 to -6) 10k. -36 (31) vs -24 (30), -11 (-18 to -4) 10l. -42 (27) vs -27 (30), -15 (-23 to -7) 10m. -0.44 (0.59) vs -0.39 (0.66), -0.05 (-0.19 to 0.09) 10n. -0.56 (0.53) vs -0.49 (0.67), -0.07 (-0.24 to 0.10)  11a. -16 (27) vs -16 (24), -0.3 (-6 to 5) 11b. -22 (27) vs -19 (24), -3 (-10 to 4) 11c. -56 (68) vs -57 (63), 1 (-13 to 16) 11d. -60 (70) vs -69 (60), 8 (-10 to 26) 11e. -11 (8) vs -11 (8), -0.3 (-2; 2) 11f. -13 (7) vs -13 (7), -0.4 (-2; 2) 11g. -10 (9) vs -9 (8), -1 (-3 to 1) 11h. -12 (9) vs -11 (8), -1 (-4 to 1) 11i. -30 (31) vs -22 (28), -8 (-15 to -1) 11j. -37 (29) vs -28 (27), -9 (-16 to -1) 11k. -34 (31) vs -26 (31), -9 (-16 to -1) 11l. -40 (28) vs -30 (28), -10 (-18 to -2) 11m. -0.41 (0.64) vs -0.42 (0.76), 0.01 (-0.15 to 0.17) 11n. -0.55 (0.62) vs -0.54 (0.79), -0.01 (-0.20 to 0.19) | … | + |
| Downs, 2006[73] | Pre-specified; 9-mo follow-up Main outcomes Group 1 (CCDSS) vs 2 (CD-ROM [compact disc – read-only memory]) vs 3 (Workshop) vs 4 (Control) **1. Detection of dementia in patients ≥ 75 y of age: n (%).  2. Concordance with guidelines regarding diagnosis: n, mean (SD) (primary outcome). 3. Concordance with guidelines regarding management: n, mean (SD).**  Note: Pre-intervention detection and concordance rates were also reported; however, authors indicated these were not directly comparable because pre-intervention data were collected for up to 12 years while post-intervention data was collected for 9 months. | 1. 32 (30%) vs 11 (20%) vs 21 (31%) vs 6 (11%); CCDSS vs control, *P=*.01; Workshop vs control, *P=*.01  2. n=32 vs 11 vs 21 vs 6; 3.1 (2.4) vs 3.6 (1.4) vs 3.5 (2.4) vs 3.3 (2.0), *P=*.4 overall  3. n=163 vs 102 vs 112 vs 73; 1.8 (1.4) vs 1.5 (1.4) vs 2.3 (1.5) vs 1.3 (1.3), *P=*.3 overall | ... | … | 0 | … |
| Feldstein, 2006[74] | At 6 months **1. % of participants who received bone mineral density measurement or osteoporosis medication within 6 months of the start of the study; p-value (primary). 1a. provider reminder + patient reminder vs control 1b. provider reminder alone vs control** 1c. provider reminder + patient reminder vs provider reminder alone  2. Change in probability of bone mineral density measurement as predicted by linear model; coefficient (represents absolute change) (95% CI); p-value 2a. Provider reminder + patient reminder vs control  2b. Provider reminder vs control   3. Change in probability of osteoporosis medication prescription as predicted by linear model; coefficient (represents absolute change) (95% CI); p-value 3a. Provider reminder + patient reminder vs control  3b. Provider reminder vs control   4. Change in probability of EITHER bone mineral density measurement or osteoporosis medication prescription as predicted by linear model; coefficient (represents absolute change) (95% CI); p-value 4a. Provider reminder + patient reminder vs control  4b. Provider reminder vs control   5. % of participants who received only bone mineral density measurement within 6 months of the start of the study (component of primary); p-value 5a. provider reminder + patient reminder vs control 5b. provider reminder alone vs control 5c. provider reminder + patient reminder vs provider reminder alone  6. % of participants who received only medication within 6 months of the start of the study (component of primary); p-value 6a. provider reminder + patient reminder vs control 6b. provider reminder alone vs control 6c. provider reminder + patient reminder vs provider reminder alone  Note: n’s for those receiving specified treatment can be calculated from article.  7. Mean change in patient satisfaction with care and service score (electronic medical record plus patient reminders vs electronic medical record reminders alone vs control); p-value (secondary) | 1a. 43.1% vs 5.9%; *P<*.01 1b. 51.5% vs 5.9%, *P<*.01 1c. 43.1% vs 51.5%, *P=*.88 2a. 0.31 (0.21 to 0.43) 2b. 0.39 (0.28 to 0.50)  3a. 0.15 (0.05 to 0.26) 3b. 0.23 (0.12 to 0.33)  4a. 0.38 (0.26 to 0.50) 4b. 0.47 (0.35 to 0.59)  5a. 22.9% vs 0.9%, *P<*.01 5b. 23.8% vs 0.9%; *P<*.01 5c. 22.9% vs 23.8%, *P=*.43  6a. 10.1% vs 4.0%; *P<*.01 6b. 11.9% vs 4.0%; *P<*.01 6c. 10.1% vs 11.9% ; *P=*.54  7. 0.08 vs 0.07 vs -0.07; *P<*.81 | **1. Caloric expenditure per week at baseline, at 6 months; p-value (secondary) 1a. Provider reminder + patient reminder vs control 1b. Provider reminder vs control**  **2. n/N, % of responders participating in regular physical activity at baseline, at 6 months; p-value (secondary) 2a. Provider reminder + patient reminder vs control 2b. Provider reminder vs control**  **3. Total calcium intake (mg/day) baseline, at 6 months; p-value (secondary) 3a. Provider reminder + patient reminder vs control 3b. Provider reminder vs control** | 1a. 2614.4, 2525.9 vs 2325.7, 1980.9; *P=*.32 1b. 3082.9, 2312.7 vs 2325.7, 1980.9; *P=*.96   2a. 11/42, 26.2%, 12/42, 28.6% vs 7/33, 21.2%, 10/33, 30.3%; *P=*.55 2b. 9/41, 22%, 8/41,19.5% vs 7/33, 21.2%, 10/33, 30.3%; *P=*.17  3a. 1221.5, 1224.7 vs 1308.6, 851.2; *P=*.05 3b. 1116.5, 1311.4 vs 1308.6, 851.2; *P=*.02 | + | 0 |
| McDonald, 2005[75] | The pre-determined nurse practitioner outcome measures for intervention (adjusted probability, difference from control (p-value)) vs. augmented intervention (adjusted probability, difference from control (p-value)) vs. control (adjusted probability) for **1. Nurse assessment practices including 1a. presence of pain 1b. presence of pain at every visit 1c. Pain intensity (using numeric scale)  1d. location of pain 1e. Other assessments of pain 1f. Medication assessment 1g. Mood assessment 1h. Bowel movement assessment  2. Nurse instruction practices including 2a. medication management 2b. Side effects of medications 2c. Other pain management instructions 2d. Instruction on contacting physician 2e. Education materials** | 1a. 89.3, 2.4(0.57) vs. 88.0, 1.1(0.81) vs. 86.9 1b. 39.0, 4.0(0.63) vs. 38.1, 3.1(0.53) vs. 35.0 1c. 31.9, 5.7(0.39) vs. 27.9, 1.7(0.80), vs. 26.2 1d. 76.8, -5.5(0.35) vs. 82.4, 0.1(0.99) vs. 82.3 1e. 60.6, 6.3(0.38) vs. 54.8, -0.5(0.94) vs. 54.3 1f. 45.6, 1.1(0.86) vs. 50.4, 5.9(0.39), vs. 44.5 1g. 92.7, 7.2(0.08) vs. 88.9, 3.4(0.48) vs. 85.5  1h. 89.0, -5.7(0.02) vs. 92.0, -2.7(0.26) vs. 94.7   2a.. 34.7, 4.0(0.50) vs. 31.9, 1.2(0.84) vs. 30.7 2b. 10.3, -1.4(0.74) vs. 21.4, 9.7(0.07) vs. 11.7  2c. 16.1, 2.2(0.64) vs. 8.5, -5.4(0.21) vs. 13.9 2d. 7.3, -1.3(0.73) vs. 10.8, 2.2(0.61) vs. 8.6 2e. 2.4, 1.1(0.59) vs. 7.3, 6.0(0.07) vs. 1.3 | The pre-determined patient outcome measures for intervention (adjusted probability/score, difference from control (p-value)) vs. augmented intervention (adjusted probability/score, difference from control (p-value)) vs. control (adjusted probability/score) for **1. pain 1a. pain at its worst (range 0 -10) 1b. Pain on average (range 0-10) 1c. Pain interference scale (range 0-10)  2. European Organization for Research and Treatment of Cancer questionnaire (higher values = better overall outcome but worse outcome on symptom scales) 2a. Best quality of life (scale >74) 2b. Severe pain (scale >74) 2c. Severe insomnia (scale >74) 2d. Severe constipation (scale >74)  3. Symptom management 3a. Inadequate pain management 3b. Barriers summary score 3c. Use of alternative treatments**  **4. Cost effectiveness for home care-related cost of a 10% reduction in (US$) for basic intervention; augmented intervention for**  **4a. Pain at its worst.**  **4b. Pain on average.**  **4c. Probability of hospitalization.** | 1a. 3.6, -0.9(0.13) vs. 3.3,-1.2(0.05) vs. 4.5  1b. 2.2, -1.5(0.03) *P<*.05) vs. 3.1, -0.6(0.42) vs. 3.7  1c. 5.8, 0.5(0.11) vs. 5.2,-0.1(0.86) vs. 5.3  2a. 16.9%, 0.8(0.79) vs. 15.2%, -0.9(0.81) vs. 16.1%  2b. 32.0%, 3.6(0.44) vs. 25.8%, -2.6(0.57) vs. 28.4%  2c. 39.5%, -1.4(0.79) vs. 32.8%, -8.1 (0.15) vs. 40.9%  2d. 14.8%, -4.1(0.27) vs. 12.0%, -6.9(0.08) vs. 18.9%  3a. 69.9%, 1.4(0.70) vs. 64.0%, -4.5(0.29) vs. 68.5%  3b. 37.6, -0.1(0.98) vs. 39.0, 1.3(0.81) vs. 37.7  3c. 22.6%, -4.3(0.22) vs. 15.9%, -11.0 (0.02) vs. 26.9%  4a. Not effective in improving outcome; 97  4b. 37; not effective in improving outcome  4c. Not effective in improving outcome; 466 | 0 | 0 |
| Dexter, 1998[76] | **Pre-specified - rate of discussions and rate of form completion**  **1. Rate (%) of advance directive discussions at 1 year; OR (95%CI)**  **1a. Instruction directives and proxy directive reminders**  1b. Instruction directive reminders  1c. Proxy directive reminders  **2. Rate (%) form completion of either directive at 1 year; OR (95%CI)**  **2a. Instruction directives and proxy directive reminders**  2b. Instruction directive reminders  2c. Proxy directive reminders | 1a. 24 vs 4; 7.7(3.4-18)  1b. 14 vs 4; 4.4(2.1-9.4)  1c. 8 vs 4; 2.5(1.1-5.5)  2a. 15 vs 4; 7.0(2.9-17)  2b. 7 vs 4; 3.0(1.1-8.0)  2c. 3 vs 4; 1.0(0.4-2.7) | … | … | + | … |
| Rubenstein, 1995[77] | **1. Mean (SD) and difference (95%CI) for number of clinical problems per patient in medical records during 6 month follow-up that were listed in the visit (prespecified).**  **2. Mean (SD) and difference (95%CI) for number of functional status interventions per patient with functional status problems during 6 month follow-up (prespecified). 3. Functional status interventions during 6 month follow-up (prespecified).  3a. Total number.  3b. Proportion of interventions recommended in study materials.  4. Physician attitudes toward managing functional status at end of study (prespecified).** | 1. 4.9 (3.4) vs 4.1 (2.9); 0.8 (0.2 to 1.5), *P<*.01  2. 3.3 (3.7) vs 2.5 (3.3); 0.8 (0.1 to 1.6), *P=*.05 3a. 231 vs 95 3b. 81% vs 71%; 10% (1 to 19), *P<*.02 4. Data not reported. | **1. Mean change (difference, 95% CI) in patient functional status during 6 month follow-up (scale 0-100, 100=highest performance). (predefined) 1a. Basic activities of daily living. 1b. Intermediate activities of daily living. 1c. Mental health. 1d. Social activities. 1e. Work performance.** 2. Mean change adjusted by baseline scores (difference, 95% CI) in social activities scores by age group over 6 month follow-up (unclear if analysis by age groups was preplanned).  2a. <50 years of age. 2b. 50-69 years of age. 2c. >69 years of age.  **3. % patients (difference, 95%CI) identified as having specific impairments during 6 months follow-up (predefined). 3a. Physical, psychological, or social function impairment.** 3b. Depression or anxiety. 3c. Depression. 3d. Anxiety. 3e. Social problems. 3f. Physical function impairments. | 1a. 0.5 vs 0.1; 0.44 (-3.2 to 4.1), *P=*.81 1b. 0.9 vs 1.1; -0.2 (-4.6 to 4.2), *P=*.92 1c. 1.3 vs -3.2; 4.5 (0.5 to 8.3), *P=*.03 1d. 3.3 vs -1.5; 4.8 (-0.8 to 10.4), *P=*.09 1e. 0.2 vs -0.8; 1.0 (-4.4 to 6.6), *P=*.70  2a. 1.13 (n=83) vs 4.49 (n=79); -3.36 (-9.0 to 2.3), *P=*.24 2b. 1.96 (n=47) vs -8.31 (n=42); 10.27 (-1.8 to 22.3), *P=*.10 2c. 9.50 (n=40) vs -10.09 (n=22); 19.59 (1.96 to 36), *P=*.03 Interaction for intervention by age, *P<*.01  3a. 37% vs 25%; 12% (2 to 2.1), [CIs are not consistent with data tho no significant p-value reported for this comparison]  3b. 30% vs 21%; 9% (1 to 20), *P<*.05 3c. 23% vs 20%, 3% (-5 to 12), *P*=NS 3d. 13% vs 4%; 9% (3 to 15), *P<*.01 3e. 17% vs 10%; 7% (0 to 15), *P<*.10  3f. 6% vs 5%; 1% (-4 to 5), *P*=NS | … | 0 |
| Petrucci, 1991[78] | 1. (prespecified) Nurses’ knowledge of caring for patients with urinary incontinence. **2. Nurses’ knowledge of caring for patients with urinary incontinency – group by time interaction** | 1. F.001(2,157)=19.46 (significant) 2. "knowledge of nurses on the treatment group improved gradually over the first 5 weeks of the study and accelerated during the second 5 weeks of the study" F.001(6,157)=45.29 (significant) | 1. (prespecified) Mean number of wet occurrences per week for 10 weeks by group. **2. Mean number of wet occurrences per week for 10 weeks – group by time interaction.**  (data provided in figure) | 1. (figure indicates that treatment groups were drier than control group) F.001(2,81)=34.67 (significant) 2. F.001(18,81)=28.6 (significant)  States that the above (#2) was the only significant interaction but do not report others) | + | + |
| McDonald, 1984[79] | **Main outcome 1. Mean per-patient response to reminders over 2 years (%).  1a. For 115 residents.  1b. For 11 faculty.  1c. For 4 nurse-clinicians.**  Not clearly prespecified 2. Residents per-patient response to reminders over 2 years.  2a. Digitalis usage.  2b. Occult blood.  2c. Cervical smear.  2d. Haematocrit.  2e. Chest roentgenogram.  2f. Pneumococcal vaccine.  2g. Tuberculosis skin test.  2h. Serum K.  2i. Mammography.  2j. Influenza vaccine.  2k. Diet.  2l. Reticulocytes.  2m. Iron/Iron binding.  2n. Liver enzymes.  2o. Antacids.  2p. Other.   Note: physicians with <100 reminder messages during study were excluded from analysis, and for the 15 most frequent reminders, physicians with <6 eligible patients for an action were excluded from analysis (p.132 of article).  Note: Data inconsistencies. a. P-value for digitalis reminder = .015 in figure 2 and .15 in text (p. 134).  b. *P*-values reported for 15 actions on p.134 but only 14 actions listed.  3. For less common reminders: 3a. serum amylase for abdominal pain 3b. colon roentgenograms for Hb-positive stools 3c. urine cultures for pyurea 3d. serum fluorescent treponemal antibody tests to follow-up positive venereal disease research laboratory tests 3e. median cell volumes to detect anaemia 3f. metronidazole to treat trichomonas 3g. multivitamins for alcoholic patients 3h. vitamin K for unexplained prothrombin time elevations 3i. prothrombin time after Coumadin treatment 3j. T4 index to work up findings suspicious of hypo- or hyperthyroidism  4. Response rate (group mean response to an indication for a clinical action) amongst residents 4a. Occult blood  4b. Cervical smear 4c. Chest roentgenogram 4d. Pneumococcal vaccine 4e. Tuberculosis skin test 4f. Serum potassium 4g. Mammography 4h. Influenza vaccine  4i. Diet  4j. Digitalic 4k. Antacids 4l. β-blockers | 1a. 49% vs 29%, *P<*.001 1b. 44% vs 29%, *P<*.01 1c. 50% vs 36%, *P<*.03 paired t-test, *P<*.06 Wilcoxon signed rank test)   2. No data reported; figure 2 shows higher rates in study group for all reminders. All in favour of CCDSS group.  2a. *P=*.015 (not significant at Bonferroni correction level, 0.0033) 2b. *P<*.001  2c. *P<*.001 2d. *P<*.001 2e. *P<*.001 2f. *P<*.001 2g. *P<*.001 2h. *P<*.001 2i. *P<*.001 2j. *P<*.001 2k. *P<*.001 2l. *P<*.001 2m. *P<*.001 2n. *P<*.001 2o. *P<*.001 2p. *P<*.001   3. *P<*.001 in favour of CCDSS.  No data reported, but article specifies large CCDSS effects for the less common reminders listed.  4a. 0.55 vs 0.22 4b. 0.38 vs 0.23 4c. 0.43 vs 0.30 4d. 0.51 vs 0.14 4e. 0.26 vs 0.03 4f. 0.84 vs 0.75 4g. 0.08 vs 0.02 4h. 0.46 vs 0.20 4i. 0.49 vs 0.37 4j. 0.49 vs 0.37 4k. 0.50 vs 0.37 4l. 0.15 vs 0.14 | Prespecified interest in patient outcomes but not which outcomes. Follow-up at 2 years.   **1. Number of hospitalizations, emergency room visits, and clinic visits.  2. Time averaged values for DBP/SBP, weight, serum glucose, serum Hb, serum potassium, and blood urea nitrogen.**  3. Winter hospitalizations and emergency room visits in patient subgroup eligible for influenza or pneumococcal vaccine. | 1. Data not reported, *P*=NS  2. Data not reported, *P*=NS  3. Data not reported, *P<*.02 in favour of CCDSS group | + | 0 |

Abbreviations: ACE –I, angiotensin converting enzyme inhibitor; ARB, angiotensin receptor blockers; BMI, body mass index; BP, blood pressure; CAD, coronary artery disease; CCDSS, computerized clinical decision support system; CHD, coronary heart disease; CI, confidence interval; COPD, chronic obstructive pulmonary disease; CV(D), cardiovascular (disease); DBP, diastolic blood pressure; Hb, haemoglobin; HDL-C, high-density lipoprotein cholesterol; HR, hazard ratio; IQR, interquartile range; ITT, intention to treat; LDL-C, low-density lipoprotein cholesterol; NR, not reported; NS, not significant; NSAID, non-steroidal anti-inflammatory drug; OR, odds ratio; RR, risk ratio; SBP, systolic blood pressure; SD, standard deviation; SE(M), standard error (of the mean); SF-36, Short-form 36 questionnaire; VAS, visual analogue scale.

^a^Ellipses (…) indicate item was not assessed or could not be evaluated. Outcomes in bold font were assessed for effect.

^b^Outcomes are evaluated for effect as positive (+) or negative (−) for CCDSS, or no effect (0), based on the following hierarchy. An effect is defined as ≥ 50% of relevant outcomes showing a statistically significant difference (2*P* < .05):

- If a single primary outcome is reported, *in which all components are applicable*, this is the only outcome evaluated (see Methods section of manuscript for definition of primary outcome).
- If > 1 primary outcome is reported, the ≥ 50% rule applies and only the primary outcomes are evaluated.
- If no primary outcomes are reported (or only some of the primary outcome components are relevant) but overall analyses are provided, the overall analyses are evaluated as primary outcomes. Subgroup analyses are not considered.
- If no primary outcomes or overall analyses are reported, or only some components of the primary outcome are relevant for the application, any reported prespecified outcomes are evaluated.
- If no clearly prespecified outcomes are reported, any available outcomes are considered.
- If statistical comparisons are not reported, ‘effect’ is designated as not evaluated (…).

^c^Study included in 2 categories.
