# Supplementary material for: Computerized clinical decision support systems for chronic disease management: A decision-maker-researcher partnership systematic review
Source: Implement Sci. 2011 Aug 3;6:92. doi: 10.1186/1748-5908-6-92 (PMC3170626; doi:10.1186/1748-5908-6-92)
Supplement: Additional file 5 — Table S5. Costs and CCDSS process-related outcomes for trials of chronic disease management. Cost and CCDSS process-related outcomes for the included studies. [file 1748-5908-6-92-S5.DOCX]

**Additional file 5, Table S5. Costs and CCDSS process-related outcomes for trials of chronic disease management^a^**

| **Study** | **CCDSS adverse effects** | **Costs^b^** | **Group comparison for CCDSS workflow** | **Practitioner satisfaction with CCDSS system** |
| --- | --- | --- | --- | --- |
|  |  | **Diabetes** |  |  |
| Holbrook, 2009[2, 3] | ... | ... | ... | Many providers indicated that technical difficulties with the CCDSS had a negative impact on the perceived usefulness of the system. However, 16/33 (48%) felt they improved their knowledge of diabetes targets, 11/33 (33%) felt their patients adherence to appointments improved, and 12/33 (36%) felt patient access to high-quality diabetes care improved. |
| Maclean, 2009[11, 12] | ... | Estimated total costs of care per person per year: $3,202 vs $4,937; adjusted difference, -$2,426 (95% CI -$4,647 to -$205), *P*=.03. Ongoing information technology support estimated at < 1 hour/month. | ... | ... |
| Christian, 2008[13] | ... | ... | ... | Author reported physicians were very satisfied with the system and intervention process. |
| Cleveringa, 2008[14-17] | ... | 1. diabetes-related costs (excluding CHD) (Euros, €): difference between CCDSS and control (95% CI) | ... | ... |
|  |  | 1a. all patients: 1698 (187-3209) |  |  |
|  |  | 1b. patients with history of CVD: 1167 (-620-2954) |  |  |
|  |  | 1c. patients without history of CVD: 2146 (-189-4482) |  |  |
|  |  | 2. CHD costs (€): difference between CCDSS and control (95% CI) |  |  |
|  |  | 2a. all patients: -587 (-880 to -294) |  |  |
|  |  | 2b. patients with history of CVD: -433 (-847 to -18) |  |  |
|  |  | 2c. patients without history of CVD: -721 (-1177 to -265) |  |  |
|  |  | 3. Diabetes Care Protocol costs (€): difference between CCDSS and control (95% CI) |  |  |
|  |  | 3a. all patients: 316 (315-318) |  |  |
|  |  | 3b. patients with history of CVD: 314 (3112-316) *seems to be error in paper |  |  |
|  |  | 3c. patients without history of CVD: -319 (318-320) |  |  |
|  |  | 4. Total costs (€): difference between CCDSS and control (95% CI) |  |  |
|  |  | 4a. all patients: 1415 (-130-2961) |  |  |
|  |  | 4b. patients with history of CVD: 1037 (-891-2967) |  |  |
|  |  | 4c. patients without history of CVD: -1698 (-692-4089) |  |  |
| Peterson, 2008[18] | ... | Costs: 1 hour/provider for coordinators, 1 hour/month for local physician champions, software for 67% of sites, computer + printer for 33% of sites. | ... | No formal measure of practitioner satisfaction; however, author reported that a majority appeared satisfied with the system. |
| Quinn, 2008[19] | ... | ... | ... | All physicians reported that they would recommend the WellDoc System to other patients and that it was easy to use, facilitated treatment decisions, and would improve efficiency of office visits. |
| Lobach, 1997[23] | ... | ... | No difference in encounter length when CCDSS was or was not supplied (*P*>.10). | ... |
| Thomas, 1983[26] | ... | Ambulatory care costs for patients with chart audit system (n=58) vs those with no chart audit system (n=75) over 12 months including: | ... | ... |
|  |  | 1. Office visits. $8712 vs $12269 |  |  |
|  |  | 2. Laboratory, X ray, electrocardiogram, etc. $5884 vs $6925 |  |  |
|  |  | 3. Procedures. $1832 vs $2260 |  |  |
|  |  | 4. Emergency centre. $3543 vs $3037 |  |  |
|  |  | 5. Total. $19971 vs $24491 |  |  |
|  |  | 6. Average cost/patient. $344 vs $327 |  |  |
|  |  | 7. Total Hospitalization cost. $92378 vs $260990 |  |  |
|  |  | 8. Cost/Hospitalization. $4619 vs $6366 |  |  |
|  |  | 9. Outpatient costs. $19971 vs $24491 |  |  |
|  |  | 10. Inpatient Cost. $92378 vs $260990 |  |  |
|  |  | 11. Total. $112349 vs $285481 |  |  |
|  |  | 12. Average cost per patient/year. $1937 vs $3806 |  |  |
|  |  | 13. Annual cost of processing the automated record and audit system for |  |  |
|  |  | 13a. medical technicians (1/3 time). $4400 |  |  |
|  |  | 13b. Data entry equipment. $1128 |  |  |
|  |  | 13c. Supplies. $720 |  |  |
|  |  | 13d. Computer (offline). $1967 |  |  |
|  |  | 13e. Total cost for 305 patients. $8215  13f. Cost/patient visit for 12-month study. $5.77 |  |  |
|  |  | 13f. Cost/patient visit for 12-month study. $5.77 |  |  |
|  |  | **Diabetes and Other** |  |  |
| Sequist, 2005[28] | ... | ... | ... | 71% of physicians preferred the electronic decision support over a paper-based system and 76% thought that the system helped to improve quality of care. Of the physicians in the intervention group, 68% found electronic reminders for diabetes care useful; 53% found them useful for coronary artery disease management. |
| Martin, 2004[29] | ... | Cost of intervention, $10.50 per member per month; $189 per member over 18 months of study. | ... | ... |
|  |  | 1. Mean (SE) costs measured as paid claims for the 18 month study period. |  |  |
|  |  | 1a. Medical cost per member. $6828 (230) vs $7001 (249); *P*=.61 |  |  |
|  |  | 1b. Intervention cost per member. $189 vs $0; Not applicable |  |  |
|  |  | 1c. Total mean cost per member. $7017 vs $7001; Not applicable |  |  |
|  |  | 1d. Pharmacy costs per member per month. $56.39 vs $57.20 |  |  |
|  |  | 1e. Cost of members lost through death or disenrollment. No difference (no data reported). |  |  |
| Hetlevik, 1999[31-33] | ... | ... | ... | 1. Physicians reported the CCDSS provided some or much benefit for checking blood pressure (68%); taking history (67%); diagnostic support, lab tests, lifestyle advice (oral), and infarction risk score calculation (61%); lifestyle advice (out-prints) (56%); clinical exams (53%), and treatment indications (50%). |
|  |  |  |  | 2. Physician evaluation of CCDSS user-friendliness (agreed or partly agreed). |
|  |  |  |  | 2a. One CCDSS for 3 diagnoses as a good/acceptable solution (20/24, 83%) |
|  |  |  |  | 2b. CCDSS too large (22/24, 92%) |
|  |  |  |  | 2c. Recommended procedures too time-consuming (20/24, 83%) |
|  |  |  |  | 2d. Many recommendations were unnecessary (12/24, 50%) |
|  |  |  |  | 2e. Could remember procedures without the CCDSS (16/24, 67%) |
|  |  |  |  | 3. Physicians reported some CCDSS implementation strategies were of some or large benefit: |
|  |  |  |  | 3a. Ready to use (11/21, 52%) |
|  |  |  |  | 3b. Physician training (17/21, 81%) |
|  |  |  |  | 3c Assistant training (10/18, 56%) |
|  |  |  |  | 3d. Physician user manual (11/21, 52%) |
|  |  |  |  | 3e. Telephone repetitions for physicians (15/21, 71%) |
|  |  |  |  | 3f. Attending risk intervention seminar (2/2, 100%) |
|  |  |  |  | Strategies of little of no use: |
|  |  |  |  | 3g. Assistant user manual (10/19, 53%) |
|  |  |  |  | 3h. Report of own patients with diabetes (11/21, 52%) |
|  |  |  |  | 3i. Checking use of CCDSS (14/20, 70%) |
|  |  |  |  | 3j. Visiting the CCDSS stand (2/2), 100%). |
|  |  | **Hypertension** |  |  |
| Borbolla, 2007[36] | Author commented that no adverse effects were found. | ... | ... | Author commented that informal evaluations found practitioners were satisfied. |
| Murray, 2004[38] | ... | 1. % direct health care charges (SD) | ... | ... |
|  |  | 1a. Outpatient. 3005 (4318) vs 2868 (3553) vs 2681 (3520) vs 2229 (2137); NS |  |  |
|  |  | 1b. Inpatient. 2145 (9805) vs 2577 (7709) vs 3519 (17830) vs 893 (3450); NS |  |  |
|  |  | 1c. Total health care charges. 5149 (11756) vs 5445 (9612) vs 6200 (18947) vs 3122 (4633); NS |  |  |
|  |  | **Asthma and COPD** |  |  |
| Kattan, 2006[50] | ... | Estimated cost of the intervention was $69.20 per child over the year. Total cost savings was $337.00 per child in the intervention group. Monte Carlo simulations, using the observed distributions of symptom days and resource use, showed that the intervention had a 97% chance of being cost saving. Hourly wage of $15 for a clerical employee was used in the calculation. There were 6 calls per child per year (40 mins per call) resulting in a cost of $60. The cost for these materials on a per child basis was $9.20. | ... | ... |
| Plaza, 2005[52] | ... | Direct and indirect costs were calculated. The former was calculated as the product of the consumption of each resource times its unitary cost. Treatment costs were estimated using drug market prices in Spain. The remaining costs were obtained through the SOIKOS database with prices corrected for year 2001 and using average prices (medical visits: 8.47 €; home visits:19.53 €; emergency visits: 87.53 €; days in intensive care unit: 1,156.44 €; shift days: 282.46 €; espirometries: 10.03 €; blood standard analysis: 10.73 €; total E immunoglobulin: 6.77 €, thorax radiographies: 14.35 €; skin allergic tests: 30.05 €). Indirect costs were calculated, for active workers, as the product of hours lost and the total cost per hour of effective work (11.79 €). | ... | ... |
|  |  | The total costs were estimated using a social and national healthcare system perspective. From a social perspective the medical costs per patient rose to 2444.35 (987 treatments) in the usual care group and 1408 (833 treatments) in intervention group. The estimated difference was -1022 (95%IC -2165-122; *P*=.08). |  |  |
|  |  | From the perspective of the National Health Care System, the total median medical cost per patient rose 1544 (891 treatments) in the usual care group and to 1077 (767 treatments) in intervention group. The difference was -489 (95%IC -1302-324; *P*=.24). |  |  |
| Tierney, 2005[53] | ... | 1. Mean (SD) direct health care charges over 12 months (US $). All *P* = NS unless noted otherwise: Physician intervention vs pharmacist intervention vs both interventions vs control | ... | ... |
|  |  | 1a. Outpatient charges. 3,142 (3,381) vs 2,814 (3,282) vs 3,177 (3,558) vs 3,129 (2,921) |  |  |
|  |  | 1b. Inpatient charges. 4,864 (17,257) vs 2,519 (7,267) vs 2,475 (8,699) vs 2,671 (6,805) |  |  |
|  |  | 1c. Total health care charges. 8,006 (18,720) vs 5,333 (9,400) vs 5,652 (10,579) vs 5,800 (8,536), *P*<.05 for increase with physician intervention. |  |  |
| Eccles, 2002[54, 55]^c^ | ... | ... | ... | Author comment: We have always interpreted Figure 3 (shows the number of times the guidelines were triggered for each practice and the proportion of active interactions that involved going beyond the first screen - the median number of active interactions was zero for much of the study) to mean that the majority of the users were not satisfied - in that they stopped using the system. This is certainly what the process evaluation suggested. |
| McCowan, 2001[56] | ... | All 9 responders said the CCDSS could be used in consultations lasting ≤ 10 minutes, although users indicated it had slightly increased consultation times. | ... | Users found the software easy to use. Management recommendations and reminders were popular with users, who felt they contributed to improved quality of consultations. The risk prediction data was not popular within the consultation. Printed management plans were useful and of value to patients. |
|  |  | **Dyslipidaemia** |  |  |
| Bertoni, 2009[57, 58] | Patients had a greater risk for overtreatment than of undertreatment because all patients were screened including low risk patients who would not normally be screened. | ... | ... | ... |
| Gilutz, 2009[59] | Author comment: no adverse effects. | Author comment: yes it was cost effective; data not stated. The cost of implementing the CCDSS was $170000 | ... | Author commented that 143 satisfaction grading forms were available with 91% rate of general satisfaction. |
| Lester, 2006[60, 61] | ... | Median time to complete an email notification: 90 sec (range 15 sec to 49 min). | ... | ... |
| Cobos, 2005[62] | ... | Direct costs = sum of costs of physician visits, laboratory analyses, and lipid lowering drugs prescribed during the study. For each patient, visit and laboratory costs were estimated by frequency x unit cost (physician visit 12€, lipid 9.46€, alanine aminotransferase and aspartate amintransferase 2€ each, creatine kinase 1€). All costs in €s. | ... | ... |
|  |  | 1. Lipid-lowering drug treatment costs at 1 year. 125,569 vs 214,683 |  |  |
|  |  | 2. Lipid-lowering drug total costs at 1 year. 170,061 vs 264,658 |  |  |
|  |  | 3. Adjusted means for treatment costs per patient; difference (95% CI); savings %. 178 vs 237; 59 (34-83, *P*<.001); 24.9%. |  |  |
|  |  | 4. Adjusted means for total costs per patient; difference (95% CI); savings %. 223 vs 283; 60 (33-86), *P*=.001; 20.8% |  |  |
|  |  | **Cardiac Care** |  |  |
| Goud, 2009[63, 64] | ... | ... | Time increase caused by cardiac rehabilitation decision support system, minutes: median (interquartile range). 10 (0-10). | Questionnaire returned by 63 of 68 (93%) of rehab professionals. Mean (SD) responses (7-point Likert responses, 7=high satisfaction): Overall usability, 5.10 (0.85); Ease of system use, 5.14 (1.08); Information quality, 5.05 (0.87); Interface quality, 5.10 (1.18) |
| Feldman, 2005[65, 66] | ... | Service use is reported under practitioner performance outcomes and cost-effectiveness under patient outcomes. | ... | ... |
|  |  | 1. Mean home care-related costs: Augmented intervention (AI) $3,425 vs usual care (UC) $2,814, *P*=.06; Basic intervention (BI) $3,371 vs UC, *P*=.06 |  |  |
|  |  | 2. Mean overall costs: AI $6,330 vs UC $4,996, *P*=.02; BI $5,869 vs UC, *P*=.08 |  |  |
| Tierney, 2003[67] | …. | Mean (SD) direct health care charges per patient over 1 year: physician intervention vs pharmacist intervention vs both interventions vs control. | ... | ... |
|  |  | 1a. Outpatient charges. $2,961 (2,795) vs $3,143 (3,124) vs $3,176 (2,974) vs $2,696 (3,363) |  |  |
|  |  | 1b. Inpatient charges. $3,341 (9,162) vs $4,245 (11,544) vs $4,463 (15,217) vs $4,330 (14,461) |  |  |
|  |  | 1c. Total charges. $6,302 (10,928) vs $7,387 (13,206) vs $7,639 (16,921) vs $7,025 (17,024) |  |  |
| Eccles, 2002[54, 55] ^c^ | ... | ... | ... | Author comment: We have always interpreted Figure 3 (shows the number of times the guidelines were triggered for each practice and the proportion of active interactions that involved going beyond the first screen - the median number of active interactions was zero for much of the study) to mean that the majority of the users were not satisfied - in that they stopped using the system. This is certainly what the process evaluation suggested. |
|  |  | **Other** |  |  |
| Javitt, 2008[71] | ... | Outcomes over 1 year. | ... | ... |
|  |  | 1. Change in costs $ of medical care from previous year relative to control group; $ (t-statistic).*P* value |  |  |
|  |  | 1a. Total charges (per member per month [pmpm]). −21.92 (1.99); *P*<.05 (6.1% reduction in mean total charges from mean control group charges of $352 pmpm). |  |  |
|  |  | 1b. Inpatient charges (pmpm). −12.833 (1.8); *P*=.10 |  |  |
|  |  | 1c. Out-patient charges (pmpm). −1.823 (0.60); NS |  |  |
|  |  | 1d. Medication charges (pmpm). 0.7 (0.90); NS |  |  |
|  |  | 1e. Professional charges (pmpm). −7.963 (2.20); *P*<.05 |  |  |
|  |  | 1f. In hospital (pmpm). 0.000 (0.10); NS |  |  |
|  |  | 2. Mean reimbursement differentials (hospital charged health maintenance organization [HMO] for all services but was reimbursed at a fixed rate): -$8.96 overall (6% drop in CCDSS group), mostly due to professional reimbursements (-$4.62) and mean in-patient charges (-$3.88). |  |  |
|  |  | 3. Charge differentials: |  |  |
|  |  | 3a. Patients at the median. $0.561 pmpm |  |  |
|  |  | 3b. Patients at the 90th percentile. -$26.512 pmpm |  |  |
|  |  | 3c. Patients at the 99th percentile. -$658.612 pmpm |  |  |
|  |  | 4. Change in costs $ of medical care in subgroup of patients > 50 years of age at baseline (intervention vs control); $ (t-statistic). |  |  |
|  |  | 4a. Total charges (per member per month [pmpm]). -$72.171 (2.04) |  |  |
|  |  | 4b. In-patient charges (pmpm). -$49.633 (2.12) |  |  |
|  |  | 4c. Out-patient charges (pmpm). -$9.59 (1.10) |  |  |
|  |  | 4d. Medication charges (pmpm). $0.447 (0.13) |  |  |
|  |  | 4e. Professional charges (pmpm). -$13.395 (1.18) |  |  |
|  |  | 5. Change in costs $ of medical care in all patients (weighted by propensity score for risk of receiving a patient-specific care consideration based on pre-study characteristics), (intervention vs control); $ (t-statistic). |  |  |
|  |  | 5a. Total charges (per member per month [pmpm]). -$66.363 (3.44) |  |  |
|  |  | 5b. In-patient charges (pmpm). -$38.067 (3.5) |  |  |
|  |  | 5c. Out-patient charges (pmpm). -$2.913 (0.57) |  |  |
|  |  | 5d. Medication charges (pmpm). -$0.821 (0.46) |  |  |
|  |  | 5e. Professional charges (pmpm). -$24.562 (2.86) |  |  |
| Verstappen, 2007[72] | ... | ... | ... | Author reports that majority of practitioners were satisfied with system, as indicated in questionnaire. |
| McDonald, 2005[75] | ... | Estimates of Service Use and Cost for basic intervention (adjusted probability/score, difference from control (*P* value), vs Augmented intervention (adjusted probability/score, difference from control (p-value)) vs control group (adjusted probability/score) for | ... | ... |
|  |  | 1. service use |  |  |
|  |  | 1a. Probability of hospitalization, %: 22.1, -0.1(.97) vs. 16.6, -5.6 (.08) vs. 22.2 |  |  |
|  |  | 1b. Probability of emergency department use, %: 37.8, 1.2 (.96) vs. 33.5, -3.1 (.38) vs. 36.6 |  |  |
|  |  | 2. Cost measure for |  |  |
|  |  | 2a. Home care – related costs, US $: 2789, 147 (.55) vs. 2903, 261 (.33) vs. 2642 |  |  |
|  |  | 2b. Overall costs, US$: 5966, 279 (.57) vs. 5611, -76 (.88) vs. 5687 |  |  |

Abbreviations: CCDSS, computerized clinical decision support system; CHD, chronic heart disease; CI, confidence interval; COPD, chronic obstructive pulmonary disease; CVD, cardiovascular disease; NS, not significant; SD, standard deviation; SE, standard error.

^a^Ellipses (…) indicate outcome was not assessed.

^b^Costs include workflow measures, such as time to process alerts, if these are not directly compared between groups.

^c^Study included in 2 categories.
